# Differential cortical activation patterns: pioneering sub-classification of tinnitus with and without hyperacusis by combining audiometry, gamma oscillations, and hemodynamics

**DOI:** 10.3389/fnins.2023.1232446

**Published:** 2024-01-04

**Authors:** Jakob Wertz, Lukas Rüttiger, Benjamin Bender, Uwe Klose, Robert S. Stark, Konrad Dapper, Jörg Saemisch, Christoph Braun, Wibke Singer, Ernst Dalhoff, Katharina Bader, Stephan M. Wolpert, Marlies Knipper, Matthias H. J. Munk

**Affiliations:** ^1^Department of Otolaryngology, Head and Neck Surgery, Tübingen Hearing Research Centre, University of Tübingen, Tübingen, Germany; ^2^Department of Diagnostic and Interventional Neuroradiology, University of Tübingen, Tübingen, Germany; ^3^Department of Psychiatry and Psychotherapy, University of Tübingen, Tübingen, Germany; ^4^Department of Biology, Technical University Darmstadt, Darmstadt, Germany; ^5^MEG-Center, University of Tübingen, Tübingen, Germany; ^6^Section of Physiological Acoustics and Communication, Department of Otolaryngology, Head and Neck Surgery, University of Tübingen, Tübingen, Germany

**Keywords:** tinnitus, hyperacusis, rs-fMRI, fNIRS, EEG, gamma oscillations, distress, attention

## Abstract

The ongoing controversies about the neural basis of tinnitus, whether linked with central neural gain or not, may hamper efforts to develop therapies. We asked to what extent measurable audiometric characteristics of tinnitus without (T) or with co-occurrence of hyperacusis (TH) are distinguishable on the level of cortical responses. To accomplish this, electroencephalography (EEG) and concurrent functional near-infrared spectroscopy (fNIRS) were measured while patients performed an attentionally demanding auditory discrimination task using stimuli within the individual tinnitus frequency (fTin) and a reference frequency (fRef). Resting-state-fMRI-based functional connectivity (rs-fMRI-bfc) in ascending auditory nuclei (AAN), the primary auditory cortex (AC-I), and four other regions relevant for directing attention or regulating distress in temporal, parietal, and prefrontal cortex was compiled and compared to EEG and concurrent fNIRS activity in the same brain areas. We observed no group differences in pure-tone audiometry (PTA) between 10 and 16 kHz. However, the PTA threshold around the tinnitus pitch was positively correlated with the self-rated tinnitus loudness and also correlated with distress in T-groups, while TH experienced their tinnitus loudness at minimal loudness levels already with maximal suffering scores. The T-group exhibited prolonged auditory brain stem (ABR) wave I latency and reduced ABR wave V amplitudes (indicating reduced neural synchrony in the brainstem), which were associated with lower rs-fMRI-bfc between AAN and the AC-I, as observed in previous studies. In T-subjects, these features were linked with elevated spontaneous and reduced evoked gamma oscillations and with reduced deoxygenated hemoglobin (deoxy-Hb) concentrations in response to stimulation with lower frequencies in temporal cortex (Brodmann area (BA) 41, 42, 22), implying less synchronous auditory responses during active auditory discrimination of reference frequencies. In contrast, in the TH-group gamma oscillations and hemodynamic responses in temporoparietal regions were reversed during active discrimination of tinnitus frequencies. Our findings suggest that T and TH differ in auditory discrimination and memory-dependent directed attention during active discrimination at either tinnitus or reference frequencies, offering a test paradigm that may allow for more precise sub-classification of tinnitus and future improved treatment approaches.

## Graphical abstract

Graphical abstract reporting electrical, oscillatory and hemodynamic responses and activities along subcortical (AN, CN, SOC, and IC) and cortical auditory pathways (Auditory cortex—T7, ventrolateral prefrontal cortex—F7, dorsolateral prefrontal cortex—F3, left temporoparietal junction—P3, and right temporoparietal junction—P4). Results are reduced to the arrows indicating the direction of parameter change when comparing T or TH to controls (C) or among each other. Group differences in the oxy-and deoxy-Hb, are shown as more prominent symbols (greater brain activity) and smaller symbols (lesser brain activity). The brain areas described with rs-fMRI-bfc are represented by gray arrows emanating from AC/T7. For abbreviations, see Glossary.
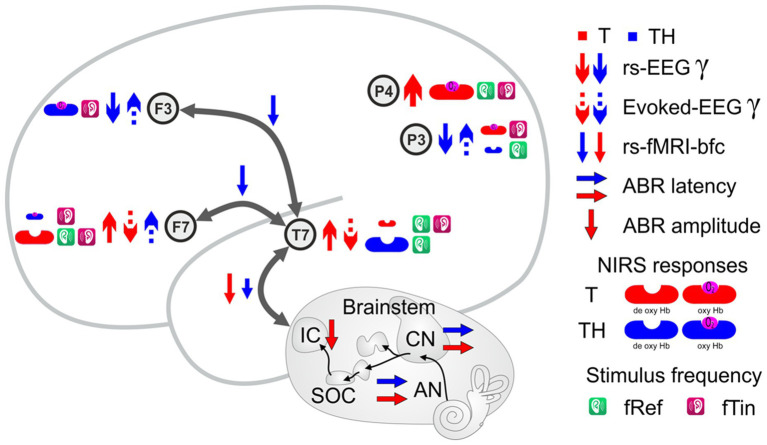

## Introduction

While previous views suggested a homeostatic increase in neural gain to generate central hyper-excitability leading to tinnitus (Shore et al., 2016), we and others observed that tinnitus occurs when the reduced auditory input fails to increase neural gain due to diminished stimulus-evoked responses (Rüttiger et al., 2013; Hofmeier et al., 2018; Möhrle et al., 2019). Previously, a co-morbidity of tinnitus and hyperacusis, which occurs in ~50% of the tinnitus cases (Aazh and Moore, 2018; Cederroth et al., 2020), has been suggested to obscure the identification of the pathogenic neural processes leading to tinnitus (Knipper et al., 2020, 2021). Combining audiometry [pure-tone audiometry (PTA), auditory brainstem response (ABR) wave analysis] with functional imaging assays [evoked and resting state blood oxygenation level-dependent (BOLD) functional magnetic resonance imaging (fMRI) activity of predefined regions of interest (ROI)] allowed for the differentiation of tinnitus with (TH) or without (T) the co-occurrence of hyperacusis (Hofmeier et al., 2018, 2021). In these studies T was linked with delayed and reduced ABR wave V and reduced evoked BOLD fMRI responses in the medial geniculate body (MGB) and auditory cortex (AC) in response to higher frequency stimuli, while TH was characterized by a smaller reduction in ABR wave V and partial elevation in evoked BOLD fMRI responses in the AC in response to broader frequency stimuli (Hofmeier et al., 2021). In an independent tinnitus cohort using a multivariate model, Edvall et al. identified, hyperacusis as a strong confounder for ABR waves (Edvall et al., 2022). It can be concluded from these findings that the overall reduced and delayed auditory-specific responsiveness in the T-group may be best explained by previous assumptions that a loss of fast high spontaneous rate (high-SR) auditory nerve fiber (ANF) processing in tinnitus frequency channels leads to reemergence of spontaneous hyperexcitability caused by loss of tonic parvalbumin (PV) interneurons in deprived cortical regions (Knipper et al., 2020). This would lead to less memory-linked contrast amplification and more noise and, as a result, would promote further alertness and attention to the phantom noise (for review, see Knipper et al., 2020). With the co-occurrence of hyperacusis (TH), a more widespread signal amplification process appears to proceed through an overactive thalamocortical activity that may trigger an excitation spread to limbic/pain regions and results in inadequately high attention to increased loudness at all sound frequencies (Knipper et al., 2020), as was also previously hypothesized (Koops and van Dijk, 2020).

In order to search for functional biomarkers that can be used for the subclassification of tinnitus in everyday clinical practice and to confirm the hypothesis that brain responses may differ between T- and TH-groups, thus impeding the definition of relevant neural pathogenesis of T alone, we tested cortical electroencephalography (EEG) responses during active auditory discrimination, focusing on gamma oscillations in T- and TH-groups. While numerous studies analyzed slower brain oscillation changes in tinnitus and linked changes in the alpha, theta, and delta band to tinnitus (Weisz et al., 2007; Leske et al., 2014; Schwippel et al., 2019; Li Y. H. et al., 2022), the role of gamma oscillations in auditory cortex of tinnitus subjects remains unclear and controversial (van der Loo et al., 2009; Vanneste et al., 2010; De Ridder et al., 2015; Vanneste et al., 2018). This may be explained by the fact that EEG studies have not differentiated between T- and TH-patients in the past and have often only reported spontaneous EEG findings. In addition to analyzing spontaneous EEG from the same session, the present study reports results from EEG recorded during an attention-requiring two-tone auditory discrimination task to test whether central processing differences between groups might interfere with perceptual performance in response to acoustic stimulation within and outside the individual tinnitus frequency range. EEG was combined with simultaneous functional near-infrared spectroscopy (fNIRS), which provides hemodynamic activity, which we compared to the results of resting-state-fMRI-based functional connectivity (rs-fMRI-bfc) between auditory-specific networks. Previous studies using fNIRS in tinnitus subjects have demonstrated that during auditory stimulation, the auditory and parietal cortex respond differentially in tinnitus and control subjects and dissociate during rest (Issa et al., 2016; Zhai et al., 2021). The analysis of rs-fMRI-bfc, evoked and induced EEG oscillations, and hemodynamic responses revealed by fNIRS, all in similar brain regions, provided more profound insights into subgroup-specific differences. This approach also unraveled novel associations between the methods, with simultaneous EEG and fNIRS allowing for a direct comparison between neural oscillations and hemodynamic activity. Identifying changes in oxygenated and deoxygenated hemoglobin (oxy- and deoxy-Hb) concentrations during the same period has also been suggested to make small sample sizes acceptable (Mauri et al., 2020; Pinti et al., 2020; Wang et al., 2022).

Regarding our hypothesis of tinnitus being linked to a decline in high-SR ANF processing (see for a review Knipper et al., 2013, 2020, 2021) previous findings on phase-locked responses of auditory nerve fibers being mainly coded by high-SR ANF (Huet et al., 2019), constitute an important dimension for the discussion of the expected cortical EEG response changes. Thus, the precision of neural phase locking that predicts neural synchrony in central auditory system processing (Clinard et al., 2010; Walton, 2010; Anderson et al., 2012; Encina-Llamas et al., 2019; Marcher-Rorsted et al., 2022) and both, phase locking and oscillatory power in gamma bands have previously been shown to predict hemodynamic BOLD responses in human auditory cortex (Oya et al., 2018).

This combined cross-method approach allowed for identifying characteristic group differences between T- and TH-group, which likely will optimize future sub-classification of tinnitus subjects and thus improve treatment approaches.

## Materials and methods

This study was conducted in the Department of Otolaryngology (Patient Recruitment and Audiometry), the Department of Diagnostic and Interventional Neuroradiology (fMRI), and the Department of Psychiatry and Psychotherapy (EEG and fNIRS) at the University of Tübingen. The study was approved by the ethics committee of Tübingen University (Faculty of Medicine; ethical approval-number 264-2016BO1, 391/2018B02, 092/2020BO2 and follow-up study 383/2021BO2). All participants gave written informed consent. All methods were used according to the Declaration of Helsinki by the World Medical Association for human research ethics. The present study aims to validate objective markers previously identified for the differentiation of tinnitus with and without hyperacusis (Hofmeier et al., 2018, 2021; Refat et al., 2021) and to search for differential changes in brain oscillations and hemodynamic responses in an extended cohort. Employing previously described inclusion and exclusion criteria (Hofmeier et al., 2018, 2021) along with strict subclassification criteria for hyperacusis, the hyperacusis questionnaire (HKI) developed by Goebel and Berthold (Fischer, 2013), and the Loudness Discomfort Level Test (Goldstein and Shulman, 1996), a total of 86 subjects aged 19–57 years were included (Supplementary Tables 1, 2, Total cohort).

### Participants

From 86 subjects (for age, gender, and handedness, see Supplementary Tables 1, 2), 30 patients complained of tinnitus without co-occurrence of hyperacusis (T-group), and 17 complained of tinnitus with co-occurrence of hyperacusis (TH-group). The remaining 39 participants were included as controls (C-group). Hearing loss did not exceed 20 dB at each frequency from 0.125 to 3 kHz and 40 dB at each frequency from 4 to 10 kHz in the pure tone audiogram (PTA).

Age and sex were not evenly distributed among the groups (Supplementary Table 1), requiring tests for age and sex-based contributions in subsequent analyses. Not all of the total cohort were available for later measurements. Thus, pure tone audiometry up to 16 kHz could be measured only in a subgroup of 22 control subjects (C-group), 18 patients with tinnitus without the concomitant presence of hyperacusis (T-group), and 11 patients in whom tinnitus occurred with hyperacusis (TH-group; Supplementary Table 2, Gr1). In this study, a portion of the total cohort underwent rs-fMRI measurements (consisting of 12 C, 15 T, and 6 TH; Supplementary Table 2, Gr2). Another subgroup, which included 17 C, 16 T, and 8 TH, was tested with an active pitch discrimination task while recording EEG and concurrent fNIRS (Supplementary Table 2, Gr3). The majority of the individuals in the subgroups Gr1, Gr2, and Gr3 overlapped (Supplementary Figure 1; Supplementary Table 2).

### Audiological evaluation

Before the initial measurement session, all subjects provided written and informed consent, a physician conducted an ear examination, and an anamnesis was performed to determine the exclusion criteria. Because we are and were aware that a clean distinction of hyperacusis from other forms of reduced sound tolerance is at least challenging (Jastreboff and Jastreboff, 2023), special attention was paid to this issue during recruitment of the participants, asking them (1) first directly with an open question (“Stören Sie bestimmte Töne oder Geräusche?”) whether there were any particularly unpleasant sounds or noises for them, and (2) second, we specifically addressed examples like the sound of e. g. chewing, eating, smacking, clicking of a pen, rubbing styropore or chalk on a blackboard (“Stören Sie Geräusche wie Kauen, Schmatzen, Kugelschreiber klicken, Reiben von Styropor oder Kreide auf einer Tafel?”). If the answers were positive, we excluded the subjects from the study. Audiological measurements, including tympanometry, PTA (air and bone conduction), loudness discomfort levels (LDLs), tinnitus localisation (frequency and loudness), the Goebel-Hiller-Score [GHS, reliability (Cronbach’s alpha) α = 0.93 (Goebel and Hiller, 1994)] tinnitus questionnaire, and the hyperacusis questionnaire HKI (α = 0.93; Berthold-Scholz, 2013) were administered as described in Supplementary material and Hofmeier et al. (2018). In addition to the default PTA ranging from 0.25–10 kHz, the study also evaluated extended high frequency (EHF) PTA hearing thresholds at 11.2, 12.5, 14, and 16 kHz using the AT 1000 Audiometer (Auritec, Medizindiagnostische Systeme GmbH, Germany) with on-ear headphones (HDA 300, Sennheiser, Germany). Additionally, speech audiometry was performed using the German version of the matrix test, the “Oldenburger Satztest” (OLSA), with on-ear headphones (AT 1350 A, Beyerdynamic, Germany). OLSA assesses the speech reception threshold by repeating random German five-word sentences. It includes two phases: binaural speech signals without noise and monaural speech signals with contralateral noise at 65 dB SPL (Frank and Karlovich, 1975).

### Hyperacusis classification

To assess the grade of hyperacusis, LDLs were measured at 0.25, 0.5, 1, 2, 4, and 6 kHz (Supplementary Table 4), and the hyperacusis questionnaire (HKI; Supplementary Figure 2) was administered (Fischer, 2013) in all participants. The HKI and LDL methods classify patients into four hyperacusis severity quartiles (none, mild, moderate, severe) with an additional hyperacusis cut-off value at HKI score > 11 (Goldstein and Shulman, 1996; Berthold-Scholz, 2013). For the control group, we specified an HKI burden below the cut-off value and a none or mild burden measured by LDLs. For tinnitus patients in the TH-group, we considered a moderate to severe burden as measured by LDLs combined with a mild to severe HKI burden. Also included are subjects with a mild burden, as measured by the LDLs, and a moderate to severe HKI burden. The remaining tinnitus patients with a lower LDL burden than moderate were included in the T-group.

### ABR measurement

The ABR measurement was derived ipsilaterally with an amplifier [actiCHamp Plus and EP-PreAmp (x50), Brain Products, Germany] according to the manufacturer specifications with a sampling rate of 50 kHz. Measurements were performed with four electrodes (Neuroline 720, Ambu, Germany), with electrode impedance consistently below 2 kΩ [ground: Fpz—above the nasion; Reference—inverting input (−): Fz–hairline; Non-inverting input (+): mastoid]. The ABR was elicited with broadband acoustic click stimuli (83 μs) presented with 65 to 85 dB SPL in 10 dB steps and a repetition rate of 11.1 Hz. The stimuli had 3,000 repetitions of alternating polarity, with a sampling rate of 44.1 kHz. Acoustic stimulation was delivered via shielded in-ear transducers (ER-2, Etymotic Research Inc., United States) with disposable foam ear tips (ER1-14A, Etymotic Research Inc., United States), using the sound card (Scarlett 8i6 3rd Gen, Focusrite, United Kingdom). The absolute latencies and amplitudes of distinct ABR waveform components were extracted from the bandpass filtered (30–2,000 Hz; First order FIR filter, Hamming windowed), inverted, and averaged ABR of the single ears at each given stimulus level and attributed concerning stimulus onset. The most prominent positive peak, occurring typically at 5–6 ms after stimulus onset, was determined as wave V. Subsequently, wave I and III were determined in the range of 2, respectively, 4 ms before wave V. Wave amplitudes were assessed and predefined by measuring the peak-to-peak amplitude between the positive and trailing negative deflections. For wave latencies, the latencies of positive peaks were chosen.

### Pulsed distortion-product otoacoustic emissions

Within a subgroup of 18 participants (controls = 12, tinnitus = 6), we acquired three distortion-product otoacoustic emissions (DPOAE) metrics, i.e., amplitude, threshold (lowest measurable DPOAE), and estimated threshold (derived by extrapolation) derived from pulsed DPOAE (pDPOAE) level maps based on pDPOAE measurements. Moreover, to complement these results, we conducted behavioral threshold assessments using modified Békésy-Tracking audiometry in these individuals (Zelle et al., 2020; for further information, see Supplementary material).

### Functional magnetic resonance imaging

Functional magnetic resonance imaging (fMRI) image acquisition was performed on a 3-Tesla scanner (PrismaFit, Siemens Healthineer, Germany) with a 64-channel head–neck coil. The resting state functional images for the whole brain were acquired with a gradient echo planar imaging sequence over a 5 min acquisition period of awake rest. Exact measurement parameters and rs-fMRI analysis were performed as described in detail in the Supplementary material.

### EEG and fNIRS measurement setup

**Figure 1:**
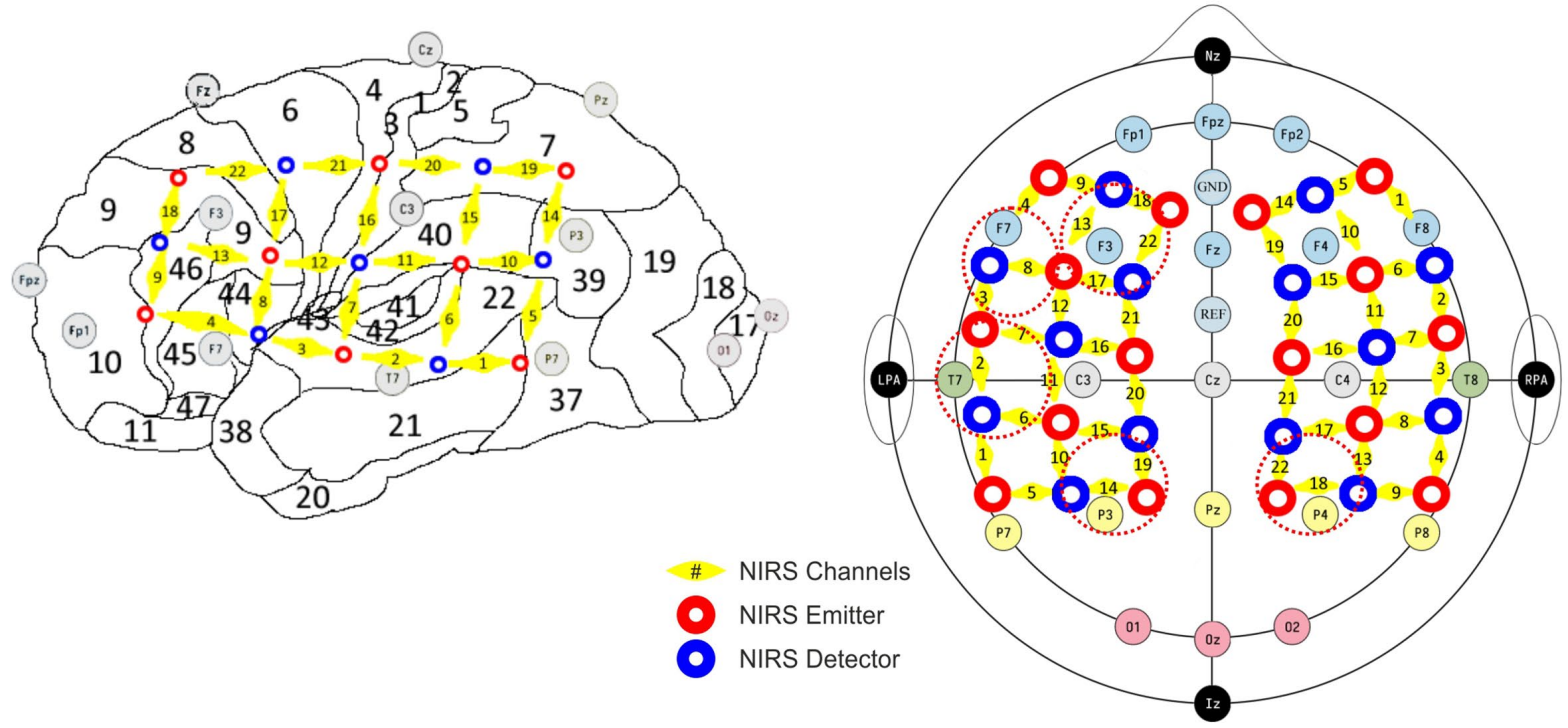
Sampling encompassed the auditory cortex (BA41, 42) channel configuration T7/T8, including superior temporal gyrus (BA22), corresponding to NIRS channels 2/6/7 left and 3/7/8 right; temporoparietal areas in channel configuration P3/P4 (Wernicke’s area, BA39, 40 and ventral part of BA7) covered by NIRS channels 14/19 left and 18/22 right; the ventrolateral prefrontal areas (BA44, 45, 46) in channel configuration F7/F8 covered by channels 4/8/9 left and 1/5/6 right; the dorsolateral prefrontal regions (BA9DL) covered by channels 13/17/18/22 left and 10/14/15/19 right (F3/F4).

Simultaneous EEG and fNIRS recordings were obtained with a specialized cap integrating both sensor types. EEG signals were recorded at a sampling rate of 1 kHz using 21 passive electrodes, positioned according to the international 10/20 system, with FCz as the reference and AFz as the ground electrode (Figure 1, gray disks; Jasper, 1958). To maintain optimal signal quality, electrode impedances were kept below 5 kΩ. The recording system consisted of a Vision Recorder data acquisition software receiving signals from a DC amplifier (BRAINAMP DC 32 channel module, Brain Products, Germany). The concentration changes of oxygenated and deoxygenated hemoglobin [(de)oxy-Hb] were measured by a continuous wave, multichannel NIRS system (ETG-4000 Optical Topography System, Hitachi Medical Co., Japan) with a temporal resolution of 10 Hz using two 22-channel optode arrays covering the left and right fronto-temporo-parietal head areas (12 × 6 cm each; inter-optode distance: 30 mm; Figure 1, red/blue disks).

### Two-tone discrimination task and resting state paradigm

Concurrent EEG and fNIRS were acquired while subjects performed a tone discrimination task or were at rest with eyes open. The participants were seated with the instruction to fixate at a cross in the center of the computer screen while being exposed to two different pure-tone frequencies with a duration of 250 ms each, a gap of 250 ms, and a sampling rate of 44.1 kHz. The participant’s task was to recognize which tone was higher/lower in pitch and respond by keystroke. The difficulty of this task was constantly adapted to each participant’s performance by changing the frequency difference such that the response given by the subject was correct in 3 out of 4 trials. All auditory stimuli were presented at 65 dB above individual hearing level (HL) via a Monitor Speaker (Model MSP5, Yamaha Corporation, Japan). The task comprised two distinct stimulus frequency bands: one centered at 1 kHz, referred to as fRef, and another centered at the individual’s tinnitus frequency, referred to as fTin [for controls 6 kHz based on the average tinnitus frequency in Hofmeier et al. (2018)]. A total of 6 blocks of 20 trials were acquired for each of the two center frequencies, resulting in a total of 240 stimulus pairs, followed by 8 min of awake rest with eyes open. EEG and fNIRS resting state were acquired over a 5 min acquisition period of awake rest.

### EEG data processing

Raw data were downsampled to 500 Hz (Anti-aliasing: cut-off = 400, transition bandwidth = 100) and detrended with NoiseTools to remove low-frequency drifts < 3 Hz (de Cheveigne and Arzounian, 2018). 2.5 s epochs were extracted for further processing, including a 0.5 s baseline in the tone discrimination task and a time offset of ±0.1 s in the resting state epochs. Open and flat channels were interpolated from surrounding channels, and epochs with artifacts, such as muscle artifacts or jumps, were removed based on variance and visual inspection. Subjects were excluded if more than 10% of channels or epochs needed removal. Then, an independent component analysis (ICA) was performed to identify and remove further artifacts embedded in the data (Delorme and Makeig, 2004). After removing clearly artifact-contaminated components, the EEG data were referenced to the average reference.

Finally, a time-frequency decomposition analysis was carried out on every epoch and for all electrodes, utilizing a resolution of 200 time and 199 frequency points [using EEGLAB newtimef(); complex Morlet wavelet transformation with a length of 1.2 cycles at the lowest frequency of 4 Hz and linear increase up to 29.85 cycles at the highest frequency of 199 Hz (Delorme and Makeig, 2004)]. The calculated power data were subjected to both conventional analyses, involving epoch averaging and comparison, as well as screening for single trial events, as described in Neymotin et al. (2022).

### NIRS data processing

Preprocessing encompassed interpolation of channels with missing data from surrounding channels (arithmetic mean), Temporal Derivative Distribution Repair (Fishburn et al., 2019) for artifact reduction and bandpass filtering (0.001–0.1 Hz). Trial blocks with interruptions of several seconds between trials were cut, and only the extended continuous portion was included in further analysis. Additionally, the thus-prepared signal was visually inspected, and trials with overt and dominant artifacts were manually removed. Maps representing Hb concentration changes were generated through linear interpolation of averaged group channels, with increased concentrations depicted in red/yellow and decreased in turquoise/blue. Sampling encompassed prefrontal areas, parietal areas, and last but not least, the superior temporal gyrus (Table 1; Figure 1, yellow).

**Table 1:** Regions of interest with their respective Brodmann areas, EEG electrodes, and fNIRS channel configurations.

Brain region	Brodmann area	EEG electrode	fNIRS channel right	fNIRS channel left
Auditory cortex	BA41, 42 and BA22	T7, T8	3, 7, 8	2, 6, 7
Temporoparietal areas	BA39, 40 and ventral BA7	P3, P4	18, 22	14, 19
Ventrolateral prefrontal areas	BA44, 45, 46	F7, F8	1, 5, 6	4, 8, 9
Dorsolateral prefrontal areas	Dorsolateral BA9	F3, F4	10, 14, 15, 19	13, 17, 18, 22

BA, Brodmann Area.

### Statistical analysis

Unless otherwise noted, statistical significance was tested at the level of α = 5%. The level of significance is illustrated in the figures with symbols or shaded areas [not significant (ns): *p > 0.05*; •: *p < 0.1*; *: *p < 0.05*; **: *p < 0.01*; ***: *p < 0.001*; ****: *p < 0.0001*]. All statistical tests were performed with MATLAB or GraphPad Prism v9.1.

## Results

### Elevated distress in tinnitus subjects with co-morbid hyperacusis

**Figure 2:**
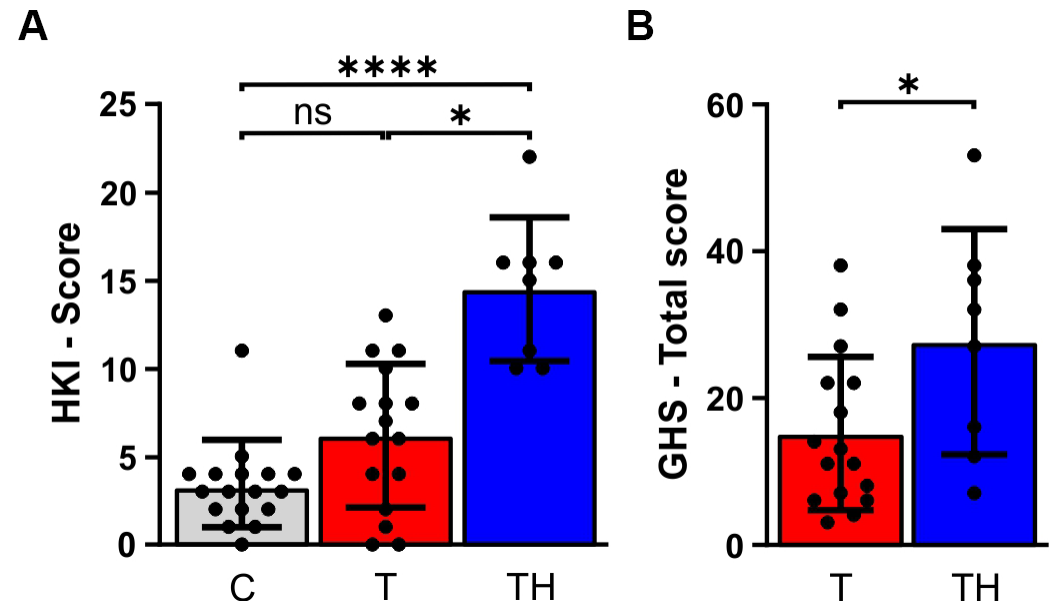
Results of the tinnitus and the hyperacusis questionnaire. The bar charts represent mean ± SD for **(A)** hyperacusis questionnaire scores, **(B)** GHS Total score between C- (gray), T- (red), and TH-group (blue). C, Control; T, Tinnitus; TH, Tinnitus & Hyperacusis; GHS, Goebel-Hiller-Score; HKI, Hyperakusis-Inventar; SD, standard deviation. *: *p* < 0.05; ****: *p* < 0.0001.

All included subjects, independent of being part of Gr1, Gr2, or Gr3, were classified using the HKI and LDL, measured as described in Supplementary material and previous studies (Hofmeier et al., 2021; Refat et al., 2021). Additionally, the Goebel-Hiller-Score (GHS) was used to assess tinnitus severity, emotional distress, cognitive distress, self-experienced intrusiveness, auditory perceptual difficulty, sleep disturbance, and somatic complaints. The subgroup Gr3 (EEG, fNIRS) revealed significant group differences in the HKI (Figure 2A) and the GHS total score (Figure 2B), the former demonstrated by a Kruskal-Wallis test in the HKI Score [H(2) = 19.13, *p* < 0.0001]. Dunn’s multiple comparison tests indicated significant differences between C- (mean rank = 13.7, *n* = 17) and TH-group (mean rank = 36.06, *n* = 8, *p* < 0.0001), as well as between T- (mean rank = 21.22, *n* = 16) and TH-group (mean rank = 36.06, *n* = 8, *p* < 0.01; Figure 2A). To test significance of differences in GHS total score, a Mann–Whitney U test was performed (U = 31, *n*_1_ = 16 *n*_2_ = 8, *p* = 0.042, Figure 2B).

Notably, the GHS sub-score differences in the subgroup Gr3 with 41 subjects (Supplementary Figures 3K–P) were not different from that in the total cohort with 86 subjects (Supplementary Figures 3C–H), both groups expressing significant group differences in HKI and GHS total score among C-, T-, and TH-groups. Higher tinnitus severity was observed in the TH- compared to the T-group, regardless of whether the total score or sub-scores were considered (Supplementary Figures 3A–P), with significance being reached in the total score, as previously reported (Schecklmann et al., 2014; Hofmeier et al., 2021; Refat et al., 2021). Taken together, irrespective of the small selected cohort sizes used in the present study, the distress caused by tinnitus was reproducibly greater in tinnitus subjects with co-morbid hyperacusis.

### No T- and TH-specific differences in hearing threshold up to 16 kHz

**Figure 3:**
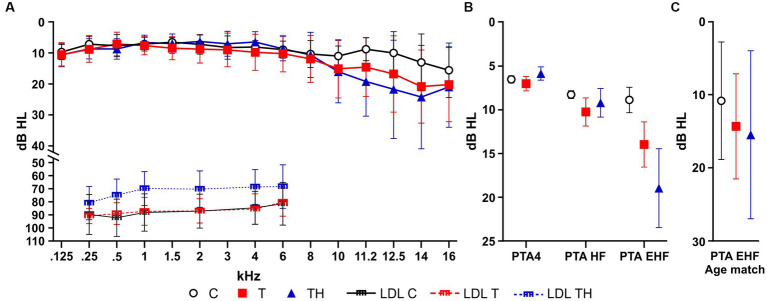
Hearing sensitivity thresholds for left and right ear were determined by pure tone audiometry (PTA) plotted as mean ± SD for C- (*n* = 22, black), T- (*n* = 18, red), and TH-group (*n* = 11, blue). **(A)** PTA thresholds in dB HL for each measured frequency. **(B)** PTA4, PTA-HF, and PTA-EHF thresholds plotted as mean ± SD for C- (*n* = 22, black), T- (*n* = 18, red), and TH-group (*n* = 11, blue). **(C)** PTA-EHF thresholds plotted as mean ± SD for an age-matched subgroup C- (*n* = 5, black), T- (*n* = 5, red), and TH-group (*n* = 5, blue). dB, decibel; HL, hearing level; PTA, pure tone audiometry threshold; PTA-EHF, PTAs in extended high frequencies (better ear of 11.2–16 kHz); PTA-HF, PTAs in medium frequencies (better ear of 6–10 kHz); PTA4, PTAs in low frequencies (better ear of 0.5, 1, 2, 4 kHz); SD, standard deviation; LDL, loudness discomfort level.

As described in the methods section, PTAs were collected for frequencies between 0.125 and 16 kHz in the subgroup Gr1. We confirmed here again that the TH-group, unlike C- or T-group, showed a decreased LDL over the frequency range of 0.25–6 kHz (Figure 3A). The PTA4, which considers PTA thresholds at 0.5, 1, 2, and 4 kHz (von Gablenz and Holube, 2015), PTA-HF, which accounts for thresholds at 6, 8, and 10 kHz, and PTA-EHF, including thresholds within the extended high-frequency regions (EHF) covering 11.2, 12.5, 14, and 16 kHz, were plotted for C-, T-, and TH-group (Figure 3B). Because of inhomogeneous group variances, a non-parametric mixed-effect analysis had to be performed, indicating a statistical trend [F(2, 48) = 2.77, *p* = 0.0727], while the Holm-Sidak multiple comparison tests revealed no significant group differences. Although apparent, the threshold increases in T and even stronger TH were evident in the EHFs (Figure 3B). However, the descriptive differences between C-, T-, or TH-groups in the EHF region do not reach significance when we normalize the groups for age (Figure 3C). To verify that threshold differences did not contribute and differed between the C- and T/TH-group, we confirmed in an independent cohort of 12 controls and six patients with tinnitus using pDPOAE and Békésy-Tracking audiometry that the observed threshold variations can be explained by the cochlear amplifier (see Supplementary material; Supplementary Figure 4).

### Tinnitus may influence the threshold around tinnitus frequency, tinnitus loudness, and distress through tinnitus loudness differentially in T- and TH-subjects

We next investigated whether individual PTA thresholds, specifically within the tinnitus frequency range, were related to tinnitus loudness. This information is fundamentally relevant for the subsequently required ability of subjects to correctly classify frequency differences in our active auditory tone discrimination task, during which simultaneous EEG and fNIRS activities were recorded in response to acoustic stimulation within and outside the tinnitus frequency range.

**Figure 4:**
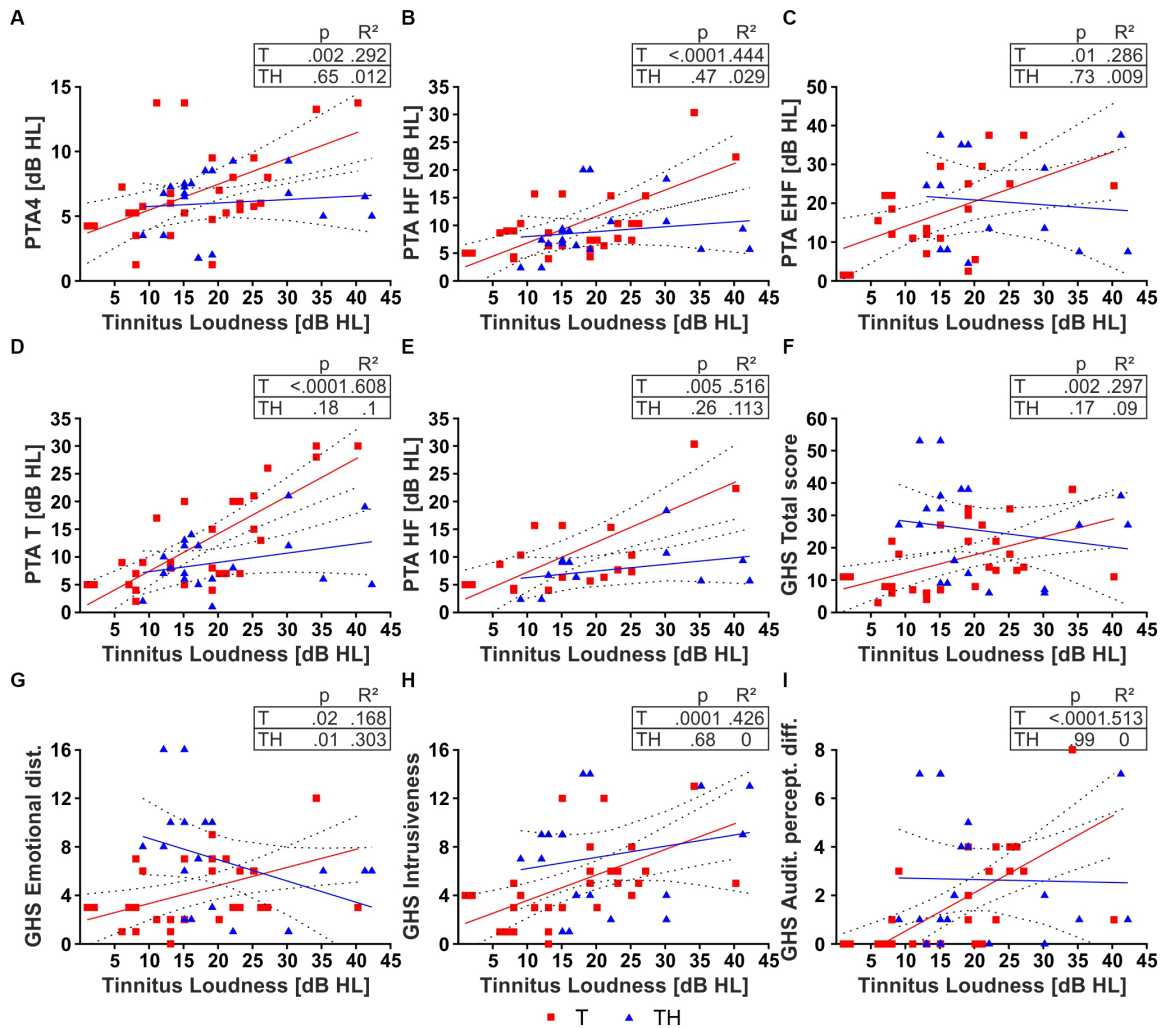
Two-tailed Pearson correlation for T- (red) and TH-group (blue) **(A–E)**. Tinnitus loudness and **(A)** PTA4, **(B)** PTA-HF, **(C)** PTA-EHF, **(D)** PTA-T, **(E)** PTA-HF in subjects with individual tinnitus in the range of 6–10 kHz. Two-tailed Spearman correlation for T- (red) and TH-group (blue) **(F–I)**. Tinnitus loudness and **(F)** GHS Total score, **(G)** GHS Emotional distress, **(H)** GHS Intrusiveness, **(I)** GHS Auditory perceptual difficulties. dB, decibel; GHS, Goebel-Hiller-Score; HL, hearing level; PTA, pure tone audiometry threshold; PTA-EHF, PTAs in extended high frequencies (better ear of 11.2–16 kHz); PTA-HF, PTAs in medium frequencies (better ear of 6–10 kHz); PTA-T, PTAs in individual tinnitus frequency; PTA4, PTAs in low frequencies (better ear of 0.5, 1, 2, 4 kHz).

We observed significant positive correlations between individual tinnitus loudness and PTA-HF (Figure 4B), and PTA thresholds in the individual tinnitus frequency (PTA-T), the latter approximating the PTA threshold of the tinnitus frequency (Figure 4D) in T-, but not in the TH-group. Moreover, weak correlations were observed for PTA4 and PTA-EHF (Figures 4A,C) in the T-, but not TH-group.

A significant increase in correlation becomes detectable in the T-group for PTA-HF also when we only consider tinnitus subjects with a tinnitus frequency in the PTA-HF spectrum (Figure 4E), suggesting a dominant role of threshold elevations within the tinnitus frequency range to tinnitus loudness. We next hypothesized that tinnitus loudness in the TH-group did not correlate with the PTA-T threshold, as the distress in TH-subjects is already higher at low tinnitus loudness. Actually, when tinnitus loudness was correlated with the different GHS scores, not only the total score (Figure 4F), but furthermore the sub-scores, as defined by emotional distress (Figure 4G), intrusiveness (Figure 4H), and auditory perceptual difficulties (Figure 4I), all parameters were positively correlated with tinnitus loudness in the T-group. In contrast, most parameters were uncorrelated in the TH-group, except emotional distress, which was negatively correlated. Also, no correlations were found for sleep disturbance and cognitive distress with loudness in the T-group and a significant negative correlation in the TH-group (Supplementary Figures 5A,C). The correlations with tinnitus loudness (Supplementary Figures 5D–O) were additionally presented in dB SL, i.e., corrected with the PTA threshold. Due to this correction, we observe a loss of correlation of tested tinnitus loudness to hearing thresholds (Supplementary Figures 5G–K). However, we observed tinnitus loudness SL still correlates with GHS for T-, but not for TH-group (Supplementary Figures 5L,N).

**Figure 5:**
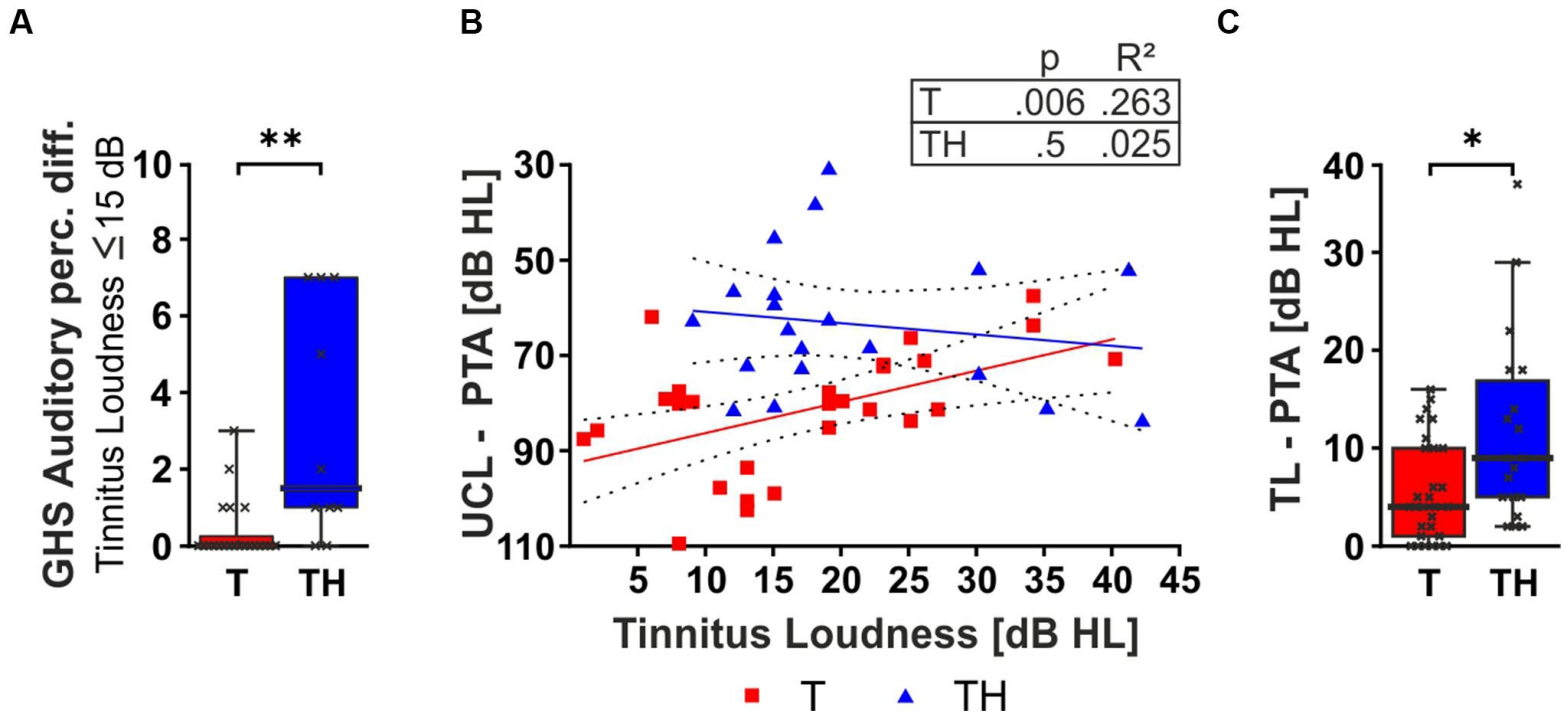
The box plot **(A)** shows the median, range (whiskers), and quartiles (box) of the GHS Auditory perceptual difficulty (Auditory Perc. Diff.) score for patients with self-rated tinnitus loudness intensity ≤ 15 dB HL. **(B)** Two-tailed Spearman correlation between tinnitus loudness and the dynamic range (PTA-LDL). The box plot **(C)** shows the median, range (whiskers), and quartiles (box) of the tinnitus loudness corrected for PTA threshold for T- (red) and TH-group (blue). dB, decibel; GHS, Goebel-Hiller-Score; HL, hearing level; PTA, pure tone audiometry threshold; LDL, loudness discomfort level. *: *p* < 0.05; **: *p* < 0.01.

Hypothesizing that the difference in the correlation of GHS sub-scores to tinnitus loudness between T and TH was solely due to inherent differences in perceived loudness between groups, we next considered GHS auditory perceptual difficulty scores for both groups only for subjects with low tinnitus loudness levels ≤ 15 dB HL (Figures 4I, 5A). Indeed, the perceived burden of auditory perception difficulty with comparably low tinnitus loudness is already significantly higher in the TH-group (U = 38, *p* = 0.0016), meaning that maximum distress is reached in the TH-group through low tinnitus loudness levels. In accord with tinnitus loudness and distress scoring only in T-, but not in TH-groups, the LDL threshold correlated positively with the individual tinnitus loudness perception in the T-, but not the TH-group (Figure 5B). To test independently of the distribution of scores, we applied a Mann–Whitney U test which revealed a significantly greater PTA threshold-corrected tinnitus loudness in the TH-group (U = 162, *p* = 0.0157; Figure 5C).

In conclusion, this shows that the TH- differs from the T-group, particularly in how stressed subjects were due to their brains’ responses to the individual tinnitus loudness, visible through a strong correlation of PTA-T thresholds and GHS distress scores.

### Suprathreshold ABR amplitude and latency were affected differentially in T- and TH-subjects

Motivated to validate T- and TH-specific characteristics, as described above, we next explicitly addressed the question of differential central auditory response behavior of the three groups, searching for aspects of central auditory differences in the ABR amplitude.

**Figure 6:**
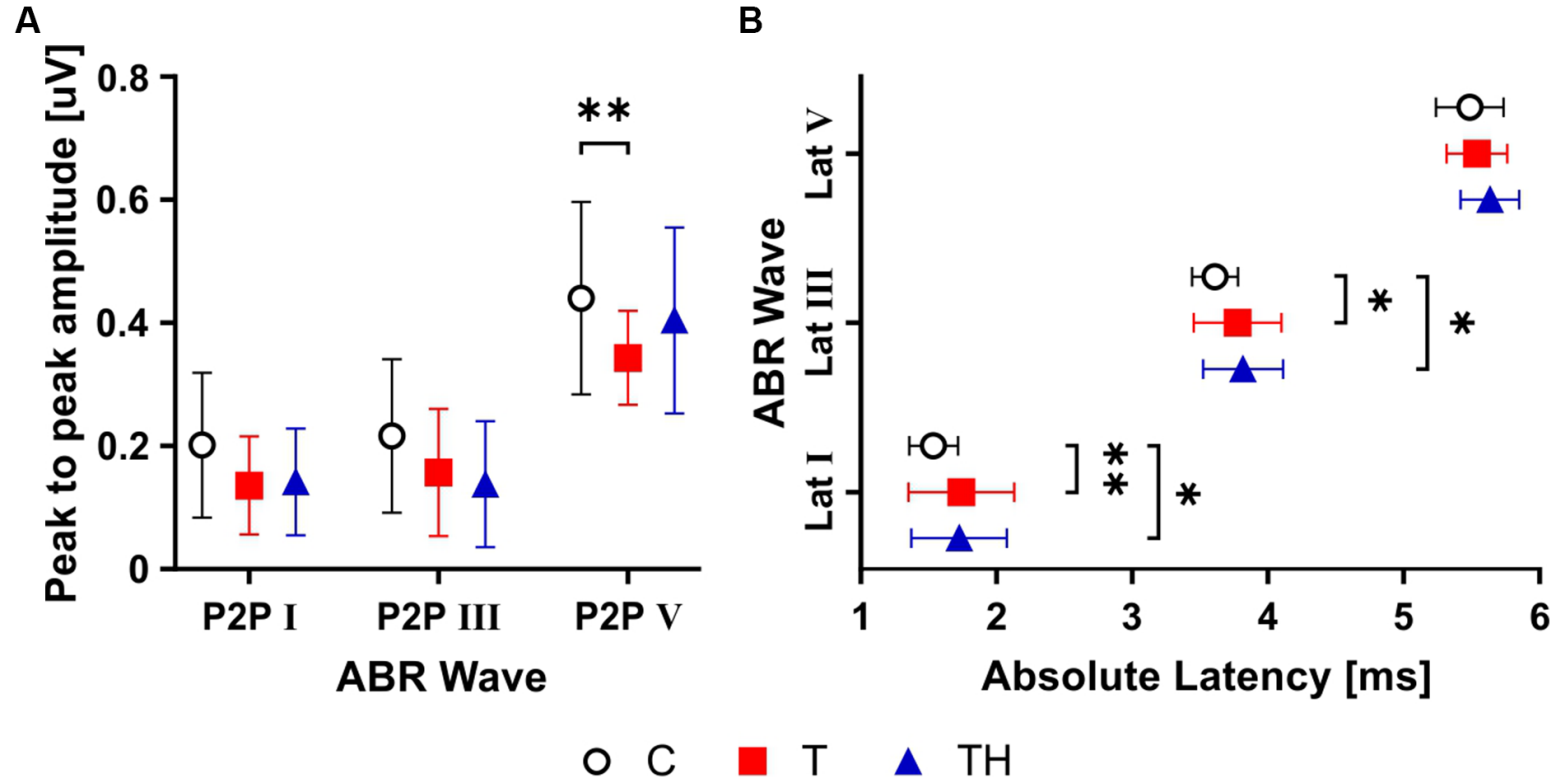
Graphs **(A,B)** show mean ± SD for ABR wave amplitude and latency group differences. Averaged ABR wave I, III, and V peak-to-peak (P2P) amplitudes **(A)**, absolute latency **(B)**. ABR, auditory brainstem response; Lat, latency. *: *p* < 0.05; **: *p* < 0.01.

As we recently demonstrated that tinnitus patients express reduced and delayed ABR wave responses (Hofmeier et al., 2018), we were particularly interested in reproducing the same effects in a smaller sample. Subsequently, we searched for differences in ABR wave I [generated in the auditory nerve (Portmann et al., 1980)], ABR wave III [generated in the superior olivary complex (SOC) and lateral lemniscus (Melcher and Kiang, 1996)], and ABR wave V [generated in the inferior colliculus (IC) output or the medial geniculate body (MGB; Møller et al., 1994; Figures 6A,B)].

A two-way ANOVA analysis revealed a significant difference for ABR wave amplitudes, measured at 85 dB SPL stimulus level, among all three groups (F (2,227) = 10.4, *p* < 0.0001). Consistent with previous findings (Hofmeier et al., 2018; Refat et al., 2021), subsequent Holm-Sidak’s multiple comparison tests demonstrated significant group differences between the C- (mean = 0.44 ± 0.156, n_Ears_ = 36) and the T- (mean = 0.34 ± 0.07, n_Ears_ = 28) groups for wave V amplitude (*p* = 0.003; Figure 6A).

The T- and TH-groups showed prolonged peak latencies of ABR waves I, III, and V compared to the C-group, with a significant group difference revealed by a two-way ANOVA (F (2, 228) = 10.34, *p* < 0.0001; Figure 6B). Holm-Sidak’s multiple comparison tests demonstrated significant group differences between C- (n_Ears_ = 35) and T-groups (n_Ears_ = 27, *p* < 0.01) and between C- and TH-groups (n_Ears_ = 16, *p* = 0.04) for wave I latencies, as well as between C- (n_Ears_ = 36) and T-groups (n_Ears_ = 27, *p* = 0.03) and between C- and TH-groups (n_Ears_ = 16, p = 0.03) for wave III latencies. The latency shift of ABR waves I and V was not positively correlated with or linked to age differences of the T-group, but was weakly correlated with age for the TH-group (Supplementary Figure 6A). We could not confirm ABR wave V or III latency shortening as in the previously observed TH-group (Hofmeier et al., 2021).

Thus, the current findings confirm a differential amplitude reduction of ABR wave V and an elongation of the latencies of ABR waves I and III in the T-, but less so in the TH-group. These differences indicate that neural activity (discharge rate or number of active neurons) or their synchronicity which all can determine ABR wave peaks (Johnson and Kiang, 1976; Kujawa and Liberman, 2009) are possible functional biomarkers, particularly for the T-group. However, this may not be the most reliable criterion for differentiating T and TH for such small groups.

### No speech discrimination threshold differences between C-, T-, and TH-groups

As other groups described effects on language comprehension in tinnitus patients and we previously observed fMRI BOLD activity pattern differences in language processing regions of the cortex (Hofmeier et al., 2018), we also performed the German matrix sentence test “Oldenburger Satztest” (OLSA) as described (Wagener et al., 1999; HörTechgGmbH, 2012). To test whether measured audiometric parameters (Figures 4–6) may influence other aspects of auditory processing, e.g., speech understanding, we analyzed the speech reception threshold in quiet and under fixed noise conditions. The speech reception threshold of the OLSA was determined with headphones at a fixed noise level of 65 dB SPL using a standard adaptive procedure, converging at 50% speech intelligibility. No differences in OLSA thresholds could be observed between the groups, neither in quiet (Supplementary Figure 6B) nor upon noise conditions, as shown for the left and right ear (Supplementary Figure 6C). However, a higher variability is recognizable, especially for the right acoustically exposed speech stimuli in T- and TH-groups that may need further consideration.

In conclusion, Tinnitus with or without co-occurrence of hyperacusis does not show any speech reception threshold differences when using OLSA, although higher variability in OLSA thresholds in T- and TH-groups may need future consideration.

### Differentially reduced rs-fMRI-based functional connectivity in auditory and temporal, parietal, and prefrontal cortex of T- and TH-subjects

Sound-evoked ABR wave amplitude reflects, for instance, synchronized neural activity (Johnson and Kiang, 1976; Ruttiger et al., 2017). Stronger signal coordination or more synchronous resting state BOLD fluctuations in task-relevant areas of neocortex were shown to predict behavioral performance (Haag et al., 2015). We, therefore, hypothesized differences in rs-fMRI connectivity between the groups, specifically a differential weakening of synchronous auditory neural activity as reflected in lowered and delayed ABR waves (Figure 6). For the subgroup Gr2, the subjects of which were nearly all included in EEG and fNIRS recordings (Supplementary Table 2), we compiled the frequency of significant connections among predefined ROIs. We then analyzed the frequency of significant correlations with priority for those cortical ROIs, which roughly corresponded to our ROIs applied in the analyses of EEG and NIRS signals.

**Figure 7:**
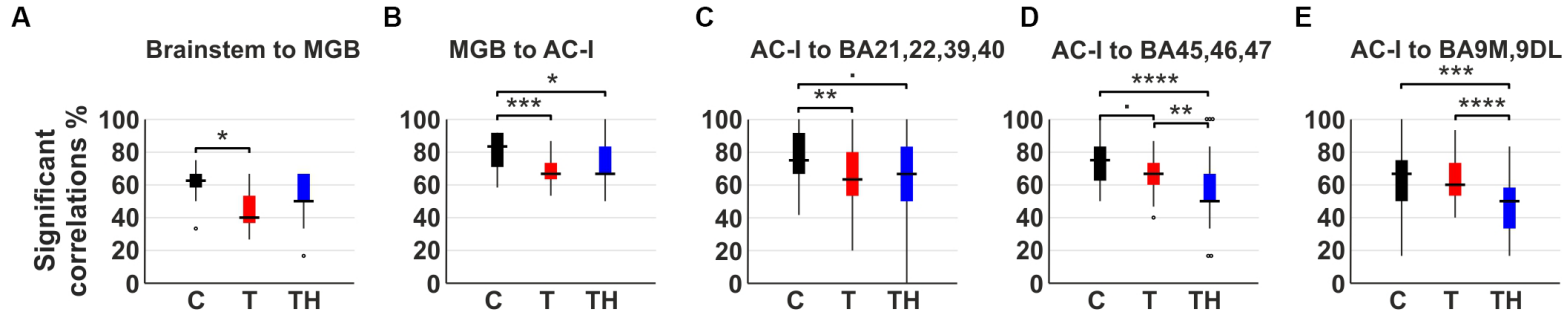
The boxplots display the frequency of significant positive rs-MRI BOLD correlations between predefined ROIs in **(A)** subcortical regions and MGB, **(B)** MGB and AC-I, **(C)** AC-I and temporoparietal cortex (BA21, 22, 39, 40), **(D)** AC-I and ventrolateral prefrontal cortex (BA45, 46, 47), **(E)** AC-I and anterior mesial and dorsolateral prefrontal cortex (BA9M, 9DL) for C- (*n* = 12, black), T- (*n* = 15, red), and TH-group (*n* = 6, blue). Regarding the management of the family-wise error rate, the *p*-values obtained from the Kruskal-Wallis test were adjusted by Bonferroni correction. AC-I, primary auditory cortex; BA, Brodmann area; BOLD, blood oxygenation level depended; DL, dorsolateral; M, medial; MGB, medial geniculate body; ROI, region of interest. •: *p* < 0.1; *: *p* < 0.05; **: *p* < 0.01; ***: *p* < 0.001; ****: *p* < 0.0001.

**Figure 8:**
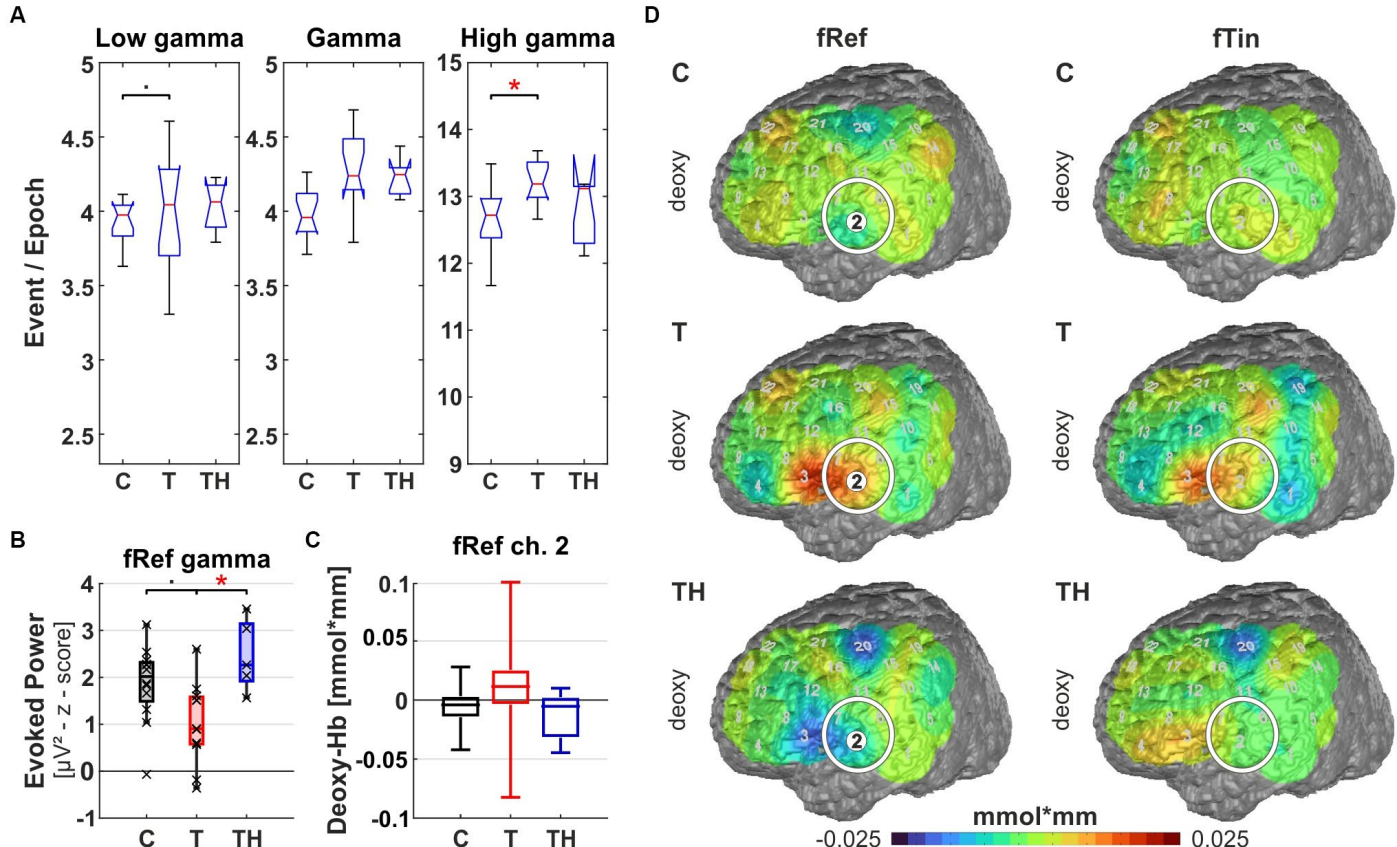
Results from region of interest “T7” for oscillations and hemodynamic activity: Box plots display median, quartiles, and range of measures derived from signals recorded at the T7 electrode for C- (*n* = 12), T- (*n* = 15), and TH-group (*n* = 6). **(A)** Spontaneous oscillation events per epoch at different gamma frequency ranges. The left panel represents the low gamma [21–40 Hz], the center panel represents (mid) gamma [41–60 Hz], and the right panel represents the high gamma [61–120 Hz] band. **(B)** Evoked gamma [41–60 Hz] power, left and right plots comparing responses to stimulation with fRef and fTin. **(C)** fNIRS deoxy-Hb response to fRef stimulation in the left channel 2: Box plots display median, quartiles, and 5–95 percentile. **(D)** fNIRS deoxy-Hb changes in response to fRef (left column) and fTin (right column). The white circle highlights the region of interest on the pseudocolor maps, which represent simple group averages of (here only) deoxy-Hb concentration in mmol*mm, in subsequent figures, we combine oxy- and deoxy-Hb plots with identical scaling. See Supplementary Figure 12 for all stimulation frequencies and NIRS signals. •: *p* < 0.1; *: *p* < 0.05.

**Figure 9:**
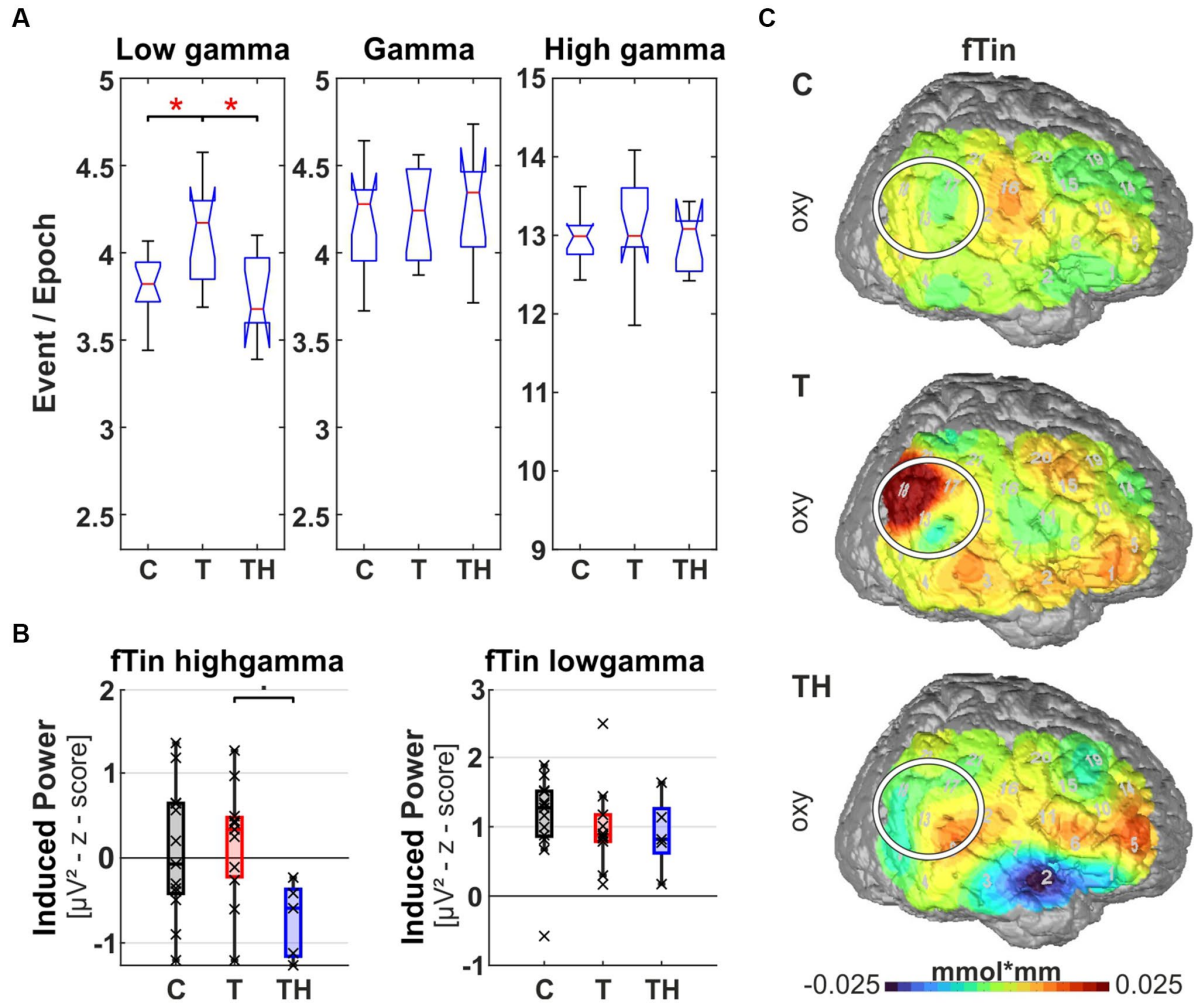
Results from region of interest “P4” for oscillations and hemodynamic activity: Box plots display median, quartiles, and range of measures derived from signals recorded at the P4 electrode for C- (*n* = 12), T- (*n* = 15), and TH-group (*n* = 6). **(A)** Spontaneous oscillation events per epoch at different gamma frequency ranges. The left panel represents the low gamma [21–40 Hz], the center panel represents (mid) gamma [41–60 Hz], and the right panel represents the high gamma [61–120 Hz] band. **(B)** Induced gamma power in response to fTin stimulation, left and right plots comparing high [61–120 Hz] and low [41–60 Hz] gamma. **(C)** fNIRS oxy-Hb changes in response to fTin. •: *p* < 0.1; *: *p* < 0.05.

**Figure 10:**
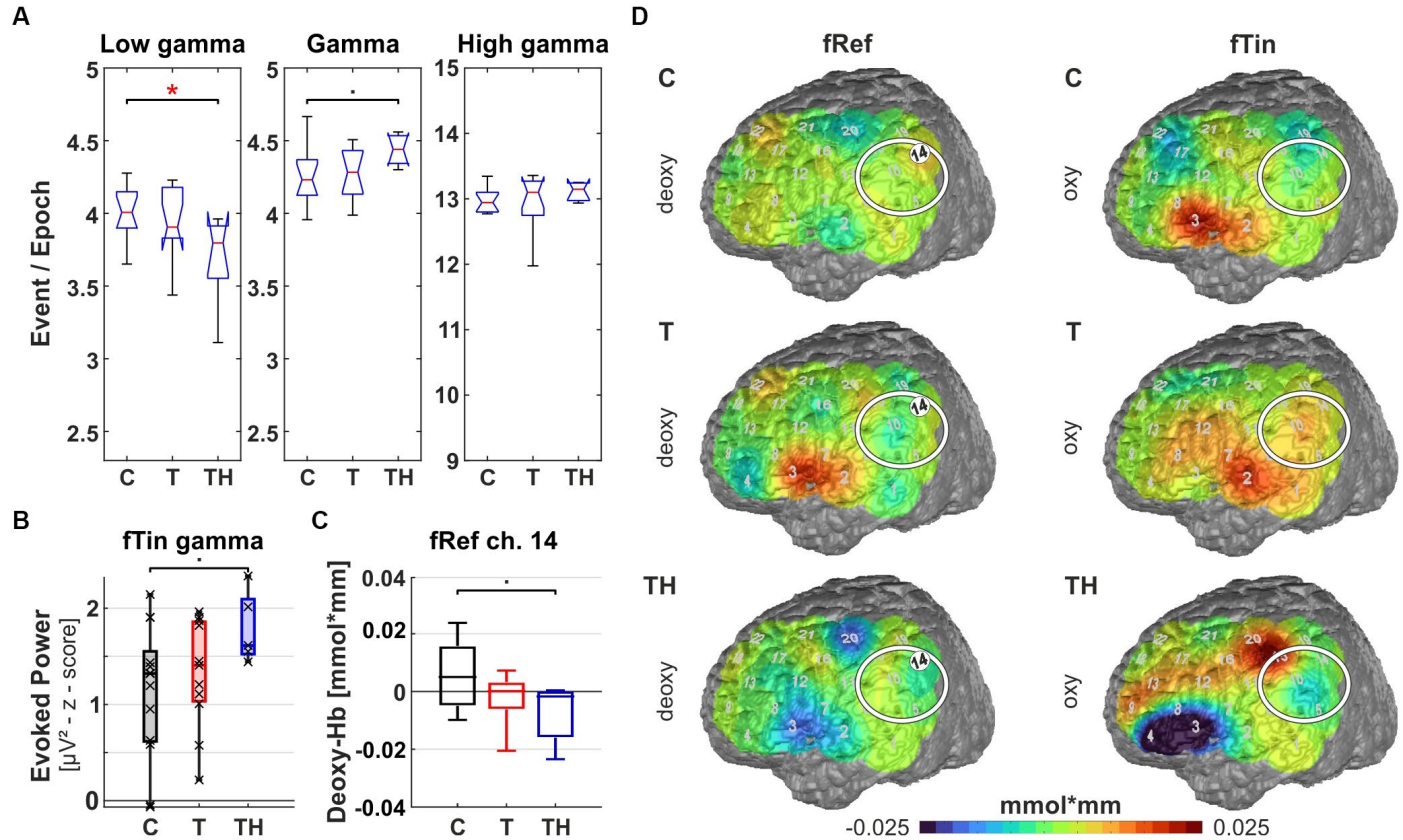
Results from region of interest “P3” for oscillations and hemodynamic activity: Box plots display median, quartiles, and range of measures derived from signals recorded at the P3 electrode for C- (*n* = 12), T- (*n* = 15), and TH-group (*n* = 6). **(A)** Spontaneous oscillation events per epoch at different gamma frequency ranges. The left panel represents the low gamma [21–40 Hz], the center panel represents (mid) gamma [41–60 Hz], and the right panel represents the high gamma [61–120 Hz] band. **(B)** Oscillation responses to fTin stimulation with left and right plots comparing responses of evoked and induced gamma [41–60 Hz]. **(C)** fNIRS deoxy-Hb response to fRef stimulation in the left channel 14: Box plots display median, quartiles, 5th and 95th percentiles. **(D)** fNIRS deoxy-Hb changes in response to fRef and oxy-Hb changes in response to fTin. •: *p* < 0.1; *: *p* < 0.05.

**Figure 11:**
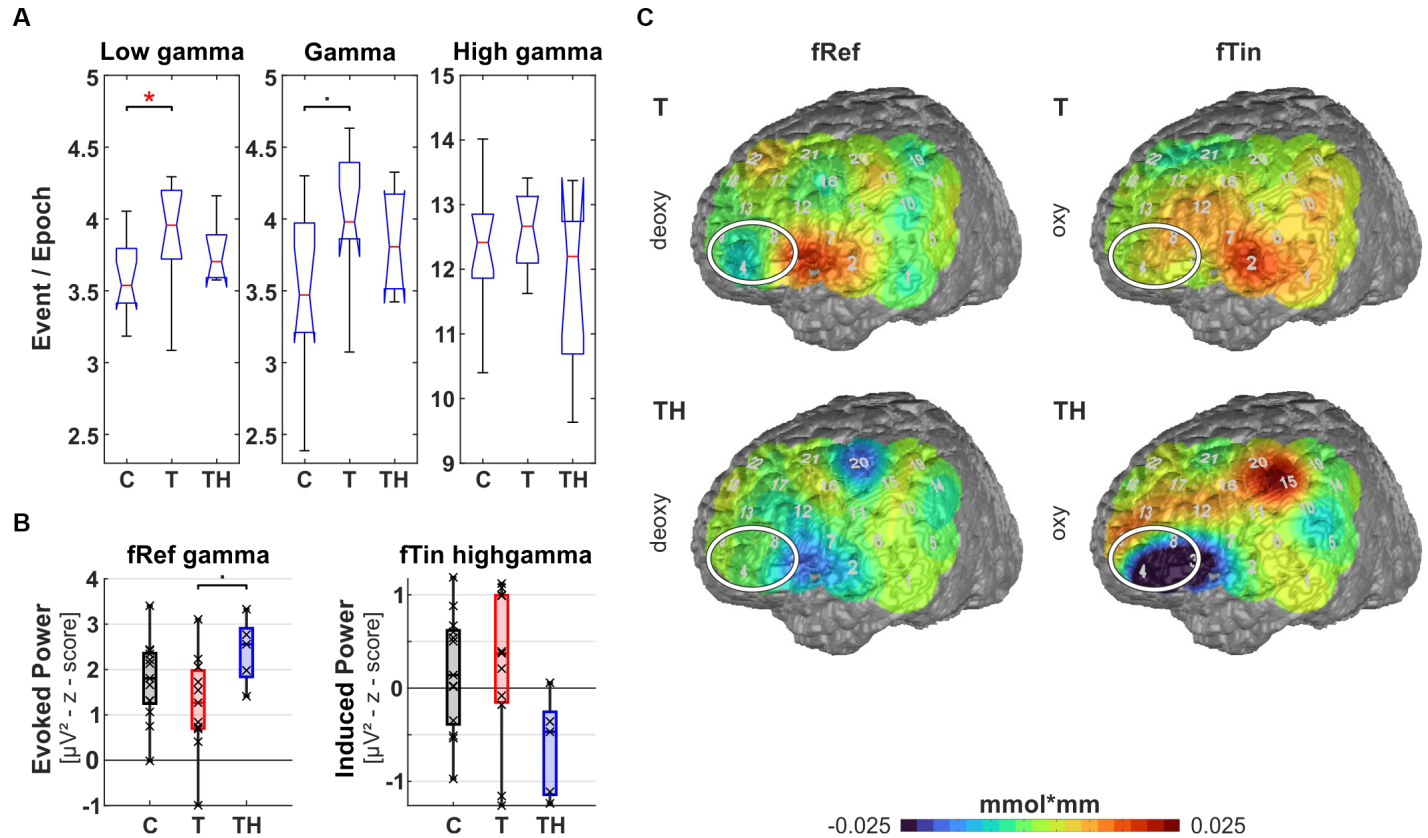
Results from region of interest “F7” for oscillations and hemodynamic activity: Box plots display median, quartiles, and range of measures derived from signals recorded at the F7 electrode for C- (*n* = 12), T- (*n* = 15), and TH-group (*n* = 6). **(A)** Spontaneous oscillation events per epoch at different gamma frequency ranges. The left panel represents the low gamma [21–40 Hz], the center panel represents (mid) gamma [41–60 Hz], and the right panel represents the high gamma [61–120 Hz] band. **(B)** Evoked gamma [41–60 Hz] power, display responses to stimulation with fRef (left), and induced high gamma [61–120 Hz] power, display responses to stimulation with fTin (right). **(C)** fNIRS oxy-and deoxy-Hb changes in response to fRef (left column) and fTin (right column). •: *p* < 0.1; *: *p* < 0.05.

**Figure 12:**
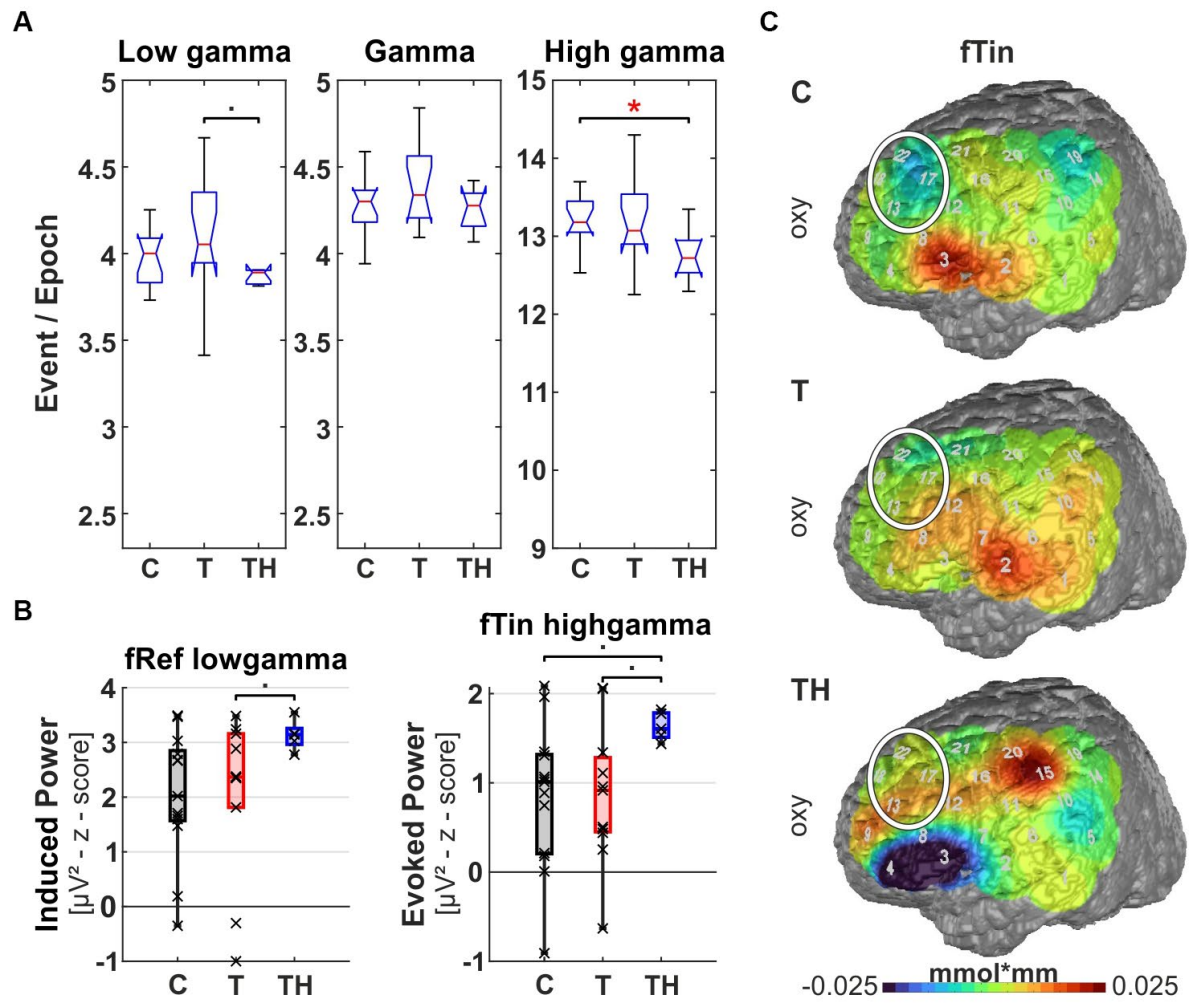
Results from region of interest “F3” for oscillations and hemodynamic activity: Box plots display median, quartiles, and range of measures derived from signals recorded at the F3 electrode for C- (*n* = 12), T- (*n* = 15), and TH-group (*n* = 6). **(A)** Spontaneous oscillation events per epoch at different gamma frequency ranges. The left panel represents the low gamma [21–40 Hz], the center panel represents (mid) gamma [41–60 Hz], and the right panel represents the high gamma [61–120 Hz] band. **(B)** Induced low gamma [21–40 Hz] power displayed responses to stimulation with fRef (left) and evoked high gamma [61–120 Hz] power, showing responses to stimulation with fTin (right). **(C)** fNIRS oxy-Hb changes in response to fTin. •: *p* < 0.1; *: *p* < 0.05.

The presence of positive spontaneous correlations at rest, caused by neuronal events (Florin et al., 2015), is considered to have a physiological basis (Zhang et al., 2020) despite their complex dependence on neuronal and vascular parameters. Since we are interested in biomarkers that can be assessed in individual subjects, we examined whether the regions are significantly correlated for individual subjects and perform a group comparison based on the individual level. If the BOLD response of two brain regions in an individual subject correlated significantly, these regions were considered functionally connected, although we cannot determine the directionality of interaction, i.e., which region influences the other. Herein, we regarded the suggestion that a lack of correlation provides compelling insights into impaired or altered information transmission or changes in default network structure (Husain and Schmidt, 2014; Chen Y. C. et al., 2017; Hullfish et al., 2018; Rosemann and Rauschecker, 2023), which are interpreted according to the respective regions. Frequencies of positively correlated connections were depicted for each subject within the C-, T-, and TH-groups in (i) brainstem regions as the CN, SOC, IC to the MGB (Figure 7A; Supplementary Figure 7A); (ii) the MGB to the AC-I regions (BA41, 42; Figure 7B; Supplementary Figure 7B), which approximate the EEG/fNIRS channel configuration of T7/T8 (Figures 1, 8); (iii) the AC-I to middle temporal (BA21, 22) and the temporoparietal junction or Wernicke area (BA39, 40), involved in sound detection and evaluation, i.e., semantic language processing (Yantis et al., 2002; Schwartz et al., 2012; Ardila et al., 2016; Coslett and Schwartz, 2018; Figure 7C; Supplementary Figure 7C), approximating the EEG/fNIRS channel configurations of P3/P4 (Figures 1, 9, 10); (iv) the AC-I to regions of the attention/stress-regulating networks: part of the ventrolateral prefrontal network, including inferior frontal gyrus (BA45 and BA47; Husain, 2016; Leaver et al., 2016a; Chen Y. C. et al., 2017) and part of the dorsolateral prefrontal cortex (BA46; Cieslik et al., 2015; Figure 7D; Supplementary Figure 7D), brain regions approximating the EEG/fNIRS channel configuration of F7/F8 (Figures 1, 11); (v) the AC-I to the dorsolateral and medial BA9 (BA9DL, 9 M) prefrontal cortex regions involved in distress regulation (Lai et al., 2019; Li L. et al., 2022; Figure 7E; Supplementary Figure 7E), regions approximating the EEG/fNIRS channel configuration of F3/F4 (Figures 1, 12).

The boxplots show the difference in frequency of significant positive correlations between C-, T-, and TH-groups (Figure 7). Overall, we observed a general trend for reduced frequencies of positive correlations in T compared to C, less pronounced for TH in “auditory-specific” connections. Dunn’s multiple comparison tests confirm this significant reduction of the T-group in brainstem regions to the MGB (*p* = 0.015; Figure 7A; Supplementary Figure 7A), MGB to AC-I (*p* = 0.0009; Figure 7B; Supplementary Figure 7B), and AC-I to Wernicke (*p* = 0.0016, Figure 7C; Supplementary Figure 7C). In contrast, the frequency of connections from AC-I to the ventrolateral prefrontal networks (Figure 7D; Supplementary Figure 7D) or distress-related dorsolateral prefrontal networks (Figure 7E; Supplementary Figure 7E) exhibited only a minor decrease in connectivity frequencies within the T-group in comparison to the C-group. However, through Dunn’s multiple comparison tests, a significantly more pronounced decline in connection frequencies was observed in the TH-group, specifically from AC-I to BA45, 46, 47 (C-TH *p* < 0.0001, T-TH *p* = 0.0014; Figure 7D), as well as from AC-I to BA9M, 9DL (C-TH *p* = 0.0002, T-TH *p* < 0.0001; Figure 7E). When the number of frequencies between the left AC-I and medial prefrontal cortex (BA9M) and dorsolateral prefrontal cortex (BA9DL) was considered separately, it became evident that the correlations to BA9DL for T and TH decreased in comparison to C, while those to BA9M increased evidently in the TH-, but not in the T-group (Supplementary Figures 7F,G). This could prove to be an important new observation considering the group differences in distress levels in response to tinnitus loudness and the differential role attributed to BA9M and BA9DL in stress regulation (Sullivan and Gratton, 1999, 2002; Wang et al., 2005; Kern et al., 2008).

Taken together, the analysis of the frequency of significant positive correlations between distinct ROIs in the ascending auditory pathway up to the AC and associated higher cortical regions, including the extended Wernicke region, provided evidence for a significant decline in network correlations, particularly for the T-group. However, a stronger and differential reduction of connectivity to the dorsolateral and ventrolateral prefrontal cortex regions became evident for the TH-group (Figures 7D,E).

### Simultaneous EEG and fNIRS recordings identified differential gamma oscillations at rest linked with partially reversed evoked gamma and altered hemodynamic responses in T- and TH-groups

To evaluate brain oscillations during active auditory perception, comparing T- and TH-groups in comparison to control subjects, we employed a two-tone (pitch) discrimination task. Subjects were exposed to stimulus pairs in two different frequency ranges: (i) around their tinnitus frequency in T/TH or 6 kHz in control subjects, and (ii) around 1 kHz reference frequency. EEG served to analyze cortical oscillations, primarily focused on the gamma frequency range and distinguishing ongoing, evoked, and induced oscillations, while fNIRS served to determine hemodynamic changes in temporal, parietal, and frontal brain regions. As depicted in Figure 1 and Table 1, sampling encompassed the auditory cortex, temporoparietal areas, ventrolateral prefrontal areas, and dorsolateral prefrontal areas. fNIRS provides concentrations of oxy-Hb and deoxy-Hb in mmol*mm, which we here interpret in the context of metabolic brain activity following neural activation similar to fMRI BOLD activity. As there is no simple relation among these different signal modalities (Steinbrink et al., 2006; Pinti et al., 2021), we only use changes in hemodynamic activity across space to determine activity differences across regions and groups. Thereby, we interpret an increase in the deoxy-Hb signal as reduced brain activity and, conversely, a decrease as increased brain activity. For oxy-Hb signals, the interpretation is reversed—an increase in the oxy-Hb signal is interpreted as increased brain activity and a decrease as reduced brain activity.

In a first approach, a single trial analysis of resting state EEG was performed for distinct channels, known to sample primarily from particular brain regions, which revealed that ongoing oscillation patterns in different gamma frequency bands differed for C-, T-, and TH-groups (Figures 8–12A). Next, the evoked and induced oscillatory responses averaged across all 240 stimulus pairs were computed separately for responses to reference (1st and 3rd rows) and test/tinnitus frequencies (2nd and 4th rows). Power differences were observed in some of the three gamma frequency bands analyzed here (Supplementary Figures 8–11). For the fNIRS signals, we provide average group maps (Supplementary Figure 12) for the three groups, as the sample size is limited: control subjects in the left, T-subjects in the middle, and TH in the right column. The upper row contains both oxy- (upper plots for left and right hemisphere) and deoxy-Hb maps (respective lower plots for left and right hemisphere) for reference stimuli (fRef) and the lower row for tinnitus/test stimuli (fTin).

Focusing the analysis of EEG and concurrent fNIRS recordings on specific brain regions, our highest priority were those regions, among which altered rs-fMRI-bfc between the subject groups had been detected (Figure 7). We first noticed that for all EEG and fNIRS channel configurations, significant distinctions in spontaneous gamma band activity during rest, as well as evoked or induced gamma band power, were predominantly observed in the left hemisphere for the C-, T-, and TH-groups (Figures 8, 10–12). However, one surprising exception revealed a power increase of low-frequency gamma oscillations at electrode P4, corresponding to the right parieto-temporal region, including the right hemisphere homolog of Wernicke’s areas BA39, 40 and the ventral part of BA7 (Figure 9). Correspondingly, the data sets for resting state, evoked or induced gamma power of C-, T-, and TH-groups of the right hemisphere, which did not reveal any significant group differences, are presented in Supplementary Figures 13–15. In the more ventral brain regions comprising temporal and inferior frontal cortex, we observed increased resting gamma power, which was associated with decreased evoked gamma activity, while in more dorsal brain regions, including dorsolateral prefrontal and temporoparietal areas of the left hemisphere, decreases in spontaneous gamma power were associated with increases of evoked gamma power.

Left temporal region (including EEG electrode T7, NIRS channels 1, 2, 3, 6, 7 over LH, “configuration T7”): Here, we observe signals from the auditory cortex (BA41, 42, and BA21, 22 STG/MTG; NIRS channels 2, 6, 7) during resting state and active auditory perception when subjects pressed buttons in response to discriminating different pitch frequencies. At rest, we observed a significant increase of high gamma power [61–120 Hz] (*p* = 0.018) in the T-group in comparison to the C-group as well as a trend of elevated low gamma power [21–40 Hz] (*p* = 0.08) in the T-, but not in the TH-group. According to *post hoc* analysis, the TH-group expressed the same median as T (Figure 8A). Along with increased ongoing high gamma, we observed reduced evoked gamma power [41–60 Hz] for stimuli at the reference frequency in T-, compared to the TH-group (*p* = 0.021), but only a trend (*p* = 0.055) between C- and T-group (Figure 8B). In analogy to the predicted relation of synchronous activity and oscillatory power in (high) gamma bands predicting BOLD responses in AC (Oya et al., 2018), we observed associations of hemodynamic responses and oscillations in the T-group. In the tinnitus subjects, NIRS deoxy-Hb responses (warm colors) were increased in the left temporal region (Kruskal-Wallis test: *p* = 0.06, effect size d = 0.75, Figure 8C) and adjacent inferofrontal cortex BA44 (Chang et al., 2015). These hemodynamic responses occurred in response to stimuli at reference and less to tinnitus frequencies (Figure 8D, compare upper and middle rows). In contrast, in the TH-group, the temporal cortex expressed a compatible decrease of deoxy-Hb (cold colors) in response to the reference frequencies, not to the tinnitus frequencies. Thus, these findings show for the first time increased spontaneous but reduced evoked gamma oscillations [as predicted in Knipper et al. (2020)] in combination with increased variance in deoxy-Hb responses, indicating highly variable but decreased metabolic activation in and above the auditory cortex. From this, we conclude that the pathologically high activation level at rest leads to diminished synchronous auditory signaling during active auditory tasks at non-tinnitus frequencies in the T-group, masked in the TH-group, as elaborated in more detail in the discussion.

The right parieto-temporal junction (includes EEG electrode P4 and NIRS channels 13, 17, 18, 22 above RH, “P4 configuration”), corresponds to BA39, 40 in the right hemisphere, which is the homolog of the left-hemispheric Wernicke area and the more ventral parts of the posterior parietal cortex BA7. Here, we observed a highly significant elevation of spontaneous oscillatory events in the low gamma band [21–40 Hz] with the largest effect size of Cohen’s d = 1.258 in the T- (Kruskal-Wallis test: *p* = 0.006; *post hoc*: *p* = 0.029) compared to the C- and the TH-group (*post hoc*: *p* = 0.029, Figure 9A). Evoked gamma did not differ among groups, not even with a trend, but induced high gamma [61–120 Hz] showed a tendency to differ (*p* = 0.065) between C- and TH-groups (Figure 9B). In stark contrast to normal subjects, oxy-Hb activation in tinnitus subjects (middle plot in Figure 9C) showed a prominent peak in the right temporo-parietal cortex corresponding to BA39, 40 and possibly the ventral end of BA7 evoked by both reference and test stimulus frequencies in the T-group, but certainly not in the TH-group (Figure 9C, see also Supplementary Figure 12).

Thus, right parietal cortex expressed significantly stronger spontaneous low gamma oscillations in tinnitus subjects combined with strong oxy-Hb responses to auditory stimuli at both reference and test frequencies, suggesting that tinnitus subjects suffer from dysbalanced attentional mechanisms.

The left parieto-temporal junction “Wernicke’s area” (includes EEG electrode P3 and NIRS channels 10, 14, 15, 19 over LH, “P3 configuration”) corresponds to BA39, 40. Group differences in oscillation patterns revealed concordant spontaneous and task-evoked/induced oscillation changes. While at the right parieto-temporal junction, the hemodynamic oxy-Hb response was increased in the T- and not at all changed in the TH- compared to the C-group in response to fTin, the left parieto-temporal junction also followed that pattern, though at a much shallower level (Figure 10D, left). If anything, the increase of oxy-Hb in response to fTin is in contrast with the weak trend toward decreased deoxy-Hb responses revealed for fRef in the TH-group (Kruskal-Wallis test: *p* = 0.046, effect size d = 0.833; *post hoc*: *p* = 0.069; Figures 10C,D left). The corresponding differences in oscillatory power at P3 occurred during spontaneous activity in the low gamma band [21–40 Hz] (*p* = 0.017) with a decrease at an effect size of d = 0.975 (Figure 10A). Furthermore, we also observed a trend for elevated spontaneous activity in the mid gamma band [41–60 Hz] (*p* = 0.07) in the TH-group compared to controls (Figure 10A, center). This was linked with a trend for elevated evoked activity (*p* = 0.071) and reduced induced gamma activity [41–60 Hz] (*p* = 0.081), again exclusively in the TH-group in comparison to the C-group in response to stimulation in the tinnitus frequency range (Figure 10B).

The left ventrolateral prefrontal region (including EEG electrode F7, NIRS channels 4, 8, 9 over LH, “configuration F7”) provides signals from BA44, 45, and the ventral part of BA46, regions involved in executive language functions and object working memory. Within this region, we observed elevated spontaneous low gamma [21–40 Hz] (*p* = 0.04, d = 0.798) and a trend for increased spontaneous gamma [41–60 Hz] (*p* = 0.08) in the T- compared to the C-group but not in the TH-group (Figure 11A). This corresponds qualitatively to the changes described for the right temporoparietal junction. Again, associated with this increased ongoing gamma in tinnitus subjects, we observed during the two-tone discrimination task a trend for reduced evoked gamma [41–60 Hz] (*p* = 0.06) in T compared to TH at the reference frequency (Figure 11B), while at the tinnitus frequency, we saw a weak trend for high induced gamma [61–120 Hz] to be lower in TH compared to T (*p* = 0.099; Figure 11B, right). Here, distinct reversed fNIRS responses were observed when comparing the T- and TH-groups (Figure 11C): while in control subjects, we did not see any relevant modulations of hemodynamic signals on both sides in inferofrontal cortex (see Supplementary Figure 12), the T-group expressed a right hemispheric oxy-Hb response to fTin, in contrast to a strong reduction of oxy-Hb in the TH-group in the right hemispheric anterior convexity at BA47/45 (Supplementary Figure 12 for oxy, blue patches). Future studies will have to clarify whether the trend for reduced induced high gamma [61–120 Hz] (*p* = 0.099) in TH- compared to the T-group (Figure 11B, lower) is reflected by the observed decrease (blue) of oxy-Hb and whether deoxy-Hb level would reveal any information for the TH-group (see also Supplementary Figure 12 for deoxy).

The left dorsolateral prefrontal region (including EEG electrode F3 and NIRS channels 13, 17, 18, 22 over LH, “F3 configuration”) provides signals from the dorsolateral part of BA9 (BA9DL) and the dorsolateral part of BA46, suggesting that attentional control and working memory are reflected. Here, spontaneous high gamma power [61–120 Hz] was significantly lowered in TH compared to C (*p* = 0.02, Figure 12A, rightmost plot), while a trend of weaker low gamma power [21–40 Hz] separated TH from T (*p* = 0.077, Figure 12A, left). Interestingly, here the lowered spontaneous high gamma power in the TH-group was associated with increased evoked high gamma power [61–120 Hz] in the TH-group at the tinnitus frequency (Figure 12B, right), although only with a trend for TH distinct from C- (*p* = 0.075) and T-subjects (*p* = 0.094). Although not significant, induced low gamma power [21–40 Hz] occurring during discriminating tones at the reference frequencies shows larger variance in controls compared to T and TH (*p* = 0.09), the latter with a much higher mean. Once more, a reversed relation of spontaneous and evoked gamma activity differences could be observed for the T- and TH-groups. Hemodynamic activity differed more in response to stimulation at tinnitus frequencies in the oxy-Hb signal among all three groups showing reduced oxy-Hb in control subjects, while T- and TH-subjects showed an increased oxy-Hb response (Figure 12C).

We here observed consistently reduced fast auditory processing in T-subjects through reduced ABR wave V, reduced rs-fMRI-bfc between the auditory brainstem and MGB, as well as between MGB and AC in T-subjects, which correlated with enhanced spontaneous, reduced evoked gamma oscillations, and reduced deoxy-Hb activity of the left hemispheric temporal cortex in response to fRef stimulation (Graphical abstract; Figures 6–8). In contrast, the TH-group showed strong effects in response to tinnitus frequencies in left hemispheric ventrolateral prefrontal cortex (F7) and similar changes of spontaneous and evoked gamma oscillations in left dorsolateral prefrontal cortex (F3), while the associated hemodynamic responses were shallow both in ventro- and dorsolateral prefrontal cortex (see also Graphical abstract and compare Figures 11, 12).

In summary, spontaneous EEG analysis revealed increased low or high gamma power at T7, P4, and F7 in the T-group, while in the TH-group, a decrease of low or high gamma power was observed at P3 and F3. In contrast, during our active tone discrimination paradigm, oscillations evoked by the reference frequencies around 1 kHz expressed less power in the mid gamma range at T7 and F7 in the T-group, whereas, in the TH-group, the tinnitus frequency evoked gamma power was increased at P3 and F3. However, one exception in the TH-group was the region around F7, where mid gamma power evoked by reference frequencies was increased. The concurrent measurement of hemodynamic activity with NIRS revealed a reduction in fRef-evoked deoxy-Hb responses in the left temporal region (T7) while both the T- and TH-group exhibited increased fRef-evoked deoxy-Hb activity in Wernicke’s area (P3). These findings will be discussed in the context of the function of the respective cortical areas and attention networks.

## Discussion

In the present study, we describe for a comparatively small cohort of tinnitus subjects without (T) and with hyperacusis (TH): (i) differential distress caused by the tinnitus loudness, (ii) differentially reduced and delayed central auditory response behavior at the level of ABR waves and (iii) differentially reduced thalamocortical resting connectivity based on fMRI BOLD activity. In these thereby extensively validated T- and TH-subgroups, we observed (iv) differential patterns of spontaneous and task-dependent (evoked/induced) brain oscillations in response to an attention-requiring two-tone discrimination task, during which we recorded concurrent EEG and NIRS, the latter providing (v) differential hemodynamic activity maps. During the discrimination task, we observed altered spontaneous and evoked gamma responses, altered deoxy-Hb fNIRS responses in the auditory cortex of T, and increased deoxy-Hb activity in TH-subjects in response to stimulation at reference frequencies, which we discuss in the context of reduced synchronous response behavior as a specific characteristic of tinnitus (Graphical abstract). In contrast, the TH-group expressed differentially altered spontaneous and evoked brain oscillations in the gamma band associated with altered hemodynamic responses in the temporoparietal junction and prefrontal cortex explicitly in response to stimulation at the individual tinnitus frequencies (Graphical abstract). In our opinion, the differential central response behavior in T- and TH-subjects at their tinnitus frequencies is a finding that unravels the advantage of our two-tone discrimination task as a method of subclassification, as discussed in the following. These findings are discussed in the context of T and TH to differ in their distress response to tinnitus loudness that differentially influences attention and working memory of the individuals performing the discrimination task.

### Periphery: hair cells and synaptic vulnerability in T and TH

The present study relies on the precise classification of hyperacusis in individuals within the tinnitus cohort using LDL and questionnaires (Goldstein and Shulman, 1996; Berthold-Scholz, 2013). Unreached LDL thresholds, particularly in higher frequencies, are a well-known clinical limitation (Edvall et al., 2022), motivating the classification of hyperacusis in tinnitus patients using low-frequency LDL thresholds combined with the HKI questionnaire. Utilizing this method, in the present study, we could not detect any evidence of a hearing threshold reduction up to 8 kHz in T- and TH-groups compared to control subjects in agreement with our previous studies (Hofmeier et al., 2018, 2021; Refat et al., 2021). As a new finding, we add that no PTA hearing threshold differences at extended high frequencies up to 16 kHz were observed between C-, T- and TH-groups. In previous studies that mainly analyzed hearing thresholds up to 8 kHz, also no difference in hearing threshold or electromechanical properties could be observed when comparing tinnitus to control groups (Geven et al., 2011; Boyen et al., 2014; Gilles et al., 2016; Guest et al., 2017) or hyperacusis (Gu et al., 2010; Hebert et al., 2013; Schecklmann et al., 2014). This led to the general conclusion that neither tinnitus nor hyperacusis may be causally linked to the loss of outer hair cells (OHC). Previously, however, an elevation of thresholds within EHF regions has been considered in tinnitus (Shim et al., 2009; Kim et al., 2011; Peng et al., 2021; Song et al., 2021; Sendesen et al., 2022). Thus, based on a systematic review and meta-analysis including EHF between 9 and 20 kHz, some degree of cochlear mechanical dysfunction was suggested for tinnitus patients, which may not be detectable by conventional audiometry alone (Gilles et al., 2016; Dewey and Dhar, 2017; Jafari et al., 2022). Our findings in a small cohort so far cannot convincingly support this finding, neither when thresholds were measured using PTA up to 16 kHz (Figure 3), nor upon Békésy-Tracking audiometry up to 12 kHz (Supplementary Figure 4A), nor upon more accurate fine-structured analysis of OHC function (Zelle et al., 2013) using pDPOAE recordings (Supplementary Figures 4B,C). In future studies, a more detailed measurement of the fine-structured DPOAE/octave function is required.

The question of whether OHC function is involved in the development of tinnitus with and without hyperacusis and whether this happens with presumptive elevation of thresholds EHF or not is quite relevant. We observed an increase in ABR wave latency in tinnitus subjects (Hofmeier et al., 2018, 2021; Refat et al., 2021) that was further confirmed (Edvall et al., 2022). The postulated reduced ABR wave V and delayed ABR wave I in tinnitus subjects (Figure 6) is best explained with the involvement of high-SR low-threshold auditory fibers that might contribute to threshold differences even independent of altered OHC function, as previously suggested (Knipper et al., 2013; Rüttiger et al., 2013; Hofmeier et al., 2018; Knipper et al., 2020). Thus, the reduced and delayed ABR wave I points to a reduction in or deafferentation of fibers with a high spontaneous rate, low response thresholds (high-SR ANF), and short response latencies (Liberman, 1978). Different from low-SR high threshold ANFs that do not contribute to compound action potential thresholds of auditory fibers, the high-SR ANF elevate compound action potential threshold (Bourien et al., 2014) and could contribute through increases in population-discharge synchrony to low perceptual thresholds (Meddis, 2006; Heil et al., 2008). High-SR ANFs, less low-SR ANFs, moreover contribute to phase-locked responses for auditory nerve fibers (Huet et al., 2019) and are preferentially responsible for coding of envelope following responses (EFR) including contributions to high stimulus levels by off-frequencies [corresponding to EHF (Hunter et al., 2020)]. The precision of neural phase locking, in turn, that relies on joint activity across the population of auditory fibers, has been shown to define frequency-following responses (FFR) that reflect neural synchrony arising in the central auditory system (Clinard et al., 2010; Walton, 2010; Anderson et al., 2012). Importantly, EFR changes (Encina-Llamas et al., 2019) are suggested to influence FFR at lower carrier frequencies (Marcher-Rorsted et al., 2022) and are predicted as a potentially sensitive marker for cochlear synaptopathy. These FFR responses were not influenced by OHC dysfunction (Marcher-Rorsted et al., 2022). Elevated EHF thresholds, together with delayed and reduced ABR wave I/V might reflect diminished (high-SR) auditory fiber processing in the affected frequency range and thereby could potentially contribute to diminished FFR and neural phase locking. As previously predicted for auditory response, the FFR influenced hemodynamic BOLD responses through changed phase-locking and oscillatory power in gamma/high gamma bands (Oya et al., 2018). The link between altered neuronal synchrony within the tinnitus frequency range and altered perception in tinnitus may be found in the potential need for high-SR processing for maintaining the strength of tonic inhibition in ascending circuits (Knipper et al., 2020, 2021). In case this concept would be valid for tinnitus, reduced strength of tonic inhibition following diminished high-SR processing could, through corrupted noise cancelation (Rauschecker et al., 2010, 2015; Knipper et al., 2020) or nonlinear increase in variance (Zeng, 2020), explain the tinnitus percept (Knipper et al., 2020, 2021). Alternatively, the TH phenotype may result from multiplicative central gain (Zeng, 2020), possibly through the parallel involvement of type II fibers projecting from OHCs to the brain (Knudson et al., 2014; Sturm and Weisz, 2015; Refat et al., 2021) that, through increased attention to and increased salience of the forthcoming stimuli (Han et al., 2018; Shin et al., 2022), contribute to the observed lowering of loudness tolerance in TH-subjects. Differential contributions of distinct auditory fiber processing deficits in T and TH would also explain various inconsistencies in the change of the suprathreshold ABR waves in tinnitus subjects, which were described to be either unchanged (Shim et al., 2017; Turner et al., 2022), increased (Sendesen et al., 2022), or decreased (Milloy et al., 2017) in tinnitus cohorts that did not distinguish T- and TH-subgroups.

The group differences between T and TH in gamma power measured by EEG as well as hemodynamic responses (measured by rs-fMRI and fNIRS), can be explained rationally via such modified bottom-up processing between groups, as will be discussed below.

### Relation of hearing thresholds at tinnitus frequency to tinnitus loudness and distress

We observed that the TH-group differed from the T-group by GHS evidenced distress to low tinnitus loudness levels (Figure 5A), while in the T-group, distress scored particularly with tinnitus loudness and the threshold elevation within or around the tinnitus frequency (Figure 4D). Indeed, the correlation between tinnitus loudness and PTA threshold shift was determined by the tinnitus pitch, as validated here for PTA-HF (Figure 4E). This observation may be related to previous observations that state an impairment in the EHF regions in tinnitus patients to be involved in determining the hearing-level loudness of tinnitus (Song et al., 2021), a finding we could not test due to the small number of subjects with a tinnitus pitch >10 kHz. The significant difference in distress scores between our T- and TH-groups, as well as the differential relation of the tinnitus-loudness and distress scores, equals findings described for significantly larger cohort groups (Tyler and Conrad-Armes, 1983; Schecklmann et al., 2014, 2015; Ralli et al., 2017; Han et al., 2018; Aazh et al., 2019; Hofmeier et al., 2021; Koops et al., 2021; Refat et al., 2021; Shin et al., 2022), which strengthens the validity of the tinnitus subclassifications performed in the present study.

In conclusion, group differences in distress scores and tinnitus loudness emphasize that correlations of PTA-T threshold and tinnitus loudness may be valid functional biomarkers for the sub-entity classification of T- and TH-groups.

### Functional connectivity and neural processes in left temporal cortex

PTA-T threshold elevation in T- and TH-subjects were differentially related to tinnitus loudness and distress. Still, beyond clinical and psychosomatic parameters, elevated PTA-T thresholds were associated with differential changes in rs-fMRI-based functional connectivity between auditory brainstem, MGB, primary auditory cortex (BA41, 42), and even beyond, involving cortical regions supporting attention, memory, and distress regulation. These differences in functional connectivity were partly more pronounced for the T-group than the TH-group (temporal and parietal areas) and vice versa frontal areas (see Figure 7; Supplementary Figure 7). This finding is in line with previous studies in tinnitus patients, which observed a reduced and delayed ABR wave V to be linked to reduced sound-evoked BOLD fMRI activity in the auditory cortex (Hofmeier et al., 2018; Koops and van Dijk, 2020), and to reduced functional connectivity observed during sound-evoked activity (Boyen et al., 2014; Lanting et al., 2014), as well as to reduced rs-fMRI-bfc between auditory-specific brain regions and fronto-striatal regions (Leaver et al., 2016b; Hofmeier et al., 2018). In contrast, differential and partly enlarged subcortical and cortical activity was linked to hyperacusis in tinnitus patients (Hofmeier et al., 2021; Koops et al., 2021). While numerous studies analyzed slower EEG brain oscillation changes in tinnitus and linked particular alpha band oscillation changes with tinnitus (Weisz et al., 2007; Ortmann et al., 2011; Leske et al., 2014; Sturm and Weisz, 2015; Li Y. H. et al., 2022), the role of gamma oscillations in auditory cortex of tinnitus subjects remained up until now rather unclear and partially controversial. This is most likely due to the fact that EEG studies with tinnitus subjects have not differentiated between those with and without hyperacusis. Also, have EEG studies in T-subjects yet to be performed so far under conditions that tested for differences in brain oscillations to actively perceived auditory stimuli within and outside their tinnitus frequency range, as applied here. The pitch discrimination task requires participants to pay close attention but avoids frustration as the difficulty level is modified based on their performance in real-time. The method thereby intended to identify potential subclass-specific differences in audibility level, attention, and working memory during the performance of the task. The main finding in left temporal cortex upon continuous-wavelet-transformed based analyses of oscillatory events was a significantly higher spontaneous high gamma oscillation [61–120 Hz] in T- compared to C-group, while spontaneous low gamma oscillations [21–40 Hz], which previously were reported to be enhanced in tinnitus subjects (van der Loo et al., 2009; Vanneste et al., 2010, 2018), in our small sample only showed a trend to be higher in T (Figure 8A). In contrast, evoked gamma oscillations [41–60 Hz] were significantly lower in T than in TH, while there was only a trend compared to C (Figure 8B). These differences in the gamma frequency range were associated with a hemodynamic correlate measured with fNIRS: we saw reduced deoxy-Hb activity in temporal cortex in the T-group, while the TH-group expressed increased deoxy-Hb activity (Figures 8C,D). For T, we here suggest, that the deoxy-Hb activity reduction associated with enhanced spontaneous and reduced evoked gamma band power in AC may have its origin in altered synchronous activity in cortical representations of the tinnitus frequency, which not only has a potential to influence representations of lower frequencies (Marcher-Rorsted et al., 2022), but also—together with the oscillatory power in (high) gamma bands—predicts hemodynamic responses (Oya et al., 2018). We do not have direct evidence for altered synchronization, but this could be a potential next step to confirm this important concept. Conversely, in the TH-group that also exhibits reduced fast (high-SR) auditory fiber processing at fTin, a proposed multiplicative neural gain possibly driven by deafferentation of OHCs (Knudson et al., 2014; Sturm and Weisz, 2015) may be the consequence of inadequately high attention (see interpretation of temporoparietal junctions below). Equally important, the salience of the acoustic stimuli in hyperacusis (see above) may be expressed by the stronger evoked gamma responses at fRef (Figure 8B).

Enhanced spontaneous gamma in the AC, as observed here in the T-group, is compatible with animal studies that demonstrated rapidly enhanced spontaneous neuronal firing in response to induced chronic tinnitus, which was linked to neuronal bursting and excessive neuronal synchrony in AC (Noreña and Farley, 2013). This abnormal neural synchrony has been suggested to be confined to specific oscillation frequency bands in tinnitus subjects (Eggermont and Tass, 2015), particularly to enhanced spontaneous gamma oscillations. Altered spontaneous gamma oscillations in tinnitus were also reported in the range of [30–80 Hz], corresponding to our mid gamma frequencies [41–60 Hz] and part of the low [21–40 Hz] and high [61–120 Hz] gamma band, according to previous human (Weisz et al., 2007; Ortmann et al., 2011) as well as animal studies (Tziridis et al., 2015). In all cases up to now, the enhanced spontaneous gamma oscillations in tinnitus patients have been proposed to be the consequence of overactive feedback loops (De Ridder et al., 2015; Sedley, 2019). In contrast, we here suggest that the observed increased cortical synchrony in tinnitus subjects may result from underactive tonic feedback inhibition: this would not only explain enhanced spontaneous gamma oscillations (Figure 8A) but also provide a rationale for the observed reduced evoked gamma oscillations in AC (Figure 8B) being associated with reduced fNIRS-deoxy-Hb activity as here observed in T-subjects (Figures 8C,D). Given that PV+ inhibitory interneurons are critical for generating both gamma (feed-forward inhibition) and beta frequency oscillations (feedback inhibition; Cardin et al., 2009; Sohal et al., 2009; Chen G. et al., 2017), we reasoned that in tinnitus, a pathological reduction of tonic (perisomatic) inhibition of cortical pyramidal neurons by monosynaptically coupled PV+ interneurons in auditory circuits may have occurred as a result of a critical reduction of fast (high-SR) auditory processing in tinnitus frequency regions (Knipper et al., 2020). Under these conditions, pyramidal neurons would fire synchronously and independently of input, as shown for cerebellar neurons (Duguid et al., 2012) and cortical neurons during epilepsy (Rossignol et al., 2013; Hsieh et al., 2017). This would provide an explanation for the observed elevated spontaneous gamma power observed in present and previous studies (Vanneste et al., 2019). If indeed through reduced driving force for the activation of fast-spiking PV+ interneuron activity and subsequent breakdown of PV-dependent attention-driven contrast amplification processes (Siegle et al., 2014; Kim et al., 2016; Chen G. et al., 2017; Galuske et al., 2019), impaired “noise cancelation” leads to tinnitus as suggested (Rauschecker et al., 2015; Knipper et al., 2020), requires future validation.

In this context, our finding that left temporal cortex of tinnitus subjects was less well-coupled to thalamic input, expressed less hemodynamic activation and showed a strong spontaneous gamma enhancement (d = 0.98) and reduced evoked gamma [41–60 Hz] oscillations is of particular interest, as several studies underscored a left–right hemispheric asynchrony and left hemisphere deficits to be linked with attention deficits (Serrallach et al., 2022). As we did not detect any of these effects in right temporal cortex, despite equal distribution of tinnitus laterality and a significant fraction of bilateral tinnitus percepts, we consider left temporal cortex as the primary hub for active auditory processing affected by T and TH.

In the following sections, we will discuss four other cortical hubs which revealed significant effects and thus appeared relevant for our small sample and that might be seen in the context of previously described tinnitus-related changes in, e.g., the connectivity in attentional circuits (Husain, 2016; Schmidt et al., 2017), emotional-distress regions (Husain, 2016; Leaver et al., 2016a; Chen Y. C. et al., 2017), or temporo-frontal attentional networks (Schlee et al., 2009; Vanneste and De Ridder, 2012; Rauschecker et al., 2015; Leaver et al., 2016b; Schmidt et al., 2017). However, none of the previous studies differentiated for co-morbid hyperacusis or have used supra-threshold ABR wave analysis in combination with analysis of rs-fMRI-bfc, fNIRS, and EEG gamma oscillation power. Therefore, the T- and TH-group specific differences in the functional connectivity parameters, evoked or induced hemodynamic responses, and gamma oscillation power between different subtypes, described in the next chapter, may have escaped detection until now.

### Functional connectivity and neural processes in right parietal cortex

Given the pattern in functional connectivity presented in Figure 7, the temporal and parietal cortex shows reduced rs-fMRI-bfc with their respective input structures, as are the MGB and AC. Here, the parietal cortex – like the temporal cortex – differs from frontal and prefrontal regions in that their overall rs-fMRI-bfc shows a significant reduction for T compared to C but not for TH. In contrast, frontal regions covering the inferior frontal gyrus (around F7) and the dorsolateral prefrontal cortex (around F3) expressed an inverse pattern, with TH showing reduced functional connectivity to the AC, while T and C were indistinguishable (Figures 7D,E). The lateral inferior parietal cortex (around P4) was the only region on the right hemisphere to show any significant differences for enhanced spontaneous low gamma (T > C) with the largest effect size (d = 1.258) we could observe in our sample. Furthermore, evoked gamma oscillations seemed to play no role in this attention steering area, while induced gamma oscillations [41–60 Hz] showed the largest effect size (d = 0.78) obtained for any differences of task-induced gamma being reduced in TH in comparison to T (Figure 9B). This may suggest that induced gamma oscillations may serve a different purpose than evoked gamma during auditory processing. Most importantly, this right parietal region expressed a strong hemodynamic oxy-Hb-response for stimuli at reference and tinnitus frequencies in T- but much less in the TH-group (Figure 9C; Supplementary Figure 12). Within this context, it may be interesting to contemplate previous findings of altered low brain oscillations of the right parietal cortex that was shown to interfere with the attending left parietal cortex during an attention-requiring auditory task (Deng et al., 2019). These studies suggested that, among others, hemispheric and task-specific effects of the parietal cortex play a role in top-down control of auditory attention (Deng et al., 2019). Here, the fact that the EEG electrode P4 picks up signals from BA40 also needs consideration. The BA40 region is not only involved in language and category judgments (Chen et al., 2019) but is also related to parietal attention steering and provides a component for limbic associational integration (Williams et al., 2000). In conclusion, the observed differences in brain oscillation and hemodynamic response changes in the right parietal cortex between T- and TH-groups should be considered in the context of previous suggestions that reported the involvement of attention differences in tinnitus (Roberts et al., 2013; Shore et al., 2016; Sedley, 2019), however, neither of these studies did distinguish between T and TH.

### Functional connectivity and neural processes in the left parietal cortex

For areas in the left parieto-temporal junction, we observed a more pronounced reduction of rs-fMRI connectivity in the T- and TH-group between the AC and the regions that extended into Wernicke’s area. This region comprises the posterior part of BA21, 22 and BA39, 40 (Figure 7C; Supplementary Figure 7C), encompassing the angular gyrus near the junction of temporal, occipital, and parietal lobes. The reduction in rs-fMRI in the T- and TH-group was linked with a differentially reduced and elevated spontaneous activity in gamma bands and differential elevated and reduced evoked gamma in the TH-group. The fNIRS responses in the TH-group showed a decline of oxy-Hb compared to controls, while the deoxy-Hb activity is increased in T- and even more pronounced in TH-group (Figures 10C,D; Supplementary Figure 12). Compared to the above findings, the rather complex relationships are likely due to the different functions of the “extended” Wernicke region, which comprise a role in sound detection and language processing (Ardila et al., 2016). The differentially altered hemodynamic (Figures 10C,D) and gamma oscillatory power in the T- and TH-group (Figure 10B) may also be associated with a specific role of BA39 that if damaged, has been reported to result in dyslexia or semantic aphasia (Ardila et al., 2016). Overall, these differential changes in gamma oscillations and hemodynamic responses may be considered in the context of differential speech comprehension difficulties in quiet and noise environments between tinnitus subjects with and without hyperacusis (Vielsmeier et al., 2016). Based on the reduced rs-fMRI correlation between AC and the left hemispheric Wernicke region (Figure 7C), we need to consider the altered gamma power which dominated the homologous region in the right hemisphere (Figure 9). One possible interpretation of the differences between right and left parieto-temporal junction is that the parietal cortex in the right hemisphere can interfere with attending left stimulation patterns, as shown during attention-requiring auditory stimulation tasks (Deng et al., 2019). As the parietal cortex is considered a global brain-wide control node for attention (Behrmann et al., 2004), the observation of more spontaneous low gamma expressed on the right side of the parietal cortex (Figure 9) can be regarded to serve as a process driving attention to transform the concomitant sensory processes to phantom percept, as also suggested in a computer-based model (Sedley, 2019). In addition, the reduced spontaneous low gamma and increased mid gamma on the left side in TH (Figure 10A) may indicate some compensation or protection of the cortical circuits in the language dominant hemisphere. This is highly speculative but represents a concept that could be tested in a follow-up study. In conclusion, our study revealed reduced low gamma power during EEG resting state, along with a tendency towards increased gamma power evoked by tinnitus frequency in the P3 region of the TH-group. Additionally, there was a tendency towards increased brain activity in response to tinnitus frequency in TH-group. The findings in the P3 region—as a part of the executive frontoparietal central network—suggest altered neural processing and impaired cognitive control mechanisms in our TH-group (Seeley et al., 2007; Vincent et al., 2008), highlighting the distinct nature of this condition compared to tinnitus alone.

### Functional connectivity and neural processes in the left ventrolateral prefrontal cortex

Again, based on rs-fMRI-bfc, we observed a reduced frequency of positive correlation between AC and the ventrolateral prefrontal region in the T- less than in the TH-group (Figure 7D; Supplementary Figure 7D). The ventrolateral prefrontal cortex consists of a more caudal opercular part, including the inferior frontal gyrus (BA44), a more rostrodorsal-mid-frontal (BA46) and rostroventral orbital part of the inferior frontal gyrus (BA47), a more frontal part (BA45), with BA44 and BA45 comprising Broca’s area (see for a review: Nozari and Thompson-Schill, 2016). Although numerous studies reported complex changes in BOLD rs-fMRI activity in tinnitus patients in these regions (Hussain et al., 2016; Leaver et al., 2016a; Chen et al., 2018), up to now, no study examined multimodal imaging results to cross-validate brain activity changes in tinnitus, and no previous study distinguished between tinnitus subjects with and without hyperacusis. These reasons most likely explain the partially inconsistent findings compared to previous studies. Along with reduced rs-fMRI-bfc between AC and ventrolateral prefrontal cortex in TH, less than T (Figure 7D), we observed elevated spontaneous low/mid gamma and reduced evoked mid-frequency gamma power in the T- compared to the TH-group when stimulating with fRef (Figure 11B). FNIRS responses to stimulation within fRef led to increased deoxy-Hb activity in the T-group (Figure 11C). These findings point to diminished neural synchronization during active auditory discrimination upon stimulation within lower reference frequency ranges in T, less in TH associated with reduced hemodynamic responses in the T-group, less in the TH-group.

If we want to understand this differential finding in T- vs. TH-groups in response to the attention-demanding two-tone discrimination task, we need to recall the complex function of the ventrolateral prefrontal cortex briefly. The pitch discrimination task required exact recognition of categorical semantic information. The ventrolateral prefrontal cortex serves, among other functions (see for a review: Nozari and Thompson-Schill, 2016), the temporal sequencing of signals regardless of their specific stimulus type (Gelfand and Bookheimer, 2003; Hickok and Poeppel, 2007) and controlled semantic processing (Wagner et al., 2001). In light of our findings, these functions could mean that T-subjects might have more difficulty following rapid loudness sequences than TH-subjects due to their decreased activation of brain regions required to focus on proper fast phonetic decoding. While further studies will be required to support this notion, this finding may be worth discussing in the context of speech comprehension difficulties (Bures et al., 2019; Zeng, 2020; Sendesen and Turkyilmaz, 2023), increased intensity discrimination thresholds in T-subjects (Epp et al., 2012), reduced attention shifts in response to an auditory change in tinnitus (Cuny et al., 2004), and previous finding which reported more pronounced difficulties in T- than in TH-subjects (Vielsmeier et al., 2016). However, the differential activation of the ventrolateral prefrontal region in T- and TH-subjects following the attention-demanding discrimination task should also be discussed in connection with the frequently co-morbid depression of tinnitus subjects (for review Pattyn et al., 2016; Durai et al., 2019; Hebert, 2021). Indeed have various studies shown a specific deactivation and functional disconnection of superior external frontal cortex (BA46) or inferior frontal gyrus (BA45) regions, as part of ventrolateral prefrontal regions to be tightly correlated with elevated depressive disorder (Pizzagalli and Roberts, 2022).

### Functional connectivity and neural processes in the left dorsolateral prefrontal cortex

The connectivity of subjects with tinnitus with and without hyperacusis is generally characterized by reduced frequency of rs-fMRI-based correlations between AC and other cortical regions. However, there is a specific difference between T and TH, which concerns functional connectivity with the frontal cortex: in particular, connectivity between AC and prefrontal cortex shows significantly stronger effects for TH than for T, the latter showing at most a trend compared to the C-group (Figure 7E; Supplementary Figure 7E). The reduction of functional connectivity for TH is highly significant for BA9, although the differences are also significant for the T-group. If we differentiate the groups for AC correlations to BA9DL and BA9M (Supplementary Figures 7F,G), it, however, turns out that in the TH-group, the reduced connectivity is preferentially limited to the BA9DL, while the connectivity to the BA9M appears to be increased (Supplementary Figures 7F,G). Associated with these differences in connectivity are reduced spontaneous and a trend for increased evoked high gamma also restricted to the TH-group (Figures 12A,B). It is challenging to suggest that decreased spontaneous and enhanced evoked high gamma power may instead be linked to BA9M than BA9DL, but our current EEG and fNIRS methods do not allow for differentiation or may even be entirely insensitive for BA9M.

While without doubt, further studies with a larger group size will be required to validate this hypothesis, we may reconsider the overall accepted role of prefrontal cortex region BA9 involved in distress regulation (Lai et al., 2019; Jacobs and Moghaddam, 2021; Kupferschmidt et al., 2022; Li L. et al., 2022), that may imply positive regulation of the BA9DL for stress balancing (Sullivan and Gratton, 2002) and BA9M for stress excitation (McKlveen et al., 2013; Utevsky and Platt, 2014; McKlveen et al., 2016). The reduced rs-fMRI connectivity from AC to BA9DL in TH may be related to the hemodynamic activity observed in response to tinnitus frequencies (fTin) in TH-subjects, where oxy-Hb in the TH-group showed a profound increase (Figure 12C). In particular, with regard to the P3 results, the observed reduced low/high gamma power during EEG resting state, along with a tendency towards increased gamma power evoked by tinnitus frequency in the F3 region of the TH-group. In previous literature, the correlation strength of the frontoparietal executive resting state network is reduced in tinnitus (Kandeepan et al., 2019; Zhou et al., 2019). But we observe a significant reduction in both frontoparietal executive network regions (P3 and F3), especially in the TH-group, coupled with a tendency for increased tinnitus frequency evoked gamma. Our interpretation of the observed changes in the frontoparietal executive resting state network in the TH-group suggests an impaired cognitive control mechanism. These impairments are evident not only during resting state, but also during auditory tasks within the individual tinnitus frequency range, highlighting the relevance of considering both resting state and task-evoked conditions in understanding the neural correlates of hyperacusis in tinnitus.

In conclusion, we propose that the reduced rs-fMRI connectivity changes between AC and BA9 in TH are dominated by a reduction in connectivity to BA9DL at rest and increased evoked hemodynamic response in fNIRS to stimulus in fTin: As a result, the described increased stress response to the tinnitus loudness in TH-subjects (present study, Aazh et al., 2019; Kula et al., 2023) could be explained by an increase in the BA9DL controlled stress balance (Sullivan and Gratton, 2002). This hypothesis would be strengthened by the reduced spontaneous and increased evoked gamma power in the TH-group in response to stimulation with fTin. In case we predict this to be linked to enhanced connectivity of AC to BA9M (Supplementary Figure 7G), that activity could lead to enhanced stress excitement (McKlveen et al., 2013; Utevsky and Platt, 2014; McKlveen et al., 2016).

## Limitations

This pioneering study has several limitations which should be acknowledged. First, the number of subjects included in the analysis is low, which limits the generalizability of our findings. The rather limited use of psychometric data such as Beck’s depression inventory (BDI) for our analyses may hinder a more comprehensive understanding of the subgroups. Moreover, the use of a 21-channel EEG system severely restricts source localisation, making it at least challenging to identify brain regions involved in tinnitus and hyperacusis. Furthermore, the study is limited by a lack of fine-structured DPOAE function measurements in more abundant frequency channels, required to exclude subtle electromechanical property differences in narrow frequency channels. Lastly, due to its pioneering character, the study lacks (double-)blinding, which may introduce biases to behavioral performance, analyses, and their interpretation. These limitations should be considered when interpreting our results, and should be avoided in future research.

## Perspectives

We found evidence for explaining the differences between the T- and TH-group, particularly in their differential response to tinnitus loudness, which appeared to be related to differential cortical brain activity either leading to or at least being able to predict differential distress. The key changes encompass reduced connectivity between ascending auditory circuits up to the AC as a feature of T, less so of TH. In contrast, the distinct differences in activation of left temporoparietal and left dorsolateral prefrontal cortex (P3 and F3) in TH need to be considered in the context of predicted excitation spreading to limbic and pain regions, which among other consequences may result in inadequately high attention to increased loudness at all sound frequencies, in line with previous suggestions (Knudson et al., 2014; Sturm and Weisz, 2015; Sedley, 2019; Knipper et al., 2020; Zeng, 2020; Refat et al., 2021). Differences in attention between tinnitus with and without hyperacusis may explain numerous controversial findings concerning listening effort and speech comprehension in tinnitus (Vielsmeier et al., 2016; Ivansic et al., 2017; Jagoda et al., 2018; Bures et al., 2019; Liu et al., 2020; Zeng, 2020; Niewiarowicz et al., 2022; Tai et al., 2023).

Insights into the basis of differential neural correlates of tinnitus and hyperacusis have been gained which provide new dimensions for clinical therapeutic concepts. Therefore, the importance of inquiring about hypersensitivity to noise during the anamnesis should be emphasized.

Overall conclusion: (i) Based on the outcome of this study, we recommend future multicenter studies to validate our findings with larger cohorts. In order to obtain responses to subjectively equally loud stimuli, we recommend a harmonized methodology consisting of audiometric stimulation normalized to individual sensation level, identified frequency ranges of pure tone audiometry, resting state and evoked fMRI conditions, and EEG stimulation protocols. (ii) Future multicenter studies should compare a variety of methodologies, such as resting state and evoked EEG, fNIRS, and fMRI, in order to determine the utility of the different methods in identifying subclassifications of tinnitus. (iii) It may additionally be of crucial importance to identify characteristic signatures of the subclassifications of tinnitus, such as a reduced signal-to-noise ratio in a distinct frequency channel (a characteristic feature of tinnitus alone), or an enhanced signal-to-noise ratio in a parallel frequency channel (a characteristic feature of tinnitus with co-occurring hyperacusis). (iv) Finally, the authors recommend using a more precise tinnitus localisation method, i.e., tinnitus frequency and bandwidth matching, in addition to standard clinical protocols to achieve a more accurate stimulation in the frequency.

## Data availability statement

The original contributions presented in the study are included in the article/Supplementary material, further inquiries can be directed to the corresponding author.

## Ethics statement

The studies involving humans were approved by Ethics Committee Tübingen, Chairman: Karl Jaschonek, Tübingen University Clinical Centre, Gartenstr. 47, 72074 Tübingen, Germany, Tel: 07071-2977661, Fax: 07071-295965, E-mail: ethik.kommission@med.uni-tuebingen.de. The studies were conducted in accordance with the local legislation and institutional requirements. The participants provided their written informed consent to participate in this study.

## Author contributions

MK, LR, SW, and MM designed the research. JW, KD, JS, ED, KB, BB, and RS conducted the experiments. JW, BB, RS, ED, and KB analyzed the data. MK, MM, and SW wrote the first draft of the paper. MK, MM, LR, WS, and UK edited the paper. All authors contributed to the article and approved the submitted version.

## Glossary

1/2/3…22: Channels of NIRS optode montage
AAN: Ascending auditory nuclei
ABR: Auditory brainstem response
AC: Auditory cortex
AC-I: Primary auditory cortex
ANF: Auditory nerve fibers
BA: Brodmann area
BOLD: Blood oxygenation level-dependent
C: Control
CN: Cochlear nucleus
d: Effect size (Cohen’s d)
deoxy-Hb: Deoxygenated hemoglobin
DL: Dorsolateral
EEG: Electroencephalography
fc: Functional connectivity
FIR: Finite impulse response filter
fMRI: Function magnetic resonance imaging
fNIRS: Functional near-infrared spectroscopy
Fpz: ABR electrode montage (ground)
fRef: Reference-frequency tone stimulus
fTin: Tinnitus frequency tone stimulus
GHS: Goebel-Hiller-Score
Hb: Hemoglobin
High-SR: High-spontaneous firing rate
HKI: Hyperacusis inventory
HL: Hearing level
IC: Inferior colliculus
LH: Left hemisphere
M: Medial
MGB: Medial geniculate body
MTG: Middle temporal gyrus
NIRS: Near-infrared spectroscopy
ns: Not (statistically) significant
OHC: Outer hair cells
OLSA: Oldenburger Satztest
oxy-Hb: Oxygenated hemoglobin
pDPOAE: Pulsed distortion-product otoacoustic emissions
PFC: Prefrontal cortex
PTA: Pure tone audiogram
PTA4: Pure tone audiometry threshold average 0.5 – 4 kHz, better ear
PTA-EHF: Pure tone audiometry threshold average 11.2 – 16 kHz, better ear
PTA-HF: Pure tone audiometry threshold average 6 – 10 kHz, better ear
PTA-T: Pure tone audiometry threshold at tinnitus frequency
RH: Right hemisphere
ROI: Regions of interest
rs: Resting state
rs-fMRI-bfc: Resting-state-fMRI-based functional connectivity
SD: Standard deviation
SL: Sensation level
SOC: Superior olivary complex
SPL: Sound pressure level
STG: Superior temporal gyrus
T: Tinnitus
T7 P3 F3 F7: EEG electrode montage left hemisphere according to 10-20-system
T8 P4 F4 F8: EEG electrode montage right hemisphere according to 10-20-system
TH: Tinnitus with hyperacusis
LDL: Loudness discomfort levels
