# Supplementary material for: Differential cortical activation patterns: pioneering sub-classification of tinnitus with and without hyperacusis by combining audiometry, gamma oscillations, and hemodynamics
Source: Front Neurosci. 2024 Jan 4;17:1232446. doi: 10.3389/fnins.2023.1232446 (PMC10794389; doi:10.3389/fnins.2023.1232446)
Supplement: Supplementary file 1 [file Data_Sheet_1.docx]

Supplementary Material

**Differential cortical activation patterns: pioneering sub-classification of tinnitus with and without hyperacusis by combining** **audiometry, gamma oscillations, and hemodynamics**

**Jakob Wertz^1^, Lukas Rüttiger^1^, Benjamin Bender ^2^, Uwe Klose^2^, Robert S. Stark^3^, Konrad Dapper^1^, Jörg Saemisch^1^, Christoph Braun^7^, Wibke Singer^1^, Ernst Dalhoff^4^, Katharina Bader^4^, Stephan M. Wolpert^5^, Marlies Knipper^1^**, Matthias H. J. Munk^3,6^****

**** Correspondence:** Corresponding Author:

Prof. Dr. Marlies Knipper [marlies.knipper@uni-tuebingen.de](mailto:marlies.knipper@uni-tuebingen.de)

# Supplementary Methods

## Audiological evaluation

Ear examination was carried out by the study ENT physicians in the outpatient Ear, Nose, and Throat (ENT) Department of the University Clinic Tübingen. The ear canal was cleaned from wax, and the ear canal and the tympanic membrane were inspected to exclude external canal anomalies or tympanic membrane pathologies.

Tympanometry was performed in the Audiology Unit of the ENT Department with the AT235 (Interacoustics, Middelfart, Denmark) with 226 Hz and 300 daPa to ensure standard middle ear transmission.

Hearing thresholds were determined by pure tone audiometry (PTA) with the Audiometer AT1000 (Auritec, Medizindiagnostische Systeme GmbH, Hamburg, Germany) in a soundproof chamber (Industrial Acoustics, Niederkruchten, Germany). On-ear headphones (AT 1350 A, Beyerdynamic, Heilbronn, Germany) were used for low frequencies, and over-ear headphones (HDA 300, Sennheiser, Wedemark, Germany) were used for frequencies above 10 kHz. The measured frequencies include 0.125, 0.25, 0.5, 1, 2, 3, 4, 6, 8, 10, 11.2, 12.5, 14, and 16 kHz.

For subjects with tinnitus, the two-alternative forced choice (2AFC) recursive matching method was utilized to identify Tinnitus pitch and loudness of individual ears. In this method, a series of acoustic sinus stimuli were presented to the patient, and they were asked to indicate whether the tinnitus was higher/lower in pitch or louder/quieter after each stimulus. The procedure was repeated to obtain the most accurate determination of tinnitus frequency and loudness (Ernon and Fenwick 1983).

Additionally, speech audiometry was performed using the German version of the matrix test, the "Oldenburger Satztest" (OLSA), with on-ear headphones (AT 1350 A, Beyerdynamic, Heilbronn, Germany). OLSA assesses the speech reception threshold by repeating random German five-word sentences. Thereby, the subject is instructed to repeat a series of five-word German sentences, and the dB level at which they can repeat 50% of the words correctly is defined as the speech reception threshold. Our OLSA test utilizes standardized speech stimuli and includes two test phases: binaural presentation of speech signals without noise and monaural presentation of speech signals with contralateral speech-shaped noise at 65 dB. The resulting data comprehensively assesses the individual's speech recognition abilities in noisy conditions (Frank and Karlovich, 1975).

## Questionnaires

To assess the presence of hyperacusis and to differentiate it from phonophobia/recruitment, a Hyperacusis Questionnaire (HKI) (Fischer, 2013) was administered to all subjects. The HKI was developed based on the "Hyperacusis Questionnaire" (HQ) (Khalfa et al., 2002) and the "Geräusch Überempfindlichkeits Fragebogen (GÜF)" (Nelting et al., 2002) and consists of 9 statements which are evaluated with a score from zero to two (Supplementary Figure 14).

The tinnitus questionnaire Goebel-Hiller-Score (GHS) evaluated various aspects of tinnitus severity, such as emotional distress, cognitive distress, self-experienced intrusiveness, and auditory perceptual difficulty scores (HillerGoebel and Rief, 1994). The questionnaire consists of 52 statements that tinnitus subjects had to answer: true, partially true, or not true. The analyzed GHS scores were then correlated with the tinnitus loudness levels to determine which aspects were related to tinnitus intensity in the audiological evaluation.

## Pulsed distortion-product otoacoustic emissions (pDOAEs) & Békésy-tracking audiometry (BT)

Behavioral thresholds were obtained with modified Békésy-tracking audiometry (BT) and estimated distortion-product thresholds (EDPTs) derived from DPOAE level maps based on pulsed DPOAE were recorded. For both BT and EDPTs, ER-10C DPOAE in-ear probe microphone system (Etymotic Research, Elk Grove Village, IL, USA) was used in a double-walled sound-proof chamber (Industrial Acoustics Company, Niederkrüchten, Germany). Analysis was performed upon a custom-built toolbox implemented in LabVIEW (Version 17.0, National Instruments, Austin, TX) and a custom-built toolbox developed with MATLAB (Version 9.6, The MathWorks, Natick, MA, USA). BT were obtained for 11 frequencies in the range 1 – 12 kHz (2, 4, 8, 8.5, 9, 9.5, 10, 10.5, 11, 11.5, and 12 kHz). DPOAEs were recorded for *f*_2_ = 2, 4, 8, 10, and 12 kHz with *f*_2_/*f*_1_ = 1.2. A single acquisition block was averaged until a signal-to-noise ratio (SNR) of 50 dB or a maximum number of 200 repetitions was reached. The procedures are described in detail by Bader et al. (2021). The estimation of the amplitude of the nonlinear-distortion component uses a technique called onset decomposition to minimize interference between the nonlinear-distortion and coherent-reflection components and, thereby, increases the precision of the level estimate of the nonlinear-distortion component and, therefore, of information about the functionality of the cochlear amplifier (Vetesnik et al., 2009; ZelleDalhoff and Gummer, 2017).

At each *f*_2_, 20 pDPOAEs were measured with different predefined stimulus level pairs (*L*_1_*, L*_2_), scanning the individual DPOAE growth behavior in the *L*_1_, *L*_2_ space and enabling the reconstruction of an individual model level map (DPOAE level map) for a specific frequency *f*_2_. DPOAE level maps allow the derivation of EDPTs using a mathematical model of a level map fitted to the experimental data. The EDPT is defined as the value of *L*_2_ at the intersection of the extrapolated ridge with the *L*_1_*, L*_2_ plane. This method allows EDPTs to be obtained individually without predefining individual and frequency-specific optimal stimulus levels (Zelle et al., 2020). The total recording time for all short-pulse DPOAEs at five frequencies with 20 levels was 10 min. In total, to evaluate the function of the cochlear amplifier for each ear at different *f*_2_, we investigated three pDPOAE metrics 1) EDPTs based on pDPOAE, 2) pDPOAE threshold, and 3) pDPOAE amplitude at *L*_2_ = 50 dB SPL and *L*_1_ = computed by individual optimal path parameters. If no pDPOAE amplitude was measurable at *L*_2_ = 50 dB SPL, a linear interpolation of the neighboring DPOAE amplitudes was performed. Further, if only one DPOAE amplitude per frequency was measurable, a DPOAE amplitude for *L*_2_=50 dB SPL was calculated using the frequency-specific slope based on population data according to (Zelle et al., 2020), as long as the experimental data point was close (<5 dB) to *L*_2_=50 dB SPL.

## Preprocessing rs-fMRI

The rs-fMRI measurement parameters were: echo time (TE) 35 ms, repetition time (TR) 2 s, number of slices 40 with 25% gap, field of view (FOV) 190 x 190 mm² with a matrix of 64 x 51, which was interpolated to 64 x 64, pixel bandwidth 1954 Hz/px, that covered the whole brain and relevant brainstem areas (cochlear nucleus (CN), superior olivary complex (SOC), inferior colliculus (IC)). The participants were instructed to remain alert with eyes closed, with no task to perform. Earplugs were used for all participants during the scan to reduce noise generated by the scanner. After three initial excitations, one hundred fifty volumes were acquired to reach a steady state.

After the transformation of the DICOM images to NIFTI format origin of the anatomic T1 image was set to the center of the anterior commissure with SPM12, the axial plane aligned to the AC-PC line and the head reoriented if necessary to have the mid-sagittal plane split the brain into the two hemispheres. Afterward, the transformation matrix was applied to the functional images. Resting-state fMRI were further processed with dpabi DPARSF Advanced Edition (Version 4.5) (Yan et al., 2016), with the following settings:

- Time Points: 150

- TR (s): 2

- Slice Timing ✓

- Slice Number: 40

- Slice Order: [1:2:39 2:2:40]

- Reference Slice: 20

- Realign ✓

- T1 Coreg to Fun ✓

- New Segment + DARTEL ✓

- Nuisance Covariates Regression ✓ (Polynomial trend: 1)

- Head Motion model: Friston 24

- Head motion scrubbing regressors ✓

- Filter (Hz): 0.01 ~ 0.1

- Normalize using EPI templates (Bounding Box: [-90 -126 -72;90 90 108], Voxel Size [3 3 3])

- Smooth by DARTEL (FWHM: [5 5 5])

- Default mask (SPM 5 apriori mask – thresholded at 50%)

- Extract ROI time courses (Defined ROIs see Supplemental Table 3)

The Pearson correlation coefficients were calculated for each pairwise ROI combination in each subject using the Matlab function *corrcoeff.* Subsequently, the frequency of significant positive (p < .05) rs-fMRI BOLD correlations between predefined ROI clusters (subcortical regions to MGB; MGB to AC-I; AC-I to BA45, BA46, BA47; AC-I to BA9M, BA9DL; AC-I to BA21, BA22, BA39, BA40) was calculated for each subject group (C = controls, T = tinnitus, TH = tinnitus + hyperacusis).

# Supplementary Results

Previous studies reported EHF threshold elevation in tinnitus (Peng et al., 2021; Song et al., 2021) that were potentially associated with some degree of cochlear mechanical dysfunction (Jafari et al., 2022). To explore the impact of age on PTA-EHF threshold trends, we examined age-related threshold differences between T- and TH-groups in an independent group. In a first pilot experiment pulsed distortion-product otoacoustic emissions (pDPOAEs) and behavioral thresholds were obtained with Békésy-Tracking audiometry up to 12 kHz in a small subset of 12 controls (25.8 ± 2.9 years) and six subjects with either T or TH (35.5 ± 11.2 years) (Supplementary Figure 2A). Three pDPOAE metrics measured up to 12 kHz, i.e., the pDPOAE amplitudes (Supplementary Figure 2B), pDPOAE thresholds (lowest measurable pDPOAE at a given frequency), and estimated distortion-product thresholds (EDPTs) (Supplementary Figure 2C), are in accordance with the Békésy-Tracking thresholds of the
C-, T-, and TH-groups. When correcting for age, no group differences remained up to 8-12 kHz (Supplementary Figure 2D). Taken together, although based on a very small group size, these data give no indication against two assumptions: 1) behavioral threshold variation in > 8 kHz regions in the enclosed subgroup Gr2 is explained by age, and 2) threshold variations observed are explained by reduced function of the cochlear amplifier.

**In essence**, no significant differences in hearing threshold up to 16 kHz were seen between T- and TH-groups compared to controls, although a larger cohort will be required to validate this observation.

# Supplementary Figures and Tables

## Supplementary Tables

## Supplementary Tables

| **Proband** | **Age** | **Gender** | | **Handedness** | **HKI** | **HKI [Quart]** | **LDL [Quart]** |
| --- | --- | --- | --- | --- | --- | --- | --- |
| Controls | | | | | | | |
| 'TS002' | 31 | male | | right | 5 | None | Mild |
| 'TS003' | 30 | female | | right | 3 | None | Moderate |
| 'TS012' | 19 | male | | right | 4 | None | Mild |
| 'TS014' | 26 | male | | right | 0 | None | Mild |
| 'TS015' | 30 | female | | right | 9 | Mild | Mild |
| 'TS016' | 26 | female | | right | 4 | None | Mild |
| 'TS024' | 27 | male | | right | 4 | None | None |
| 'TS025' | 27 | male | | right | 4 | None | Mild |
| 'TS027' | 24 | female | | right | 2 | None | Mild |
| 'TS028' | 26 | male | | right | 9 | Mild | Mild |
| 'TS029' | 45 | female | | right | 7 | None | Mild |
| 'TS030' | 27 | male | | right | 1 | None | Mild |
| 'TS039' | 26 | female | | right | 7 | None | Mild |
| 'TS041' | 25 | female | | left | 4 | None | Moderate |
| 'TS042' | 30 | male | | right | 3 | None | Mild |
| 'TS047' | 28 | female | | right | 7 | None | Mild |
| 'TS056' | 22 | male | | right | 3 | None | Mild |
| 'TS060' | 24 | female | | right | 3 | None | Mild |
| 'TS063' | 35 | female | | right | 2 | None | Moderate |
| 'TS071' | 27 | female | | right | 6 | None | Moderate |
| 'TS072' | 33 | male | | right | 6 | None | Mild |
| 'TS074' | 36 | male | | right | 1 | None | None |
| 'TS076' | 26 | male | | right | 3 | None | Mild |
| 'TS077' | 31 | male | | right | 11 | Mild | None |
| 'TS078' | 28 | female | | right | 2 | None | Moderate |
| 'TS083' | 25 | female | | right | 4 | None | None |
| 'TS084' | 30 | female | | right | 2 | None | Mild |
| 'TS089' | 27 | female | | right | 4 | None | Moderate |
| 'TS099' | 23 | male | | right | 2 | None | None |
| 'TS100' | 28 | female | | right | 2 | None | Moderate |
| 'TS103' | 25 | female | | right | 1 | None | None |
| 'TS107' | 27 | female | | right | 3 | None | None |
| 'TS131' | 39 | female | | right | 3 | None | Moderate |
| 'TS145' | 25 | male | | right | 6 | None | Moderate |
| 'TS146' | 25 | male | | left | 3 | None | Mild |
| 'TS150' | 25 | female | | right | 4 | None | Moderate |
| 'TS151' | 24 | female | | left | 0 | None | None |
| 'TS153' | 25 | female | | right | 4 | None | Mild |
| 'TS154' | 22 | male | | right | 5 | None | Mild |
| **Proband** | **Age** | **Gender** | **T lateral.** | **Handedness** | **HKI** | **HKI [Quart]** | **LDL [Quart]** |
| Tinnitus | | | | | | | |
| 'TS004' | 44 | male | left | right | 4 | None | Moderate |
| 'TS005' | 29 | male | bilateral | right | 6 | None | None |
| 'TS010' | 26 | male | bilateral | right | 7 | None | None |
| 'TS017' | 29 | male | bilateral | right | 9 | Mild | Mild |
| 'TS021' | 27 | male | bilateral | right | 2 | None | Mild |
| 'TS031' | 50 | male | bilateral | right | 8 | None | Moderate |
| 'TS036' | 27 | female | right | right | 4 | None | Mild |
| 'TS044' | 20 | male | left | right | 17 | Moderate | None |
| 'TS049' | 26 | male | bilateral | right | 5 | None | None |
| 'TS054' | 21 | female | bilateral | right | 9 | Mild | None |
| 'TS057' | 33 | male | bilateral | right | 15 | Moderate | Moderate |
| 'TS059' | 35 | female | bilateral | right | 11 | Mild | Mild |
| 'TS062' | 36 | male | left | right | 3 | None | None |
| 'TS068' | 25 | male | right | right | 3 | None | Moderate |
| 'TS070' | 20 | female | right | right | 6 | None | Mild |
| 'TS073' | 44 | female | right | right | 6 | None | Mild |
| 'TS079' | 25 | male | bilateral | right | 2 | None | None |
| 'TS080' | 30 | male | bilateral | right | 8 | None | None |
| 'TS082' | 27 | male | right | right | 6 | None | Mild |
| 'TS085' | 29 | male | right | right | 4 | None | Moderate |
| 'TS086' | 22 | female | right | right | 0 | None | Mild |
| 'TS087' | 28 | female | bilateral | right | 6 | None | None |
| 'TS090' | 28 | female | left | right | 6 | None | Moderate |
| 'TS091' | 35 | male | left | right | 0 | None | None |
| 'TS094' | 28 | male | bilateral | right | 11 | Mild | Mild |
| 'TS095' | 27 | male | bilateral | right | 8 | None | Mild |
| 'TS097' | 33 | male | left | right | 10 | Mild | Mild |
| 'TS098' | 32 | female | bilateral | right | 1 | None | Mild |
| 'TS121' | 51 | female | bilateral | right | 13 | Mild | Mild |
| 'TS137' | 29 | male | bilateral | right | 10 | Mild | Mild |
| **Proband** | **Age** | **Gender** | **T lateral.** | **Handedness** | **HKI** | **HKI [Quart]** | **LDL [Quart]** |
| Tinnitus & Hyperacusis | | | | | | | |
| 'TS020' | 24 | female | left | right | 12 | Mild | Moderate |
| 'TS033' | 49 | male | bilateral | left | 15 | Moderate | Moderate |
| 'TS037' | 23 | female | bilateral | right | 22 | Severe | Mild |
| 'TS040' | 23 | female | bilateral | right | 15 | Moderate | Severe |
| 'TS048' | 27 | male | bilateral | right | 18 | Moderate | Severe |
| 'TS050' | 21 | female | bilateral | right | 15 | Moderate | Severe |
| 'TS053' | 29 | female | bilateral | right | 11 | Mild | Moderate |
| 'TS061' | 29 | male | bilateral | right | 20 | Severe | Severe |
| 'TS067' | 36 | female | bilateral | right | 25 | Severe | Severe |
| 'TS088' | 24 | male | right | right | 16 | Moderate | Moderate |
| 'TS102' | 36 | female | bilateral | right | 10 | Mild | Moderate |
| 'TS104' | 55 | female | bilateral | right | 16 | Moderate | Severe |
| 'TS105' | 57 | female | bilateral | right | 10 | Mild | Severe |
| 'TS125' | 43 | female | right | left | 16 | Moderate | Severe |
| 'TS140' | 23 | female | bilateral | right | 10 | Mild | Moderate |
| 'TS141' | 25 | male | bilateral | right | 14 | Moderate | Mild |
| 'TS147' | 20 | female | bilateral | right | 9 | Mild | Severe |

**Supplementary Table 1.** Subject information to age, gender, handedness, Hyperacusis self-report, and hyperacusis classification with Hyperacusis Inventory (HKI) and loudness discomfort level (LDL). HKI [Quart], quartile of hyperacusis burden (Goldstein and Shulman, 1996); LDL [Quart], quartile of LDL burden (Berthold-Scholz, 2013); T lateral., Tinnitus laterality.

| **Group** | **Age [years]** | | | **Participants [n]** | | |
| --- | --- | --- | --- | --- | --- | --- |
|  | **Mean** | **Min** | **Max** | **n** | **female** | **male** |
| Total cohort | | | | | | |
| Control | 27.6 | 19 | 45 | 39 | 22 | 17 |
| T-group | 30.5 | 20 | 51 | 30 | 10 | 20 |
| TH-group | 32 | 20 | 57 | 17 | 12 | 5 |
| Gr1 – Subjects included in EHF Audiometry | | | | | | |
| Control | 27.6 | 22 | 39 | 22 | 13 | 9 |
| T-group | 33.5 | 20 | 55 | 18 | 6 | 12 |
| TH-group | 33.7 | 20 | 57 | 11 | 9 | 2 |
| Gr2 – Subjects included in rs-fMRI | | | | | | |
| Control | 28.5 | 23 | 36 | 12 | 7 | 5 |
| T-group | 32.75 | 22 | 55 | 15 | 5 | 11 |
| TH-group | 38.8 | 24 | 57 | 6 | 5 | 1 |
| Gr3 – Subjects included in EEG | | | | | | |
| Control | 28 | 23 | 36 | 13 | 8 | 5 |
| T-group | 32.2 | 22 | 55 | 11 | 5 | 6 |
| TH-group | 37.8 | 27 | 57 | 5 | 5 |  |

**Supplementary Table 2.** Group information of included participants for age and gender. EEG, Electroencephalography; EHF, extended high frequency; rs-fMRI, resting-state functional magnetic resonance imaging.

| **Brain Region** | **MNI Coordinates [mm]** | | | **Radius [mm]** |
| --- | --- | --- | --- | --- |
|  | **X** | **Y** | **Z** |  |
| Subcortical Regions | | | | |
| CN-R/CN-L^1^ | ±10 | -39 | -45 | 3 |
| SOC-R/SOC-L^1^ | ±13 | -35 | -41 | 3 |
| IC-R/IC-L^1^ | ±6 | -33 | -11 | 3 |
| MGB-R/MGB-L^1^ | ±17 | -24 | -2 | 3 |
| Primary Auditory Cortex Regions | | | | |
| BA41-R/BA41-L^2^ | 49/-48 | -13/-20 | 5/7 | 3 |
| BA41A-R/BA41A-L^2^ | 53/-52 | -3/-8 | -2/2 | 3 |
| BA41P-R/BA41P-L^2^ | ±40 | -25/-30 | 10/11 | 3 |
| BA42-R/BA42-L^3^ | ±64 | -22 | 9 | 3 |
| BA42A-R/BA42A-L^3^ | ±60 | -18 | 10 | 3 |
| BA42P-R/BA42P-L^3^ | ±56 | -25 | 12 | 3 |
| Temporal Regions | | | | |
| BA22A-R/BA22A-L^3^ | ±54 | -6 | -6 | 3 |
| BA22P-R/BA22P-L^3^ | ±67 | -27 | 3 | 3 |
| BA21A-R/BA21A-L^3^ | ±66 | -13 | -5 | 3 |
| BA21P-R/BA21P-L^3^ | ±66 | -22 | -5 | 3 |
| BA39-R/BA39-L^4^ | 38/-44 | -76 | 38/32 | 3 |
| BA40-R/BA40-L^4^ | 60/-40 | -30/-46 | 48/44 | 3 |
| Temporo-frontal Regions | | | | |
| BA45-R/BA45-L^5^ | 46/-47 | 26/27 | 7/6 | 3 |
| BA46-R/BA46-L^5^ | 43/-46 | 38 | 12/8 | 3 |
| BA47-R/BA47-L^5^ | 38/-40 | 30/31 | -12/-13 | 3 |
| BA9M-R/BA9M-L^6^ | ±7 | 50 | 30 | 3 |
| BA9DL-R/BA9DL-L^6^ | ±50 | 14 | 32 | 3 |

**Supplementary Table 3.** Predefined ROIs in MNI Coordinates.

^1^(Muhlau et al., 2006)
^2^(Amunts et al., 2020)
^3^(Hofmeier et al., 2018)
^4^(Lancaster et al., 2000)
^5^(Lacadie et al., 2008)
^6^(MaiMajtanik and Paxinos, 2016)

|  | **Right ear LDL [dB HL]** | | | | | | **Left ear LDL [dB HL]** | | | | | |
| --- | --- | --- | --- | --- | --- | --- | --- | --- | --- | --- | --- | --- |
| Freq [kHz] | 0.25 | 0.5 | 1 | 2 | 4 | 6 | 0.25 | 0.5 | 1 | 2 | 4 | 6 |
| Controls | | | | | | | | | | | | |
| TS002 | - | 90 | 95 | 90 | 90 | 85 | - | 85 | 90 | 90 | 90 | 85 |
| TS003 | - | 75 | 75 | 75 | 70 | 65 | - | 80 | 80 | 75 | 80 | 75 |
| TS012 | - | 95 | 90 | 90 | 85 | 95 | - | 95 | 90 | 90 | 80 | 85 |
| TS014 | - | 105 | 100 | 90 | 100 | 90 | - | 110 | 100 | 100 | 100 | 100 |
| TS015 | - | 95 | 95 | 95 | 110 | 110 | - | 95 | 90 | 90 | 110 | 110 |
| TS016 | - | 100 | 90 | 90 | 85 | 85 | - | 90 | 95 | 90 | 85 | 90 |
| TS024 | - | 90 | 100 | 100 | 100 | 100 | - | 100 | 100 | 100 | 110 | 110 |
| TS025 | - | 85 | 90 | 90 | 85 | 90 | - | 85 | 95 | 90 | 90 | 90 |
| TS027 | - | 110 | 90 | 100 | 100 | 100 | - | 100 | 90 | 90 | 90 | 90 |
| TS028 | - | 110 | 110 | 95 | 90 | 90 | - | 105 | 100 | 85 | 90 | 90 |
| TS029 | - | 80 | 80 | 80 | 90 | 100 | - | 110 | 110 | 110 | 110 | 110 |
| TS030 | - | 90 | 90 | 90 | 90 | 90 | - | 90 | 80 | 90 | 80 | 90 |
| TS039 | - | 90 | 90 | 90 | 85 | 85 | - | 85 | 85 | 80 | 75 | 80 |
| TS041 | - | 75 | 70 | 80 | 75 | 75 | - | 75 | 75 | 70 | 75 | 70 |
| TS042 | - | 85 | 85 | 90 | 85 | 85 | - | 85 | 85 | 80 | 85 | 100 |
| TS047 | - | 80 | 80 | 80 | 80 | 80 | - | 90 | 80 | 80 | 80 | 80 |
| TS056 | - | 100 | 100 | 100 | 100 | 110 | - | 80 | 90 | 90 | 90 | 80 |
| TS060 | - | 100 | 90 | 100 | 100 | 100 | - | 100 | 90 | 100 | 95 | 105 |
| TS063 | - | 75 | 75 | 75 | 70 | 65 | - | 70 | 75 | 75 | 70 | 65 |
| TS071 | - | 70 | 70 | 65 | 75 | 70 | - | 75 | 75 | 80 | 75 | 80 |
| TS072 | - | 95 | 90 | 90 | 85 | 90 | - | 80 | 85 | 85 | 85 | 80 |
| TS074 | 110 | 110 | 99 | 95 | 90 | 110 | 110 | 110 | 100 | 105 | 100 | 110 |
| TS076 | 90 | 99 | 95 | 90 | 80 | 86 | 95 | 95 | 90 | 90 | 90 | 110 |
| TS077 | 110 | 97 | 100 | 100 | 99 | 98 | 110 | 110 | 99 | 110 | 110 | 99 |
| TS078 | 110 | 110 | 85 | 91 | 86 | 70 | 110 | 96 | 90 | 91 | 85 | 75 |
| TS083 | 110 | 110 | 110 | 110 | 110 | 110 | 110 | 110 | 110 | 110 | 110 | 110 |
| TS084 | 99 | 101 | 100 | 95 | 95 | 90 | 90 | 94 | 100 | 85 | 95 | 90 |
| TS089 | 100 | 99 | 95 | 86 | 75 | 70 | 90 | 90 | 91 | 80 | 70 | 70 |
| TS099 | 110 | 110 | 105 | 100 | 95 | 100 | 110 | 110 | 105 | 96 | 95 | 95 |
| TS100 | 110 | 105 | 100 | 80 | 66 | 110 | 95 | 95 | 85 | 74 | 75 | 110 |
| TS103 | 110 | 110 | 96 | 103 | 110 | 110 | 110 | 110 | 110 | 100 | 110 | 110 |
| TS107 | 110 | 110 | 110 | 110 | 110 | 110 | 110 | 110 | 110 | 110 | 110 | 110 |
| TS131 | 86 | 95 | 95 | 82 | 70 | 74 | 92 | 110 | 110 | 88 | 89 | 75 |
| TS145 | 61 | 65 | 60 | 62 | 65 | 61 | 77 | 85 | 70 | 75 | 71 | 65 |
| TS146 | 110 | 87 | 82 | 79 | 81 | - | 110 | 91 | 86 | 92 | 92 | 110 |
| TS150 | 110 | 90 | 90 | 90 | 90 | 80 | - | 90 | 85 | 80 | 70 | 70 |
| TS151 | 110 | 110 | 110 | 110 | 110 | 110 | 110 | 110 | 110 | 110 | 110 | 110 |
| TS153 | 101 | 105 | 83 | 87 | 90 | 110 | 110 | 110 | 89 | 90 | 86 | 110 |
| TS154 | 90 | 90 | 80 | 75 | 80 | 110 | 96 | 95 | 90 | 85 | 80 | 110 |
|  |  |  |  |  |  |  |  |  |  |  |  |  |
|  |  |  |  |  |  |  |  |  |  |  |  |  |
|  | **LDL right ear [dB HL]** | | | | | | **LDL left ear [dB HL]** | | | | | |
| Freq [kHz] | 0.25 | 0.5 | 1 | 2 | 4 | 6 | 0.25 | 0.5 | 1 | 2 | 4 | 6 |
| Patients with Tinnitus | | | | | | | | | | | | |
| TS004 | - | 80 | 75 | 80 | 75 | 70 | - | 85 | 80 | 80 | 80 | 70 |
| TS005 | - | 100 | 110 | 110 | 110 | 110 | - | 100 | 100 | 100 | 100 | 105 |
| TS010 | - | 110 | 105 | 110 | 110 | 95 | - | 105 | 95 | 90 | 95 | 95 |
| TS017 | - | 95 | 80 | 80 | 80 | 70 | - | 90 | 90 | 80 | 80 | 80 |
| TS020 | - | 85 | 85 | 80 | 80 | 75 | - | 80 | 80 | 75 | 70 | 70 |
| TS021 | - | 90 | 90 | 85 | 90 | 85 | - | 90 | 95 | 90 | 90 | 85 |
| TS031 | - | 80 | 80 | 85 | 85 | 75 | - | 75 | 75 | 80 | 85 | 65 |
| TS033 | - | 80 | 75 | 80 | 85 | 80 | - | 80 | 85 | 80 | 80 | 75 |
| TS036 | - | 100 | 95 | 85 | 90 | 105 | - | 85 | 85 | 90 | 85 | 95 |
| TS037 | - | 90 | 90 | 90 | 80 | 90 | - | 90 | 90 | 90 | 90 | 90 |
| TS040 | - | 70 | 70 | 65 | 65 | 65 | - | 75 | 70 | 60 | 55 | 50 |
| TS044 | - | 100 | 100 | 100 | 110 | 100 | - | 100 | 100 | 100 | 100 | 90 |
| TS048 | - | 40 | 40 | 30 | 25 | 30 | - | 45 | 35 | 30 | 25 | 30 |
| TS049 | - | 110 | 110 | 110 | 110 | 110 | - | 110 | 110 | 105 | 100 | 100 |
| TS050 | - | 70 | 70 | 65 | 55 | 50 | - | 55 | 60 | 65 | 50 | 35 |
| TS053 | - | 80 | 70 | 70 | 80 | 85 | - | 75 | 70 | 70 | 75 | 80 |
| TS054 | - | 100 | 95 | 90 | 100 | 100 | - | 110 | 110 | 95 | 95 | 110 |
| TS057 | - | 105 | 90 | 90 | 85 | 70 | - | 85 | 75 | 80 | 70 | 60 |
| TS059 | - | 80 | 80 | 80 | 80 | 80 | - | 80 | 80 | 80 | 90 | 80 |
| TS061 | - | 70 | 60 | 60 | 60 | 50 | - | 70 | 60 | 65 | 55 | 50 |
| TS062 | - | 70 | 60 | 60 | 60 | 50 | - | 70 | 60 | 65 | 55 | 50 |
| TS067 | - | 60 | 55 | 55 | 40 | 40 | - | 50 | 45 | 50 | 45 | 30 |
| TS068 | - | 70 | 70 | 80 | 90 | 70 | - | 80 | 70 | 100 | 90 | 80 |
| TS070 | - | 100 | 95 | 100 | 100 | 95 | - | 100 | 90 | 95 | 90 | 90 |
| TS073 | - | 95 | 100 | 90 | 90 | 85 | - | 100 | 95 | 90 | 90 | 95 |
| TS079 | 110 | 110 | 110 | 110 | 110 | 110 | 110 | 110 | 110 | 110 | 110 | 110 |
| TS080 | 110 | 110 | 110 | 110 | 110 | 110 | 110 | 110 | 110 | 110 | 110 | 110 |
| TS082 | 110 | 90 | 76 | 85 | 82 | 80 | 110 | 91 | 82 | 84 | 89 | 86 |
| TS085 | 100 | 95 | 75 | 89 | 85 | 80 | 95 | 90 | 80 | 90 | 80 | 69 |
| TS086 | 110 | 110 | 110 | 94 | 90 | 86 | 110 | 110 | 110 | 95 | 85 | 84 |
| TS087 | 110 | 110 | 110 | 110 | 110 | 110 | 110 | 110 | 99 | 110 | 105 | 110 |
| TS088 | - | 65 | 64 | 66 | 74 | 75 | - | 65 | 58 | 72 | 66 | 70 |
| TS090 | - | 79 | 80 | 70 | 50 | 66 | - | 80 | 70 | 66 | 66 | 66 |
| TS091 | 110 | 99 | 99 | 99 | 110 | 100 | 110 | 110 | 99 | 95 | 110 | 110 |
| TS094 | 110 | 99 | 89 | 88 | 80 | 100 | 110 | 99 | 89 | 89 | 88 | 98 |
| TS095 | 110 | 80 | 80 | 80 | 80 | 81 | 110 | 80 | 86 | 80 | 86 | 91 |
| TS097 | 85 | 90 | 95 | 90 | 87 | 80 | 82 | 100 | 90 | 90 | 79 | 79 |
| TS098 | 110 | 110 | 110 | 90 | 85 | 85 | 110 | 110 | 100 | 95 | 85 | 76 |
| TS102 | - | 85 | 81 | 85 | 69 | 75 | - | 70 | 70 | 70 | 65 | 64 |
| TS104 | - | 53 | 49 | 47 | 49 | 46 | - | 49 | 44 | 32 | 40 | 39 |
| TS105 | - | 63 | 56 | 54 | 53 | 47 | - | 63 | 59 | 63 | 57 | 57 |
| TS121 | 90 | 95 | 85 | 85 | 90 | - | 95 | 104 | 90 | 89 | 95 | 110 |
| TS125 | - | 66 | 56 | 63 | 57 | - | - | 76 | 69 | 76 | 71 | - |
| TS137 | 90 | 85 | 110 | 75 | 85 | 90 | 90 | 90 | 110 | 90 | 85 | 80 |
| TS140 | 90 | 90 | 70 | 70 | 80 | - | 90 | 90 | 80 | 80 | 80 | - |
| TS141 | 110 | 89 | 89 | 88 | 89 | 88 | - | 89 | 90 | 89 | 88 | 89 |
| TS147 | 75 | 80 | 80 | 75 | 68 | 61 | 69 | 74 | 61 | 71 | 59 | 59 |
|  |  |  |  |  |  |  |  |  |  |  |  |  |

**Supplementary Table 4.** Loudness discomfort levels (LDL) for the right and left ear at 0.25, 0.5, 1, 2, 4, and 6 kHz. LDL measurement was stopped at 110 dB HL to prevent ear damage.

## Supplementary Figures


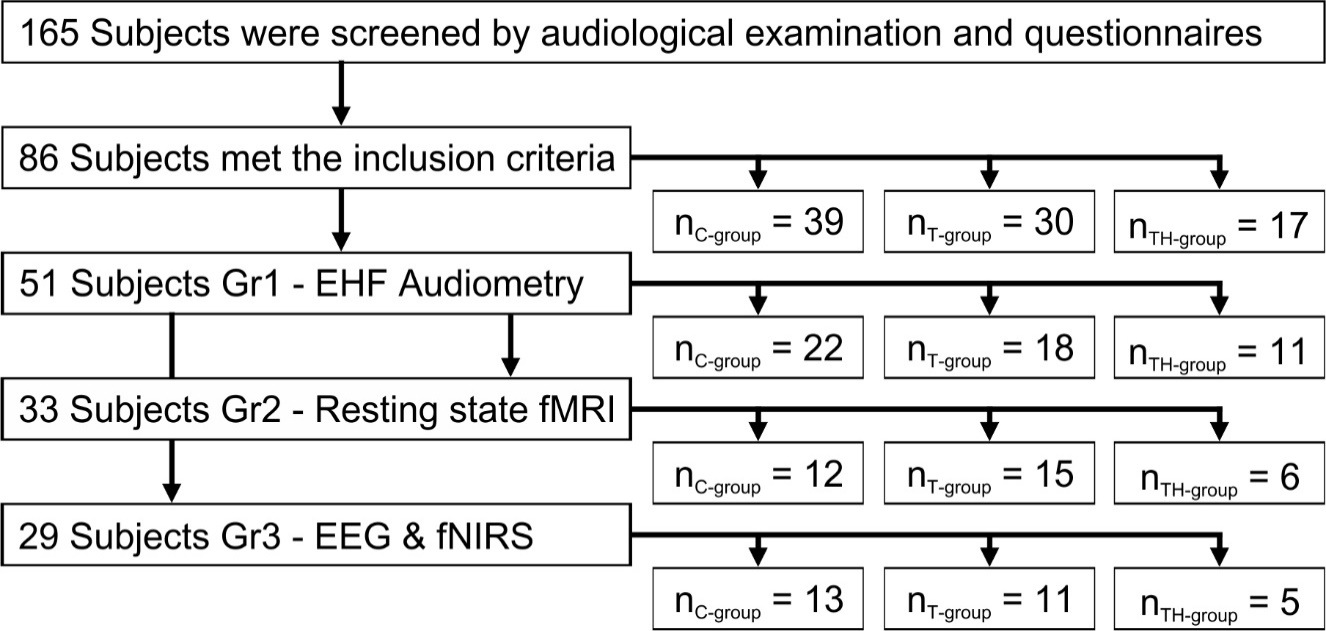


**Supplementary Figure 1.** Participant flow diagram providing an overview of the recruited subjects.


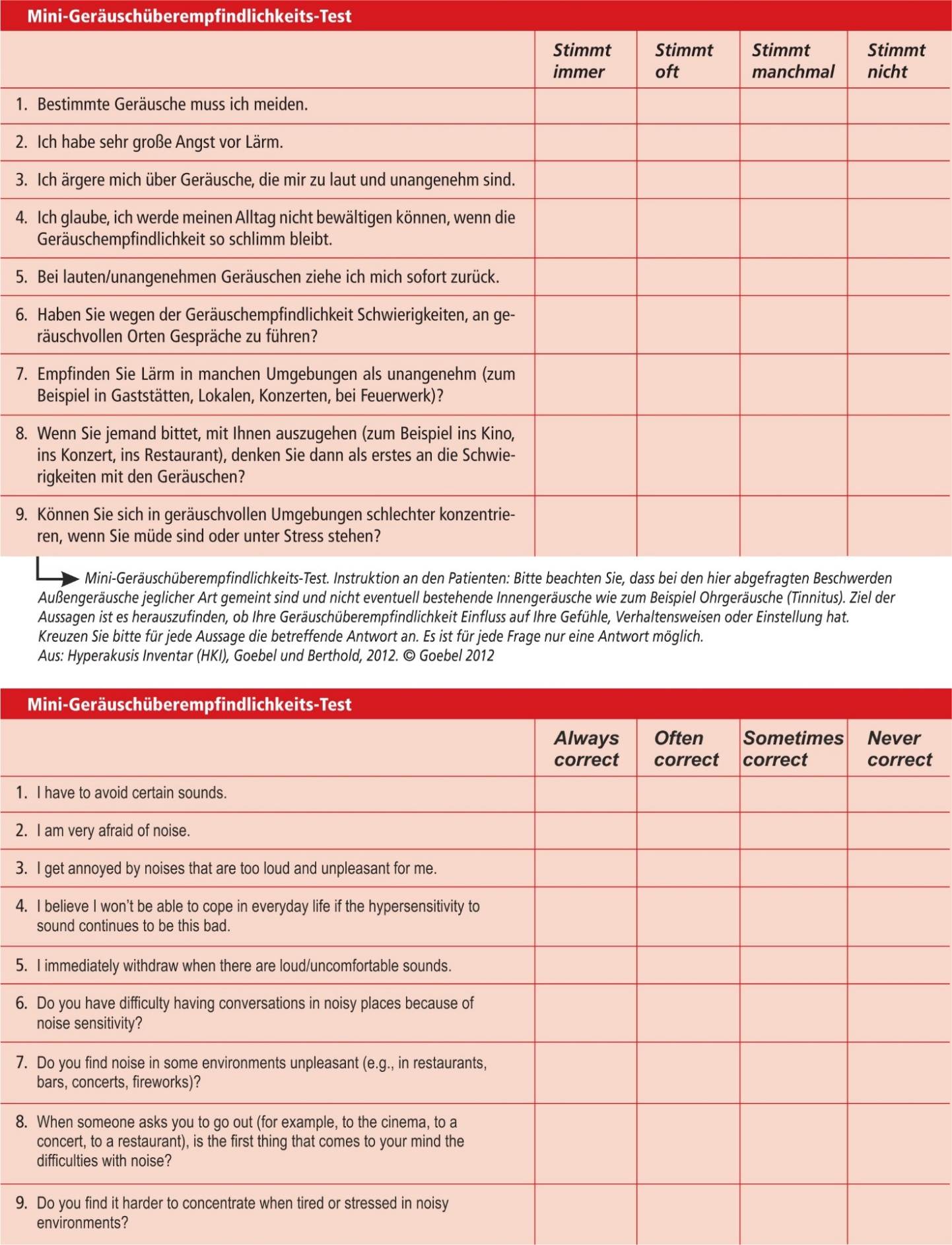


**Supplementary Figure 2.** The hyperacusis questionnaire is shown in the figure on top in the original German language and below in a version translated into English by the authors

**
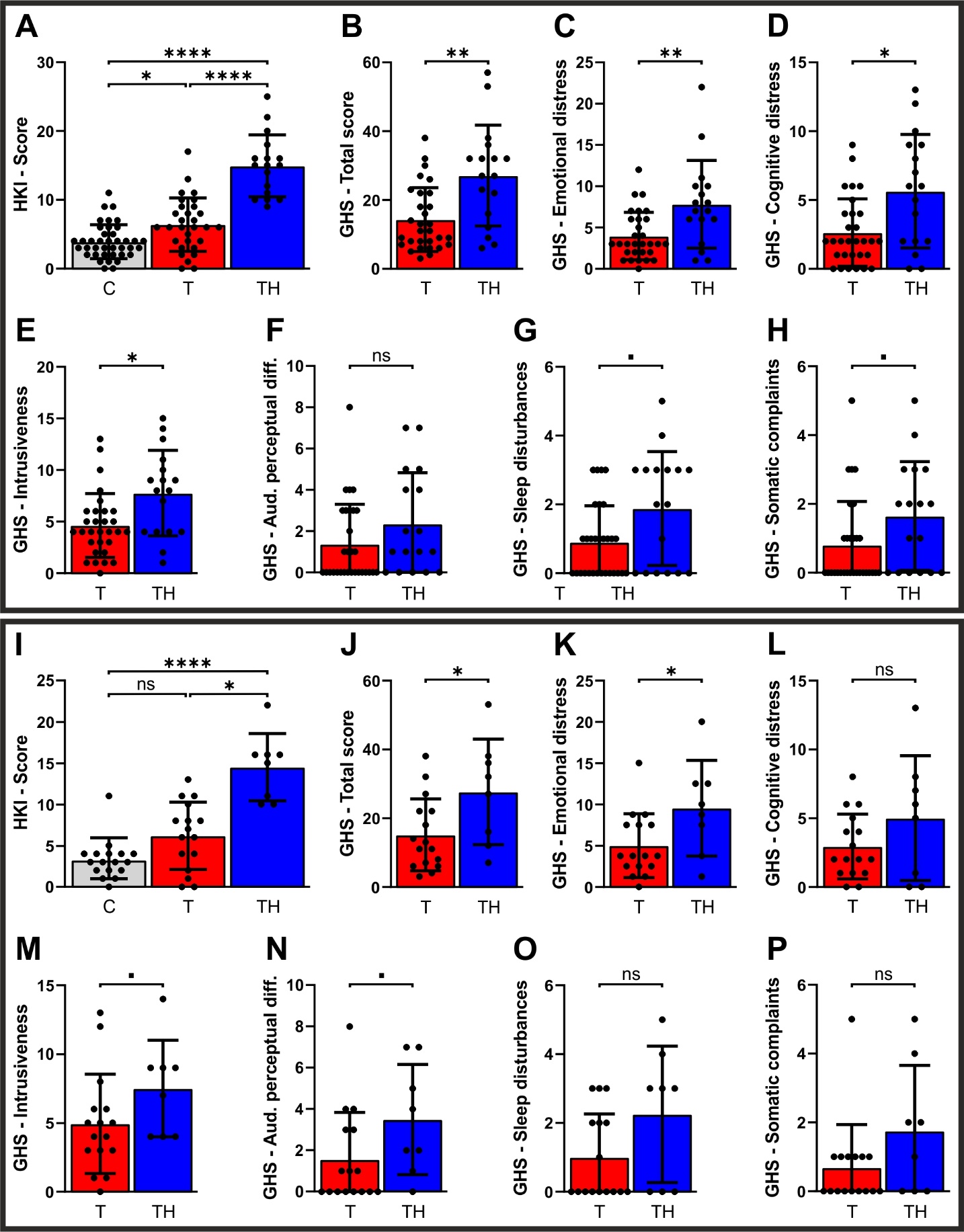
**

**Supplementary Figure 3.** Tinnitus and Hyperacusis Questionnaire Results: Bar charts represent mean ± SD for C-, T- (red), and TH-group (blue). Figures (A-H) show the total cohort and (I-P) a subset of EEG-measured Patients.


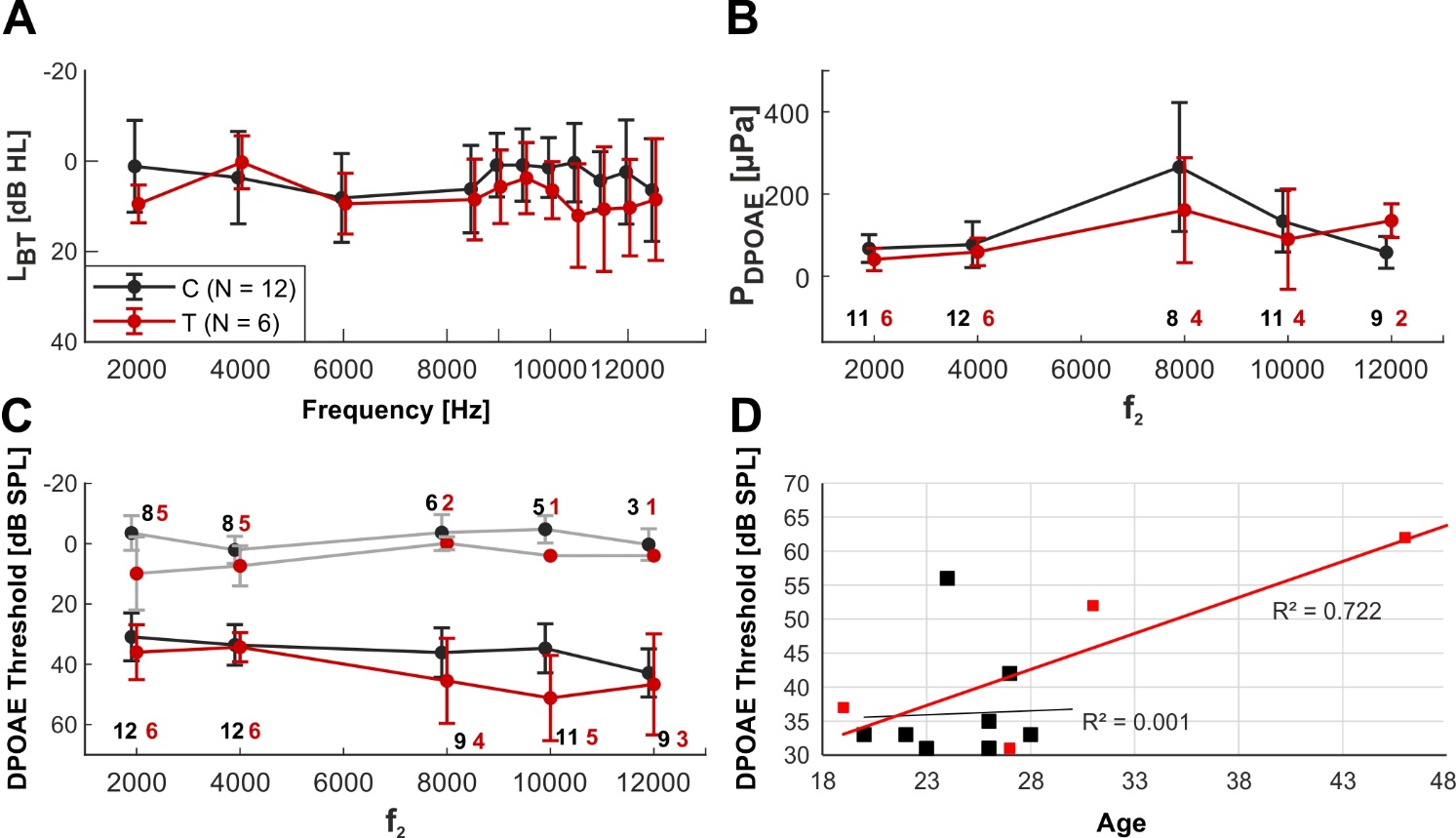


**Supplementary Figure 4.** **(A)** Mean behavioral thresholds were obtained with Békésy-Tracking audiometry (L_BT_) ± SD. **(B)** Mean pulsed DPOAE (pDPOAE) amplitudes (P_DPOAE_ at L_2_= 50 dB SPL) ± SD. **(C)** Mean pDPOAE thresholds (accepted DPOAE with lowest L_2_-stimulus) ± SD. In light gray mean estimated distortion-product thresholds (L_EDPT_) ± SD based on pDPOAE are shown in dB HL, L_EDPT_, pooled over all subjects and frequencies for ridge-based I/O functions derived from DPOAE level maps correlated linearly with behavioral thresholds (R² = 0.241, SD = 7.5 dB). **(D)** Age correlation for 8 kHz DPOAE Threshold of C- and T-group. All panels depict the controls in black and patients with tinnitus in red. In the insets in panels B-C, the number of contributing subjects is noted in the color corresponding to the subject groups tinnitus and controls, respectively.

.


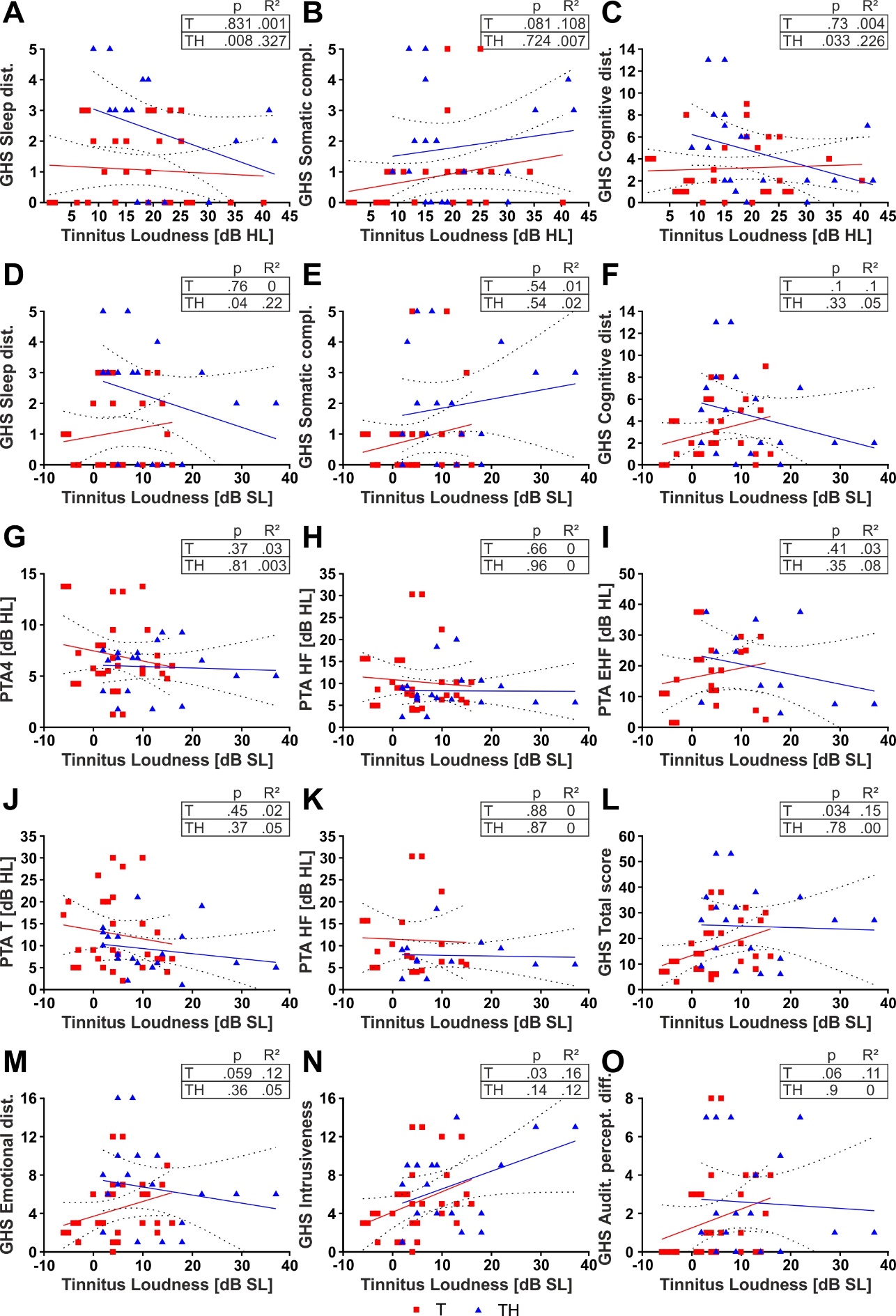


**Supplementary Figure 5.** Two‐tailed Spearman correlation for T- (red) and TH-group (blue) between Tinnitus loudness and **(A, D)** GHS Sleep disturbances, **(B, E)** GHS Somatic complaints, **(C, F)** GHS Cognitive distress, **(L)** GHS Total score, **(M)** GHS Emotional distress, **(N)** GHS Intrusiveness, **(O)** GHS Auditory perceptual difficulties. Two‐tailed Pearson correlation for T- (red) and TH-group (blue) between Tinnitus loudness and **(G)** PTA4, **(H)** PTA-HF, **(I)** PTA-EHF, **(J)** PTA-T, **(K)** PTA-HF in subjects with individual tinnitus in the range of 6-10 kHz. (**A-C**) show Tinnitus loudness in [dB HL] and (**D-O**) show Tinnitus loudness in [dB SL]. dB, decibel; GHS, Goebel and Hiller Score; HL, hearing level; SL, Sensation Levels.


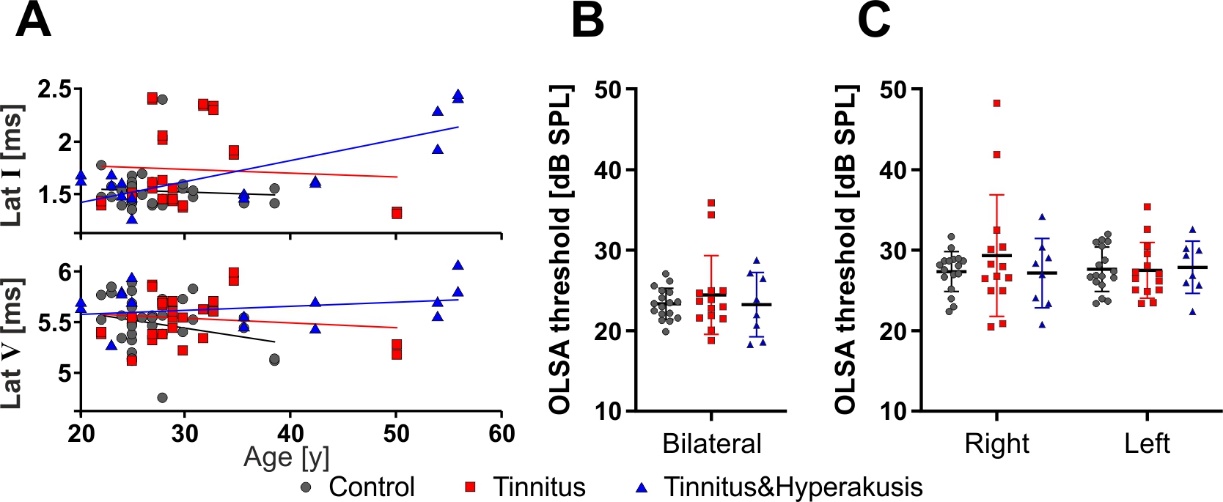


**Supplementary Figure 6.** The two-sided Pearson correlation between age and latency of ABR waves I (upper panel) and V (lower panel), respectively, are displayed for the C (gray), T (red), and TH (blue) groups at 85 dB nHL **(A)**. Speech perception is given as speech reception threshold at 50%-word recognition in dB HL for **(B)** bilateral stimulation without noise and **(C)** unilateral stimulation with contralateral 65 dB noise. Individual values, mean, and Standard deviation are shown. dB, decibel; HL, hearing level; OLSA, Oldenburger Satztest.


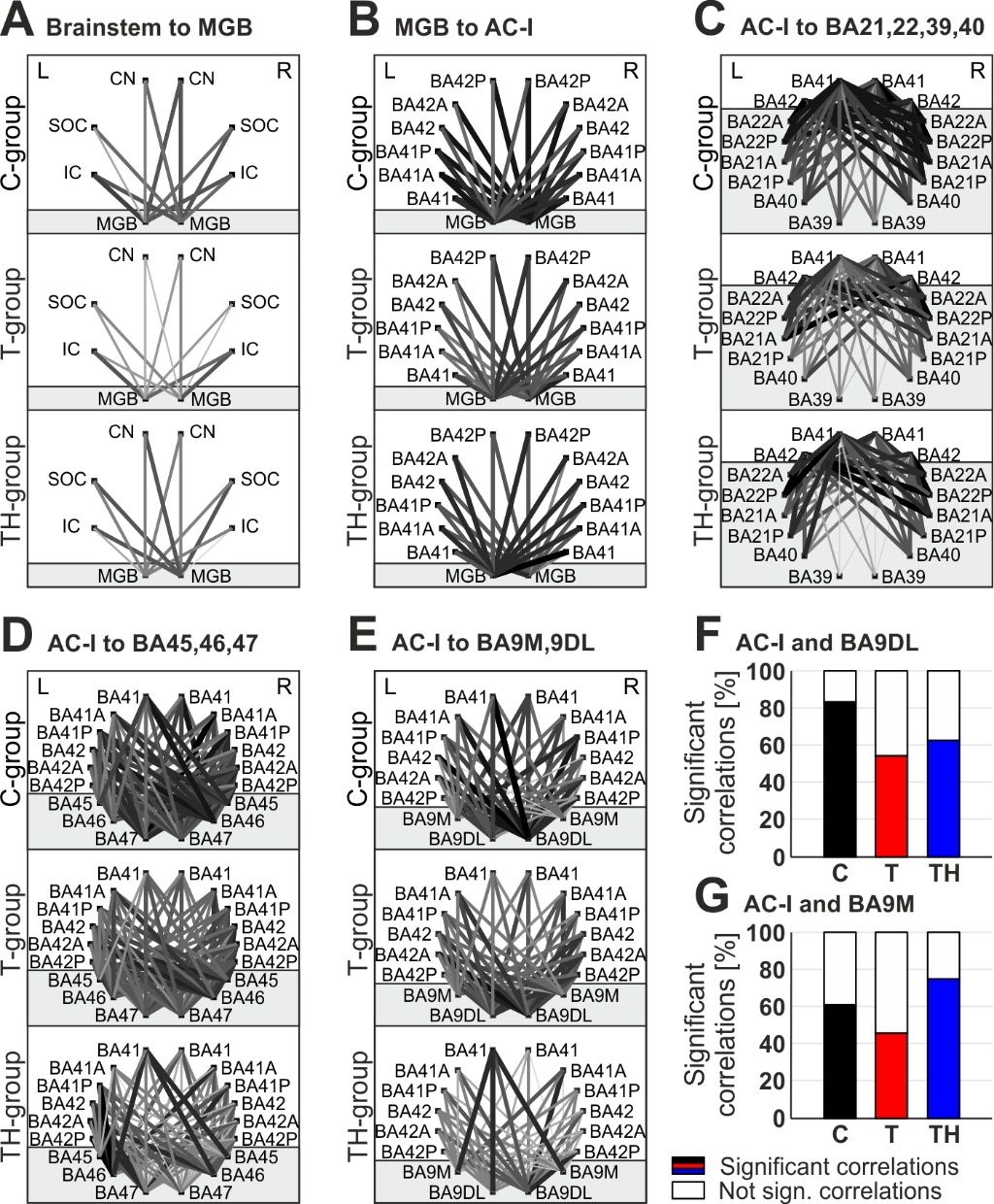


**Supplementary Figure 7.** The patterns in **(A-E)** show the frequency of significant positive (*p* < .05) rs-fMRI BOLD correlations between predefined ROIs for C- (*n* = 12, gray), T- (*n* = 15, red), and TH-group (*n* = 6, blue). The thickness and color of lines correspond to the correlation strength. Frequency of significant positive correlations between **(A)** subcortical regions and MGB **(B)** MGB and AC-I **(C)** AC-I and BA45, BA46, BA47 **(D)** AC-I and BA9M, BA9DL **(E)** AC-I and BA21, BA22, BA39, BA40. The boxplots **(F, G)** display the frequency of significant positive rs-fMRI BOLD correlations between predefined ROIs for C- (*n* = 12, gray), T- (*n* = 15, red), and TH-group (*n* = 6, blue). Fisher exact probability test show descriptive group differences in the frequency of significant positive (*p* < .05) rs-fMRI BOLD correlations between **(F)** AC-I-L and BA9DL (p = 0.122) and **(G)** AC-I-L and BA9M (p = 0.298). AC-I, primary auditory cortex; BA, Brodmann area; BOLD, blood oxygenation level depended; DL, dorsolateral; L, left hemisphere; M, medial; MGB, medial geniculate body; R, right hemisphere; ROI, region of interest.


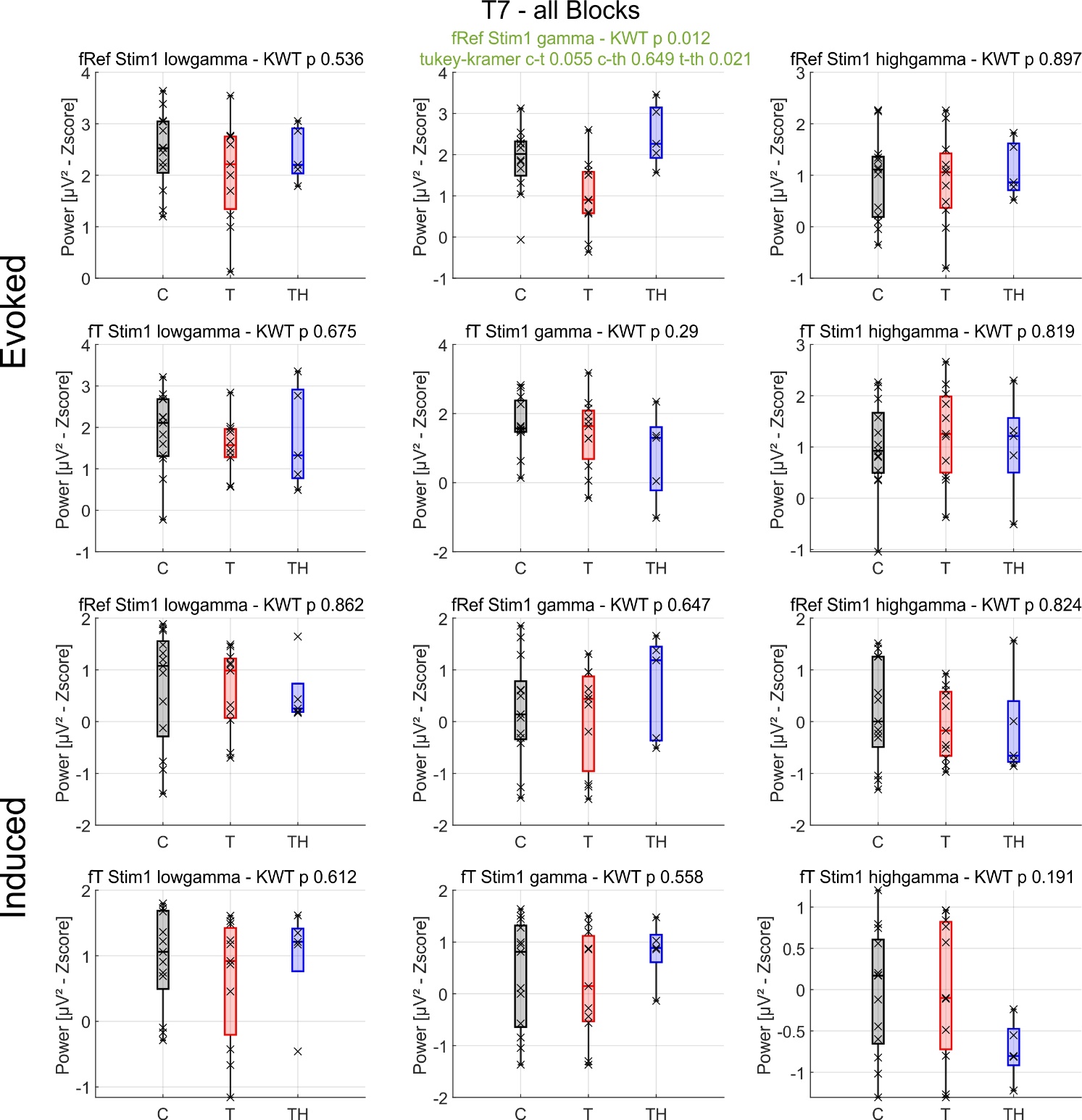


**Supplementary Figure 8.** Evoked and induced power of low-, mid-, and high-gamma in the T7 electrode position for the first stimulus in reference frequency (fRef) and individual tinnitus frequency (fTin): Bar charts represent the median and quartiles (box) for C- (gray), T- (red), and TH-group (blue). If the Kruskal-Wallis test shows a statistical trend (p < 0.1), Dunn’s multiple comparison tests are given in the Figure heading in green.


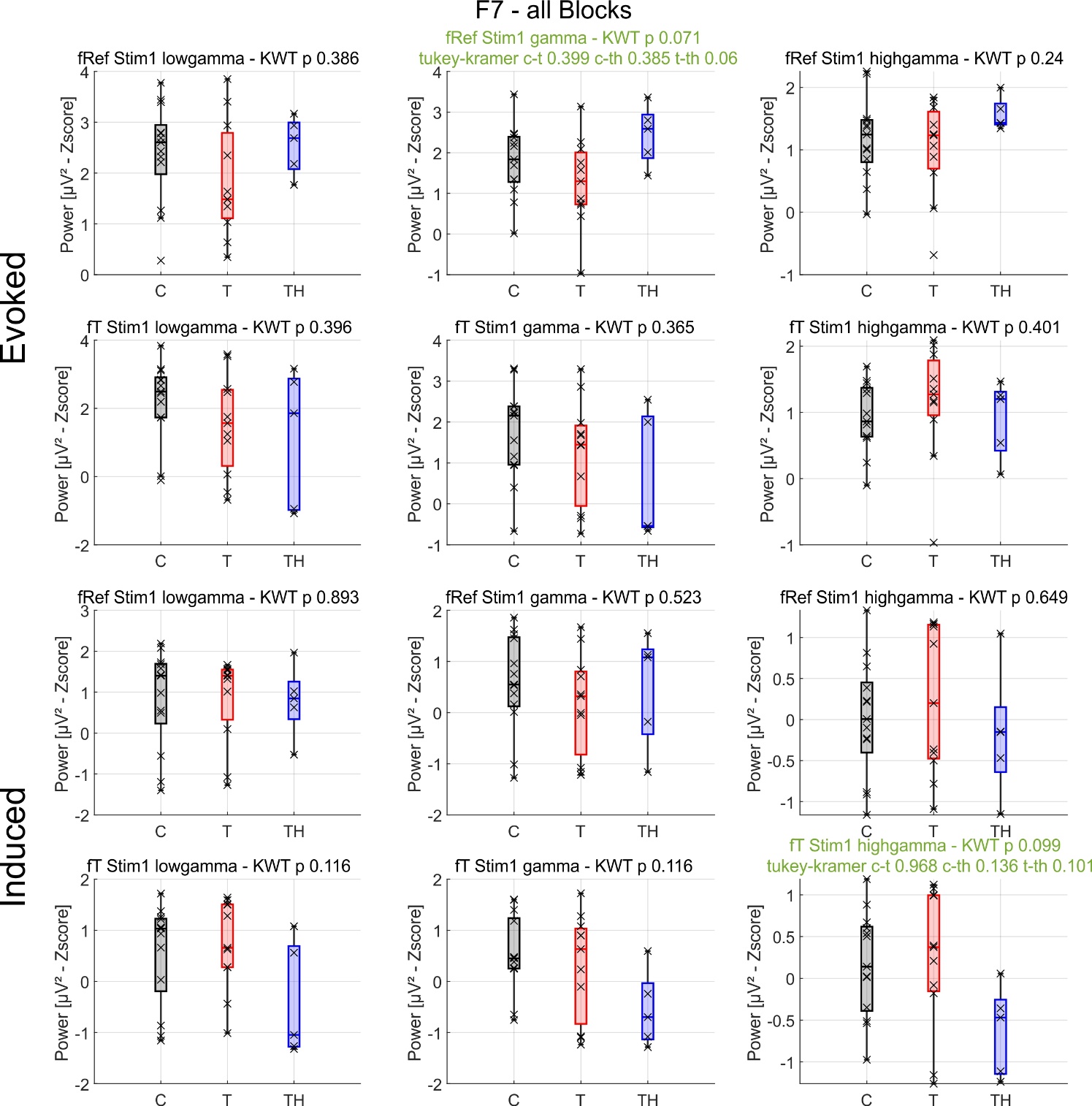


**Supplementary Figure 9.** Evoked and induced power of low-, mid-, and high-gamma in the F7 electrode position for the first stimulus in reference frequency (fRef) and individual tinnitus frequency (fTin): Bar charts represent the median and quartiles (box) for C- (gray), T- (red), and TH-group (blue). If the Kruskal-Wallis test shows a statistical trend (p < 0.1), Dunn’s multiple comparison tests are given in the Figure heading in green.


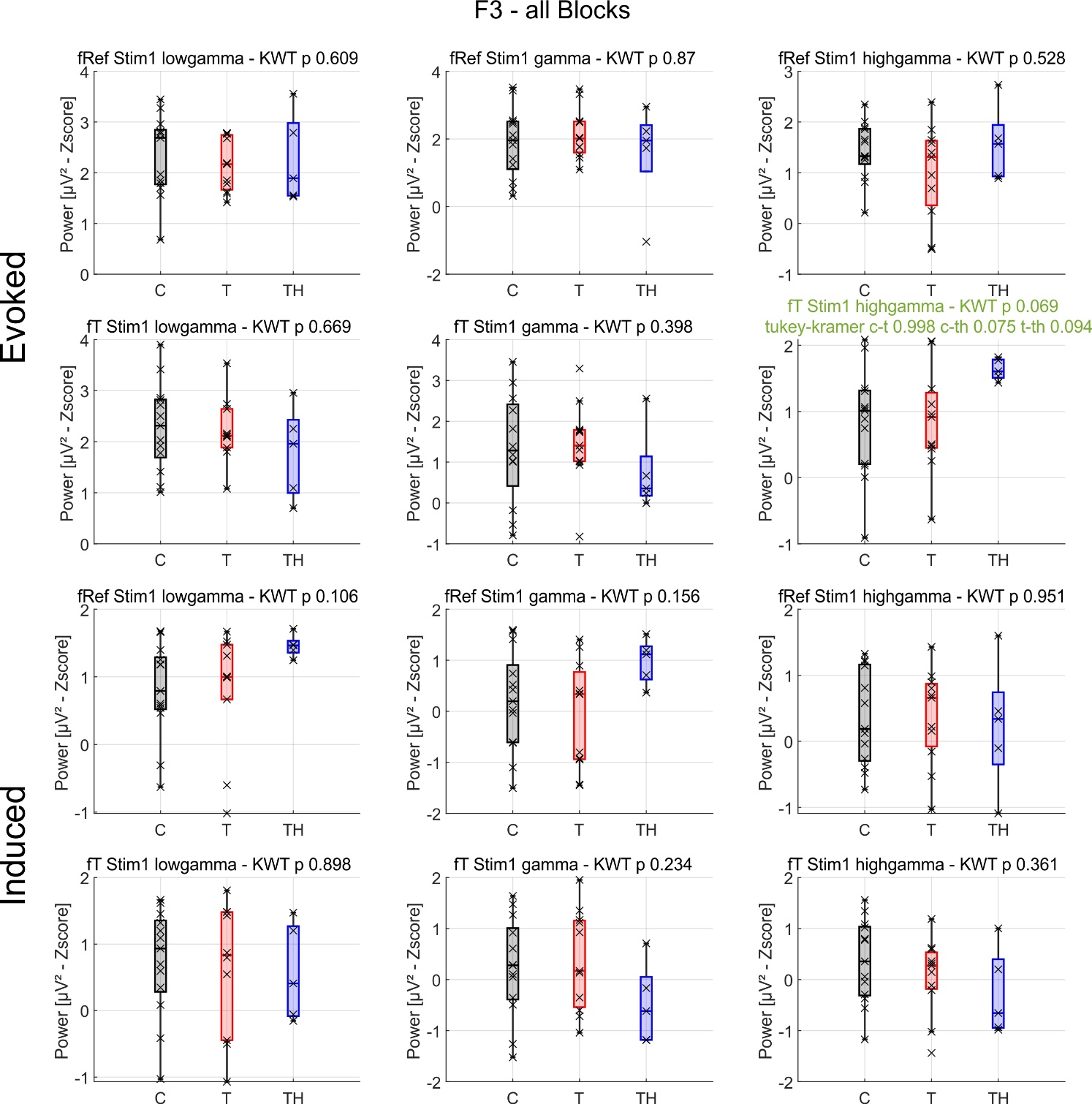


**Supplementary Figure 10.** Evoked and induced power of low-, mid-, and high-gamma in the F3 electrode position for the first stimulus in reference frequency (fRef) and individual tinnitus frequency (fTin): Bar charts represent the median and quartiles (box) for C- (gray), T- (red), and TH-group (blue). If the Kruskal-Wallis test shows a statistical trend (p < 0.1), Dunn’s multiple comparison tests are given in the Figure heading in green.


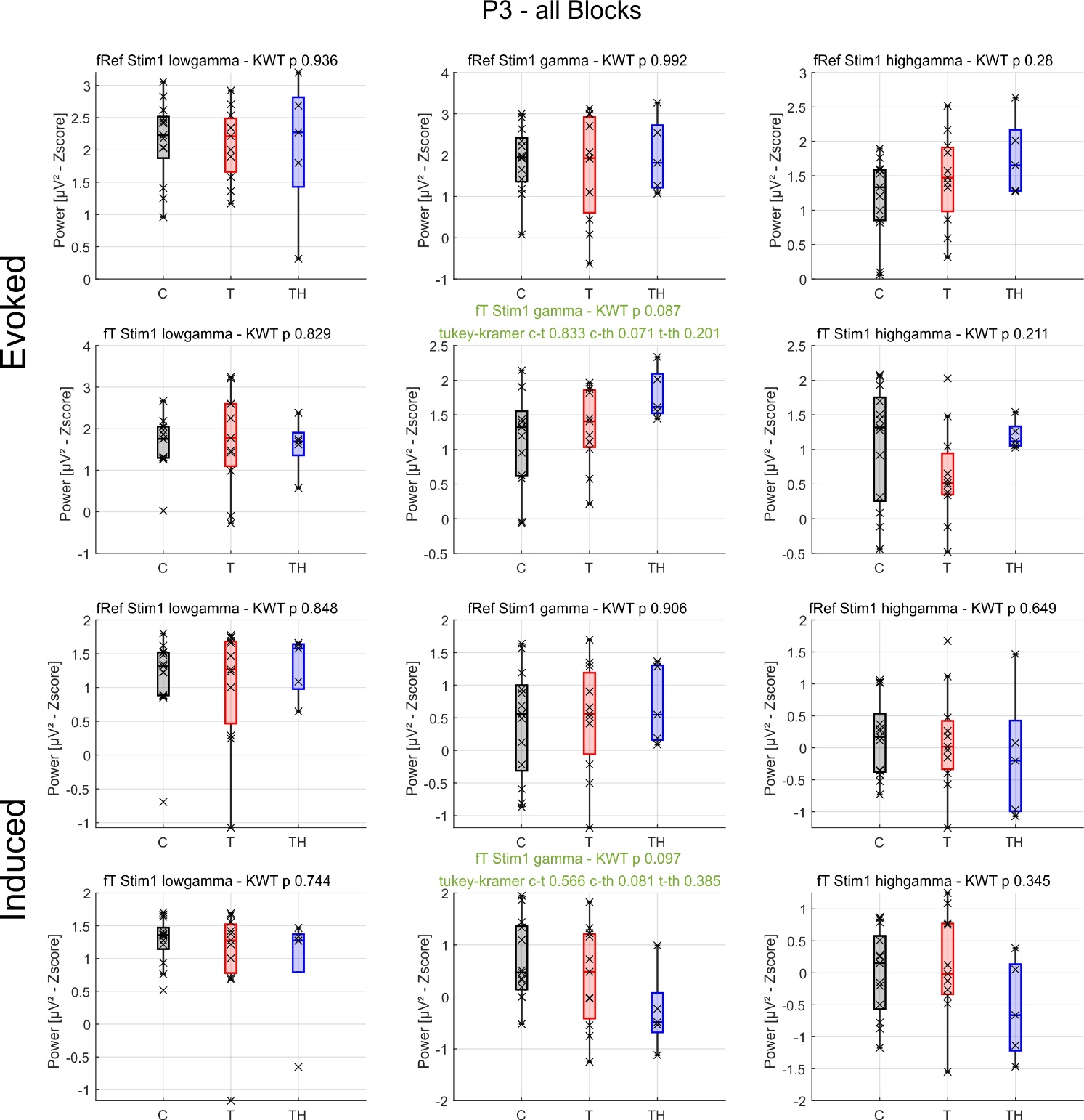


**Supplementary Figure 11.** Evoked and induced power of low-, mid-, and high-gamma in the P3 electrode position for the first stimulus in reference frequency (fRef) and individual tinnitus frequency (fTin): Bar charts represent the median and quartiles (box) for C- (gray), T- (red), and TH-group (blue). If the Kruskal-Wallis test shows a statistical trend (p < 0.1), Dunn’s multiple comparison tests are given in the Figure heading in green.


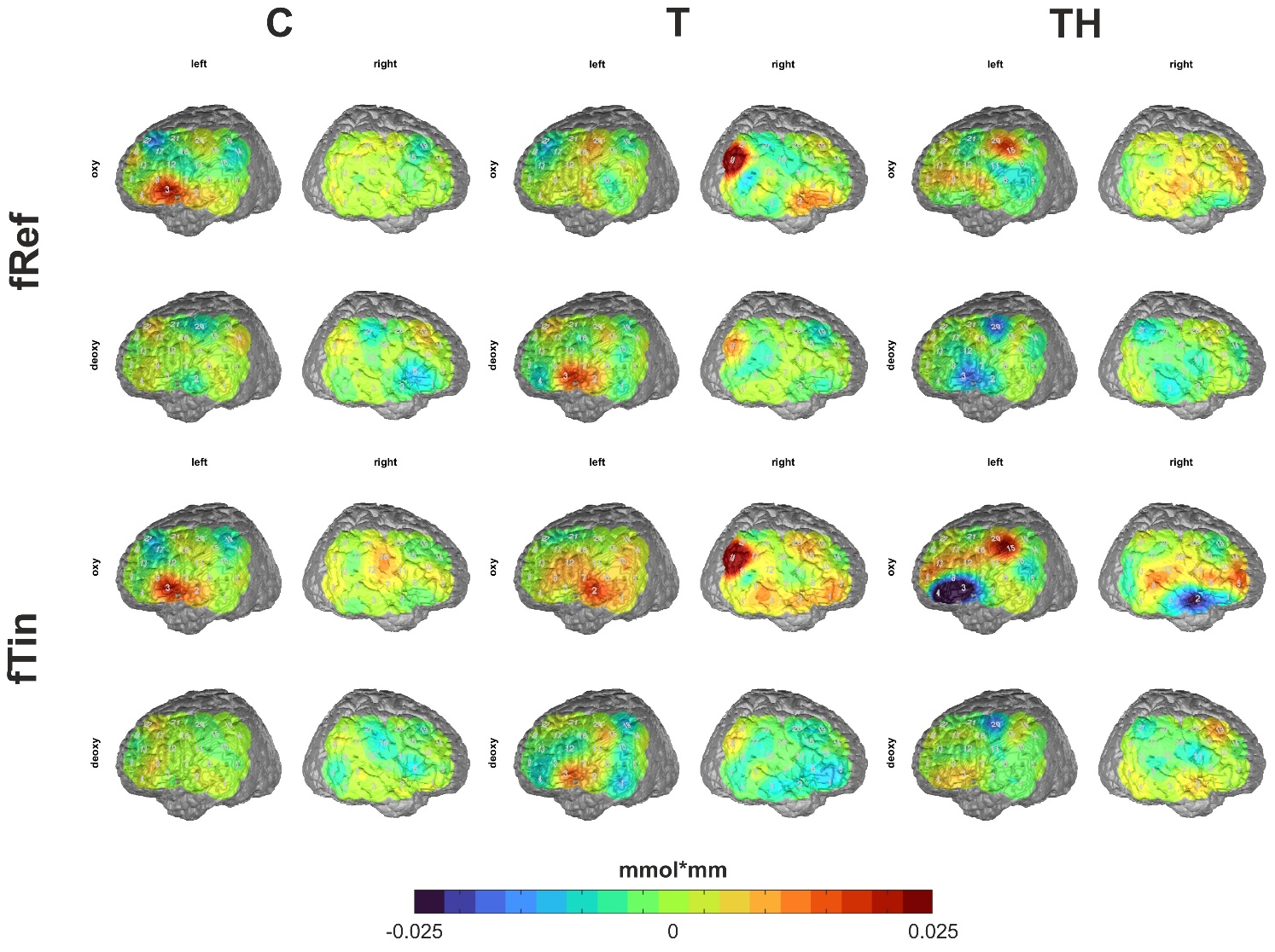


**Supplementary Figure 12.** Concentration changes of oxygenated and deoxygenated hemoglobin for the two 22-channel optode arrays covering the left and right fronto-temporo-parietal head areas.


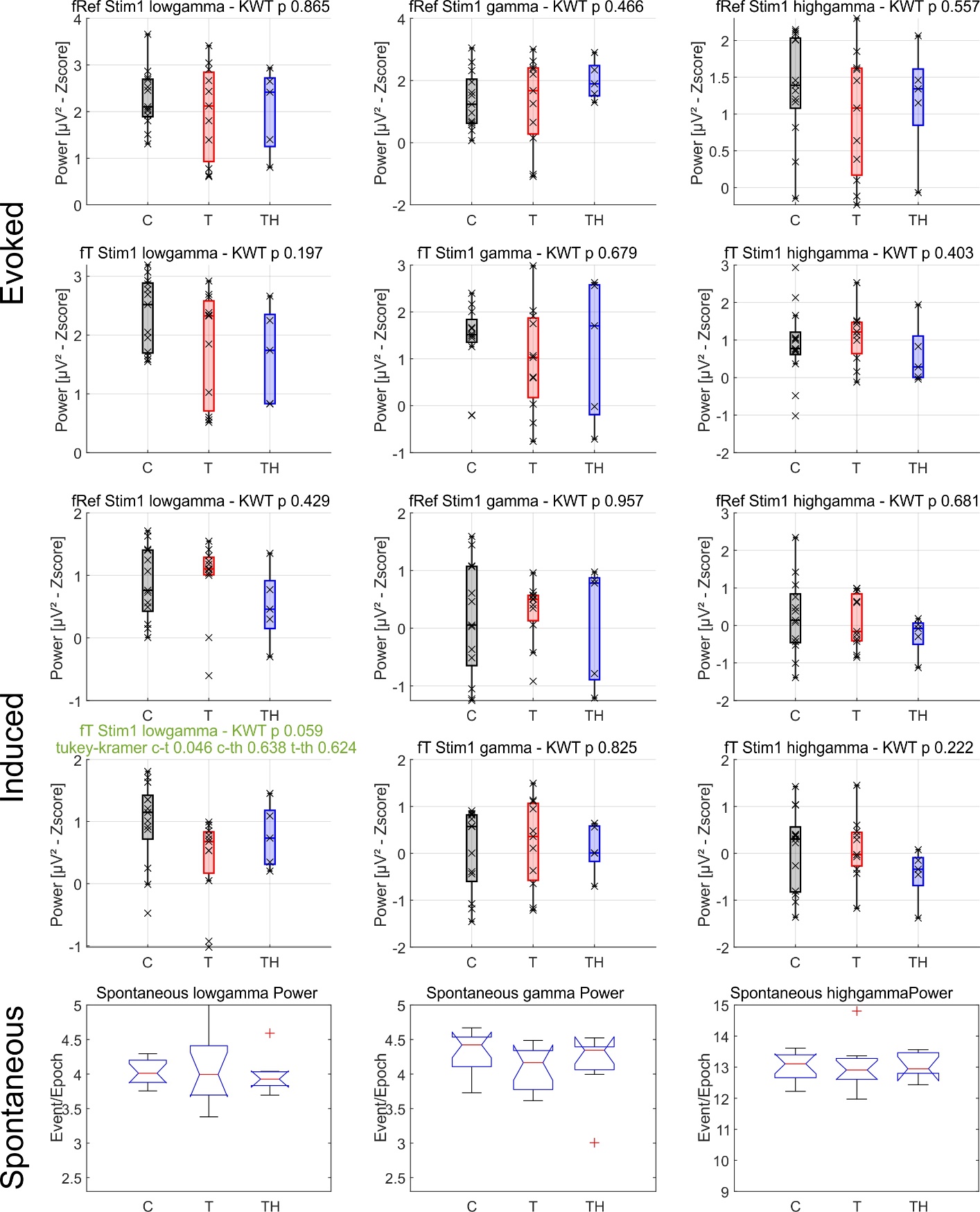


**Supplementary Figure 13.** Spontaneous, evoked, and induced power of low-, mid-, and high-gamma in the T8 electrode position for the first stimulus in reference frequency (fRef) and individual tinnitus frequency (fTin): Bar charts represent the median and quartiles (box) for C- (gray), T- (red), and TH-group (blue). If the Kruskal-Wallis test shows a statistical trend (p < 0.1), Dunn’s multiple comparison tests are given in the Figure heading in green.


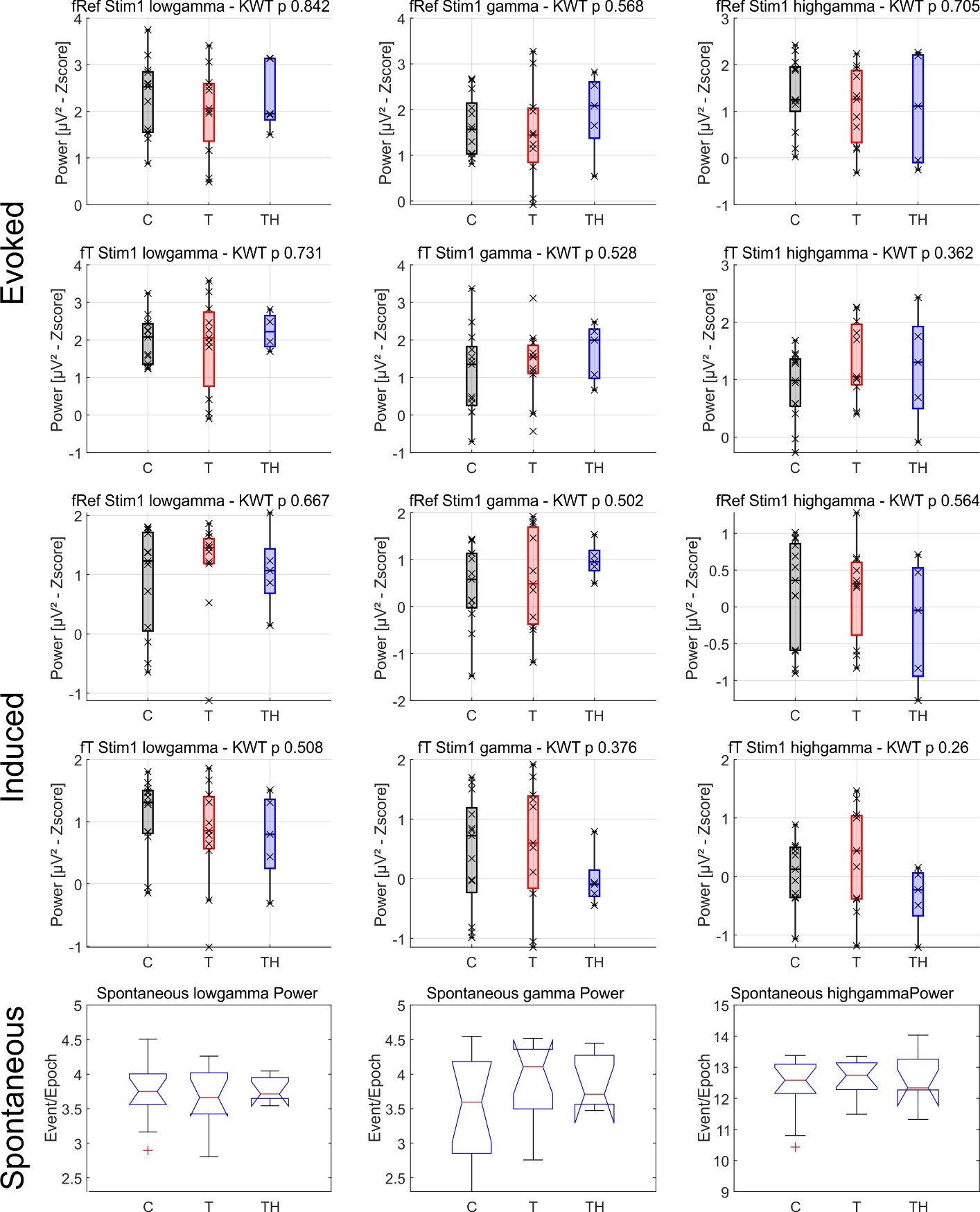


**Supplementary Figure 14.** Spontaneous, evoked, and induced power of low-, mid-, and high-gamma in the F8 electrode position for the first stimulus in reference frequency (fRef) and individual tinnitus frequency (fTin): Bar charts represent the median and quartiles (box) for C- (gray), T- (red), and TH-group (blue). If the Kruskal-Wallis test shows a statistical trend (p < 0.1), Dunn’s multiple comparison tests are given in the Figure heading in green.


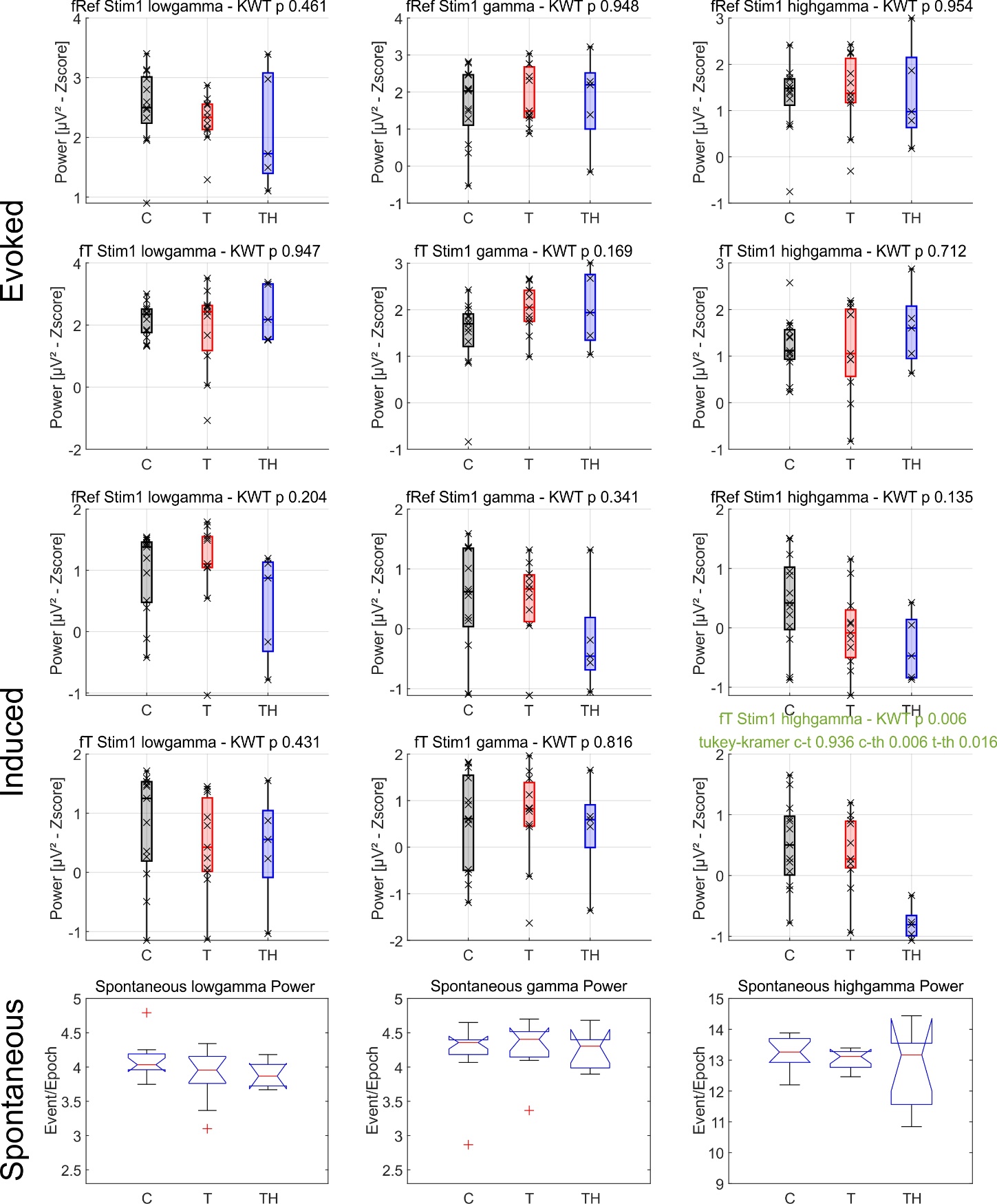


**Supplementary Figure 15.** Spontaneous, evoked, and induced power of low-, mid-, and high-gamma in the F4 electrode position for the first stimulus in reference frequency (fRef) and individual tinnitus frequency (fTin): Bar charts represent the median and quartiles (box) for C- (gray), T- (red), and TH-group (blue). If the Kruskal-Wallis test shows a statistical trend (p < 0.1), Dunn’s multiple comparison tests are given in the Figure heading in green.

**References**

Amunts, K., Mohlberg, H., Bludau, S., and Zilles, K. (2020). Julich-Brain: A 3D probabilistic atlas of the human brain's cytoarchitecture. *Science* 369(6506)**,** 988-992. doi: 10.1126/science.abb4588.

Bader, K., Dierkes, L., Braun, L.H., Gummer, A.W., Dalhoff, E., and Zelle, D. (2021). Test-retest reliability of distortion-product thresholds compared to behavioral auditory thresholds. *Hear Res* 406**,** 108232. doi: 10.1016/j.heares.2021.108232.

Berthold-Scholz, A. (2013). *Validierung von Selbsteinschätzungsinstrumenten (GÜF, Nelting und Finlayson, 2004 und HQ, Khalfa et al., 2022) in der Diagnostik der Geräuschüberempfindlichkeit-Entwicklung eines Hyperakusis-Inventar (HKI) zur Fremd- und Selbsteinschätzung.* Doktor der Medizin, Technische Universität München.

Fischer, A. (2013). Hyperakusis: Neues Screening-Instrument vorgestellt. *HNO Nachrichten* 43(3)**,** 38-38. doi: 10.1007/s00060-013-0111-x.

Frank, T., and Karlovich, R.S. (1975). Effect of contralateral noise on speech detection and speech reception thresholds. *Audiology* 14(1)**,** 34-43. doi: 10.3109/00206097509071721.

Goldstein, B., and Shulman, A. (1996). Tinnitus - Hyperacusis and the Loudness Discomfort Level Test - A Preliminary Report. *Int Tinnitus J* 2**,** 83-89.

Hiller, W., Goebel, G., and Rief, W. (1994). Reliability of self-rated tinnitus distress and association with psychological symptom patterns. *Br J Clin Psychol* 33 ( Pt 2)**,** 231-239.

Hofmeier, B., Wolpert, S., Aldamer, E.S., Walter, M., Thiericke, J., Braun, C., et al. (2018). Reduced sound-evoked and resting-state BOLD fMRI connectivity in tinnitus. *Neuroimage Clin* 20**,** 637-649. doi: 10.1016/j.nicl.2018.08.029.

Jafari, Z., Baguley, D., Kolb, B.E., and Mohajerani, M.H. (2022). A Systematic Review and Meta-Analysis of Extended High-Frequency Hearing Thresholds in Tinnitus With a Normal Audiogram. *Ear Hear* 43(6)**,** 1643-1652. doi: 10.1097/AUD.0000000000001229.

Khalfa, S., Dubal, S., Veuillet, E., Perez-Diaz, F., Jouvent, R., and Collet, L. (2002). Psychometric normalization of a hyperacusis questionnaire. *ORL J Otorhinolaryngol Relat Spec* 64(6)**,** 436-442. doi: 10.1159/000067570.

Lacadie, C.M., Fulbright, R.K., Rajeevan, N., Constable, R.T., and Papademetris, X. (2008). More accurate Talairach coordinates for neuroimaging using non-linear registration. *Neuroimage* 42(2)**,** 717-725. doi: 10.1016/j.neuroimage.2008.04.240.

Lancaster, J.L., Woldorff, M.G., Parsons, L.M., Liotti, M., Freitas, C.S., Rainey, L., et al. (2000). Automated Talairach atlas labels for functional brain mapping. *Hum Brain Mapp* 10(3)**,** 120-131. doi: 10.1002/1097-0193(200007)10:3<120::aid-hbm30>3.0.co;2-8.

Mai, J.K., Majtanik, M., and Paxinos, G. (2016). *Atlas of the human brain.* Amsterdam: Elsevier Ltd.

Muhlau, M., Rauschecker, J.P., Oestreicher, E., Gaser, C., Rottinger, M., Wohlschlager, A.M., et al. (2006). Structural brain changes in tinnitus. *Cereb Cortex* 16(9)**,** 1283-1288. doi: 10.1093/cercor/bhj070.

Nelting, M., Rienhoff, N.K., Hesse, G., and Lamparter, U. (2002). [The assessment of subjective distress related to hyperacusis with a self-rating questionnaire on hypersensitivity to sound]. *Laryngorhinootologie* 81(5)**,** 327-334. doi: 10.1055/s-2002-28342.

Peng, F., Xiang, Y., Xu, H., Yin, Q., Li, J., and Zou, Y. (2021). Systematic review and meta-analysis of extended high-frequency audiometry in tinnitus patients. *Ann Palliat Med* 10(12)**,** 12129-12139. doi: 10.21037/apm-21-3060.

Song, Z., Wu, Y., Tang, D., Lu, X., Qiao, L., Wang, J., and Li, H. (2021). Tinnitus Is Associated With Extended High-frequency Hearing Loss and Hidden High-frequency Damage in Young Patients. *Otol Neurotol* 42(3)**,** 377-383. doi: 10.1097/MAO.0000000000002983.

Vetesnik, A., Turcanu, D., Dalhoff, E., and Gummer, A.W. (2009). Extraction of sources of distortion product otoacoustic emissions by onset-decomposition. *Hear Res* 256(1-2)**,** 21-38. doi: 10.1016/j.heares.2009.06.002.

Yan, C.G., Wang, X.D., Zuo, X.N., and Zang, Y.F. (2016). DPABI: Data Processing & Analysis for (Resting-State) Brain Imaging. *Neuroinformatics* 14(3)**,** 339-351. doi: 10.1007/s12021-016-9299-4.

Zelle, D., Bader, K., Dierkes, L., Gummer, A.W., and Dalhoff, E. (2020). Derivation of input-output functions from distortion-product otoacoustic emission level maps. *J Acoust Soc Am* 147(5)**,** 3169. doi: 10.1121/10.0001142.

Zelle, D., Dalhoff, E., and Gummer, A.W. (2017). Comparison of time-domain source-separation techniques for short-pulse distortion-product otoacoustic emissions. *J Acoust Soc Am* (142:EL544.).
